# Effects of dapagliflozin on blood volume status and vascular outcomes in clinically stabilized heart failure patients after an acute decompensated heart failure event (DAPA-VOLVO study): Protocol of a double-blind randomized controlled clinical trial

**DOI:** 10.1371/journal.pone.0325668

**Published:** 2025-07-02

**Authors:** Konstantinos Bitos, Natallia Laptseva, Thomas Haider, Valentina A. Rossi, Matthias P. Nägele, Jens Barthelmes, Frank Ruschitzka, Isabella Sudano, Andreas J. Flammer

**Affiliations:** 1 Heart Failure and Heart Transplantation Unit, Department of Cardiology, University Heart Center Zurich, Zurich, Switzerland; 2 Center for Translational and Experimental Cardiology, University Heart Center Zurich, Schlieren, Switzerland; 3 University of Zurich, Zurich, Switzerland; Semnan University of Medical Sciences and Health Services, IRAN, ISLAMIC REPUBLIC OF

## Abstract

**Introduction:**

Heart failure (HF) is among the most prevalent health issues worldwide and is associated with high mortality. Adequate decongestion remain the main clinical challenge in HF management. Sodium glucose cotransporter-2 inhibitors (SGLT-2i) have been recently introduced as a new treatment option in patients with HF irrespective of left ventricular ejection fraction. Although the favorable effects of SGLT-2i are profoundly evident, the underlying mechanisms are not yet well understood. The aim of this study is to provide novel insights into the effects of dapagliflozin, a SGLT-2i with proven cardiovascular benefit, on blood volume profile and vascular function in HF patients who had a recent event of acute decompensated heart failure (ADHF).

**Methods:**

Eighty adult patients with diagnosis of de novo or chronic HF (NYHA class II-IV), clinically stabilized after an ADHF event and with preserved renal function, who were not on treatment with SGLT-2i, are aimed to be included. The patients are randomized with 1:1 allocation to either dapagliflozin 10 mg p.o. once daily or placebo in addition to guideline-directed medical therapy. The primary outcome is the mean change in plasma volume status (PVS) in the dapagliflozin group compared to placebo. PVS is assessed via optimized carbon monoxide rebreathing technique, a reliable and safe method to measure total hemoglobin mass and to estimate blood volume profile, i.e., blood volume, plasma volume and red blood cell volume. Secondary outcomes include differences between the two study groups regarding blood volume profile, micro- and macro-vascular function assessed by retinal vessel analysis and flow-mediated vasodilation, respectively, changes in body water distribution, quality of life, exercise capacity, echocardiographic and laboratory parameters.

**Ethics and dissemination:**

The study has been approved by the Cantonal Ethics Committee Zurich (BASEC-Nr.:2020−01920, Swissmedic-Nr.:2020DR4175) and has been registered at www.ClinicalTrials.gov‌ (NCT04869124). The results will be published in a peer-reviewed medical journal.

## 1. Introduction

Heart failure (HF) is a major health issue worldwide. Fluid volume overload and congestion, frequently combined with anemia [1] and red blood cell volume deficit [2], are central pathophysiological issues in patients with HF leading to increased hospitalization rates and mortality [3]. Functional disturbances of the cardio-renal-axis are also supposed to play a key role in this process [4].

Sodium-glucose cotransporter-2 inhibitors (SGLT-2i) were shown to be cardio- and reno-protective [5]. Based on data from hallmark randomized clinical trials [6–9], SGLT-2i have been included within standard medical treatment of all HF patients. As emphasized in the recent update of the guidelines of European Society of Cardiology [10,11], SGLT-2i are now recommended in HF patients across the entire spectrum of left ventricular ejection fraction (LVEF) in order to reduce the risk of HF hospitalization or cardiovascular death.

The beneficial effects of SGLT-2i on blood volume balance and erythropoiesis and subsequent amelioration of systemic hemodynamics, vascular function and oxygen transport, all resulting in improved tissue oxygen supply, have emerged as one mechanistic hypothesis to explain the cardio-reno-protective effects of dapagliflozin in particular [12]. Thus, treatment with SGLT-2i might be particularly beneficial in clinically compensated HF patients after an acute decompensated heart failure (ADHF) event. These HF patients frequently present with persistent blood volume imbalance with plasma volume overload and thus a higher risk of deteriorating HF and re-hospitalization [13], especially during the ‘vulnerable’ phase within the first 1–3 months after clinical discharge [14]. Therefore, reaching euvolemia after an ADHF event is the cornerstone of guideline-directed medical therapy (GDMT). Empagliflozin, another SGLT-2i with proven cardiovascular benefit, has been shown to have a positive effect on cardiovascular structure and function compared to placebo in non-diabetic patients with HF with reduced ejection fraction by significantly reducing epicardial adipose tissue and arterial stiffness [15]. Although dapagliflozin has been also shown to improve cardiovascular outcomes in HF patients, irrespective of LVEF [16], the exact mechanisms contributing to those favorable outcomes are still poorly understood. This study aims to provide a better insight to how dapagliflozin contributes to blood volume balance and to which extent improves the micro- and macro-vascular function, when compared to placebo.

### 1.1. Hypothesis and objectives

The primary study objective is whether dapagliflozin, on top of optimal GDMT, improves plasma volume status (PVS), assessed via optimized carbon monoxide rebreathing technique (OpCOT), in clinically stabilized HF patients after an ADHF event. PVS has been shown to be predictive for clinical outcomes [17–19] and seems to have important prognostic implications in HF patients and in patients hospitalized for an ADHF. Moreover, OpCOT is a highly accurate, investigator-independent and reliable method for measuring total hemoglobin mass [20,21], which is needed to calculate intravascular volumes, and has been proven to be safe and feasible in HF patients [22].

The main secondary study objectives are the effects of dapagliflozin on blood volume profile, i.e., blood volume, plasma volume and red blood cell volume, micro- and macro-vascular function as well as on total body water distribution. The study also seeks to determine whether the effects of dapagliflozin on blood volume profile and vascular function translate to functional improvements in terms of exercise capacity, health-related quality of life and quality of sleep. The effects of dapagliflozin on various echocardiographic parameters and biomarkers will be also assessed.

## 2. Materials and methods

### 2.1. Study design

**Fig 1:**
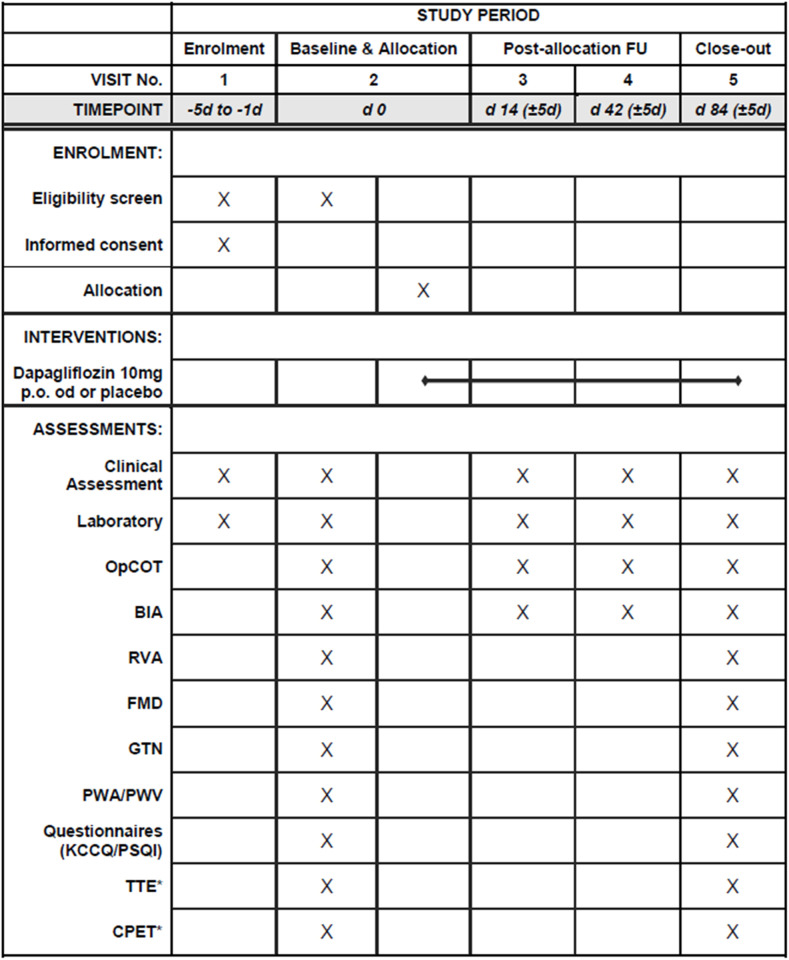
Study schedule. *Performed only by participation in the advanced study program. OpCOT: optimized carbon monoxide rebreathing technique, BIA: bioelectrical impedance, RVA: retinal vessel analysis, FMD: flow-mediated vasodilatation, GTN: vasodilatation after glycerol trinitrate sublingual, PWA: pulse wave analysis, PWV: pulse wave velocity, KCCQ: Kansas City Cardiomyopathy Questionnaire, PSQI: Pittsburgh Sleep Questionnaire Index, TTE: transthoracic echocardiography, CPET: cardiopulmonary exercise testing.

**Fig 2:**
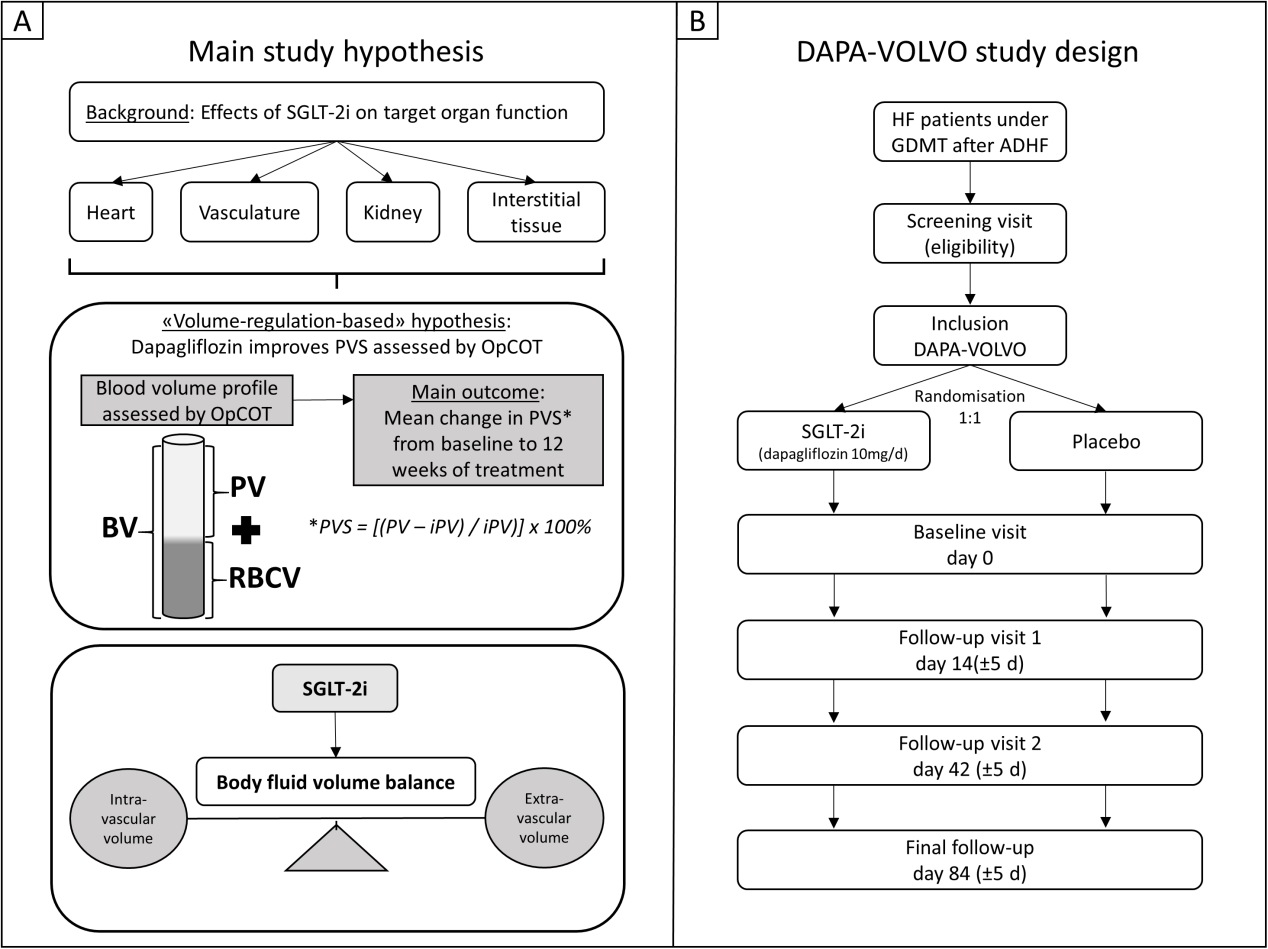
Main study objective hypothesis and DAPA-VOLVO study design. Panel A: Main study hypothesis. The sodium-glucose co-transporter 2 inhibitor (SGLT-2i) dapagliflozin, on top of guideline-directed medical therapy (GDMT) may lead to improvement of plasma volume status (PVS) from baseline to 12 weeks of treatment compared to placebo in patients with heart failure. Favorable effects on different target organs of fluid volume regulation including heart, kidney, vasculature and interstitial tissue support the hypothesis that dapagliflozin may improve body fluid volume balance by shifting the PVS towards ideal values (iPV: ideal plasma volume). OpCOT: optimized carbon monoxide rebreathing technique, BV: blood volume, PV: plasma volume, RCBV: red blood cell volume. Panel B: DAPA-VOLVO study design. HF: heart failure, ADHF: acute decompensated heart failure.

The current study is a randomized, double-blind, placebo-controlled parallel-group trial, evaluating the superiority of dapagliflozin, added to optimal GDMT, on blood volume profile regulation in stabilized HF patients after an ADHF event compared to placebo, across a 12-week treatment period (Fig 1). Eligible patients are randomized to either dapagliflozin (10 mg p.o. once daily) treatment group or placebo group with 1:1 allocation ratio using block randomization (Fig 2). Randomization is performed with a random computerized algorithm using the secuTrial® software system [23] via interactive response technology (IRT). The investigator contacts the IRT after confirming that the patient fulfils all the inclusion and none of the exclusion criteria. The IRT assigns a randomization number to the patient, which is used to link the patient to a treatment arm and specifies a unique medication number for the package of investigational treatment to be dispensed to the patient. Patients, investigator staff and data analysts are blinded to the identity of the treatment. The study protocol has been approved by the Cantonal Ethics Committee Zurich in Switzerland (BASEC-Nr.: 2020−01920, Swissmedic Nr.: 2020DR4175) and has been registered at www.ClinicalTrials.gov (NCT04869124). The original study protocol can be found in the supporting information material (S1 File).

### 2.2. Study population

Adult patients with a diagnosis of de novo or chronic HF, who have been clinically stabilized after hospitalization or ambulatory health care because of an ADHF (congestive) event, are not on treatment with SGLT-2i and have preserved renal function defined as estimated glomerular filtration rate (eGFR) ≥30 ml/min/1.73m^2^, are enrolled in this study. The detailed inclusion and exclusion criteria are shown in Table 1.

**Table 1:** Eligibility criteria.

Inclusion criteria
Age ≥ 18 years
Documented diagnosis of de novo or chronic heart failure (NYHA class II-IV) and clinically stabilized (considered for hospital discharge) after hospitalization or ambulatory care because of an acute decompensated (congestive) heart failure event
Preserved renal function (eGFR ≥ 30 mL/min/1.73 m^2^ according to CKD-EPI formula)
**Exclusion criteria**
Contraindications to SGLT2-inhibitor, e.g., known hypersensitivity or allergy
Receiving therapy with an SGLT2-inhibitor within 8 weeks prior to enrolment or previous intolerance of an SGLT2-inhibitor
Participation in another study with investigational drug within the 30 days preceding and during the present study
Type 1 diabetes mellitus
Symptomatic hypotension or systolic blood pressure < 90 mmHg at 2 out of 3 measurements either at screening or at baseline visit
Coronary revascularization (percutaneous coronary intervention because of STEMI or coronary artery bypass grafting) or valvular repair/replacement within 12 weeks prior to enrolment or planned to undergo any of these operations after randomization
Implantation of a CRT device within 12 weeks prior to enrolment or intent to implant a CRT device during 12 weeks of study observation period if indicated according to the actual ESC guidelines
Previous cardiac transplantation or implantation of a ventricular assistance device or similar device, or implantation expected after randomization
HF due to restrictive cardiomyopathy, active myocarditis, constrictive pericarditis, hypertrophic obstructive cardiomyopathy or uncorrected primary valvular disease
Symptomatic bradycardia or second or third degree heart block without a pacemaker
Severe (eGFR < 20 mL/min/1.73m^2^ by CKD-EPI), unstable or rapidly progressing renal disease
Women who are pregnant or breast feeding
Intention to become pregnant during the course of the study
Known or suspected non-compliance, drug or alcohol abuse
Inability to follow the procedures of the study, e.g., due to language problems, psychological disorders, dementia, etc. of the participant
Patients with severely restricted liver function
Patients with recurrent mycotic genital infections

NYHA: New York Heart Association, eGFR: estimated glomerular filtration rate, CKD-EPI: Chronic Kidney Disease Epidemiology collaboration, SGLT2: sodium-gluocose co-transporter 2. STEMI: ST-elevation myocardial infarction, CRT: cardiac resynchronization therapy, ESC: European Society of Cardiology.

### 2.3. Study setting & assessments

Patients are recruited at the University Hospital of Zurich and study assessments take place in the cardiology department’s clinical research facilities. After evaluation of eligibility in a screening visit and provided written informed consent, a baseline visit, and randomization are performed. If logistically feasible, screening and baseline visits are combined (Fig 1). Follow-up visits are scheduled in 14 (±5), 42 (±5) and 84 (±5) days after baseline. During the baseline visit and the last follow-up visit, a complete study assessment, as described below, is performed. During short-term follow-up visits (i.e., 14 and 42 days after baseline), clinical status, laboratory assessment and blood volume profile via OpCOT are evaluated. All study measurements are performed in the morning and in fasting state. All participants are requested to abstain from nicotine and coffee for at least 24 hours to avoid the possible effects of those substances on vascular function and blood volume profile. There are no restrictions on any guideline-recommended treatment adjustments (e.g., iron supplementation, up-titration of GDMT) after randomization and all changes on GDMT are documented.

By inclusion to the main study, patients are asked to participate in an advanced study program, including evaluation of echocardiographic parameters and exercise capacity. Those patients, who give written consent for participation to the advanced program, perform, additionally to the assessments mentioned above, a transthoracic echocardiogram and a cardiopulmonary exercise testing (CPET) at baseline visit and at the last follow-up visit.

The recruitment phase began on March 1, 2021 and ended on July 31, 2024. The detailed study flow chart, providing data on intention-to-treat and per-protocol analysis, as well as the reasons of discontinuation will be presented in the final study manuscript.

#### 2.3.1. Clinical assessment.

A study physician performs at each visit a complete clinical evaluation, including a detailed history, a review of current medication, including newly initiated GDMT, vital signs and weight measurement, and a standardized physical examination. During each follow-up visit, any adjustments to medical treatment made in the meantime, as well as interventions performed based on clinical recommendations, are thoroughly documented.

#### 2.3.2. Optimized carbon monoxide rebreathing technique (OpCOT).

The total hemoglobin mass (Hb_mass_) and the blood volume profile, consisting of blood volume (BV), plasma volume (PV) and red blood cell volume (RBCV), are determined via OpCOT. The patient is placed in supine position and a venous catheter (22G venflon, BD, USA) is inserted into an antecubital vein. Blood samples, including venous blood gas probe to measure the percentage of carboxyhemoglobin (COHb), the hemoglobin concentration (Hb) and the hematocrit (Hct), are taken. After a 10-minute resting period in supine position, blood pressure and oxygen saturation by pulse oximeter (SpO_2_) are measured, a suitable nose clip is placed and the patient is connected through a mouthpiece with breathing filter (Filter SafeStar 55 Plus, Dräger, Germany) to the closed-circuit automated rebreathing device (Detalo Performance^TM^, Detalo Health, Denmark).

The patient is instructed to normally breathe through the mouthpiece and to avoid respiration through the nose. At the beginning, the patient breathes 100% oxygen for 1 minute (flushing period), followed by application of a bolus of chemically pure carbon monoxide (CO, 99.997%, CO N47, Air Liquide, France) in a standardized way (weight- and gender-adapted predefined dose, CO-bolus application in two breath cycles) into the closed breathing circuit. The resulting gas mixture is rebreathed for 10 minutes (rebreathing period) under continuous monitoring of SpO_2_. To avoid CO leakage at the patient side, the air CO levels at the level of the mouthpiece and nose clip are monitored during the rebreathing period using a portable gas analyzer (Monoxor^®^ Plus, Bacharach, USA). When the rebreathing period is completed, the patient is instructed to fully exhale into the closed rebreathing circuitry before being disconnected from the rebreathing device. The CO, which was not up taken by the patient and thus remained in the closed rebreathing circuitry is measured via the same portable gas analyzer and subsequently being used to correct the CO-dependent calculations. Blood pressure and SpO_2_ at the end of the procedure are documented and another venous blood gas sample is drawn 30 seconds after the end of the test, i.e., 10 minutes after the administration of the CO bolus into the rebreathing circuit to allow sufficient blood mixing within patient’s vascular system following established recommendations ^(22)^. The blood gas analysis is performed with a hemoximeter (ABL825 Flex, Radiometer, Switzerland).

**Fig 3:**
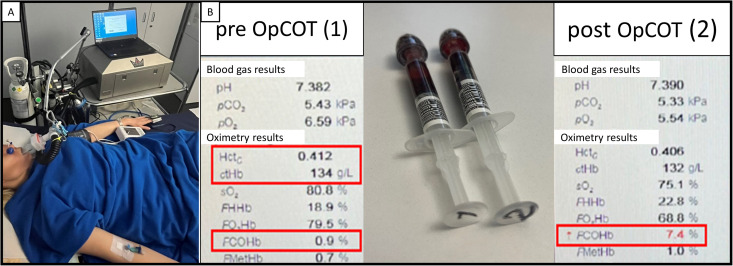
Blood volume profile measurement via optimized carbon monoxide rebreathing technique (OpCOT). Panel A: OpCOT installation. The patient is connected via mouthpiece with breathing filter to closed-circuit rebreathing device. Nose clipper and venous catheter have been installed. Blood pressure and oxygen saturation by pulse oximeter are being monitored during the procedure. Panel B: Venous blood gas analysis. Probe 1 has been drawn before OpCOT, probe 2 has been drawn 30 seconds after the end of the rebreathing period. Hematocrit (Hct), hemoglobin (Hb) and carboxyhemoglobin (COHb) before OpCOT and COHb after OpCOT are used to measure the total hemoglobin mass and estimate the blood volume profile, consisting of blood volume, plasma volume and red blood cell volume, as described in the method’s session.


Taking into consideration the pre-test measurements of Hct and Hb, the difference in COHb before and after OpCOT, as well as the volume of CO absorbed during the procedure, the Hb_mass_ is calculated (Fig 3). Then, RBCV, PV and BV can be determined as following:

RBCV [L= Hbmass [g] x Hct / Hb [g/L]

BV [L= RBCV [L] / Hct

PV [L= BV [L] – RBCV [L]

#### 2.3.3. Bioelectrical impedance analysis (BIA).

The body composition, fluid volumes and volume distribution are measured non-invasively via BIA technique using the InBody S10 device (InBody, Japan Inc., Tokyo, Japan) with 8 adhesive electrodes, applied on upper and lower extremities (2 on each extremity), according to the manufacturer’s instructions. The measurement is performed in supine position after a 10-minute resting period.

#### 2.3.4. Retinal vessel analysis (RVA).

Microvascular endothelial function is assessed by static and dynamic RVA using established protocols [24]. The RVA is evaluated by an Imedos dynamic retinal vessel analyzer (Imedos, Jena, Germany) with a Zeiss FF450 plus fundus camera (Carl Zeiss Meditec AG, Jena, Germany) connected with two charge-coupled device cameras (Imedos, Jena, Germany) for image acquisition and a customized computer software system for image analysis (Imedos, Jena, Germany).

After exclusion of serious ophthalmological diseases or history of epilepsy, 2 drops of 0.5% tropicamide are applied in one randomly selected eye to induce mydriasis. After a waiting time of at least 15 minutes to achieve sufficient mydriasis, the patient is positioned comfortably in front of the fundus camera and the non-examined eye is covered with an eye patch. First, we perform the dynamic RVA. After focusing on retina, the upper temporal fundus is visualized. Then, a 1.0–1.5 mm long segment of an artery and a vein are selected using the RVA software. In dynamic RVA the flicker-light response of 3 consecutive 20s-flicker-periods is measured and the flicker-light induced dilatation of the selected retinal artery and vein are evaluated.

Thereafter, the eye patch is removed and the static RVA is performed by taking fundus pictures of the retina in order to measure the diameters of the retinal arterioles (central retinal artery equivalent) and venules (central retinal vein equivalent) in standardized concentric segments and to finally calculate the arterio-venule-ratio.

#### 2.3.5. Flow-mediated vasodilatation (FMD).

Evaluation of the macrovascular function is performed via FMD of the brachial artery using an established protocol [25,26]. It is assessed by a high-resolution ultrasound vessel wall-tracking device with a 10 MHz linear array transducer (Siemens Acuson X300, Siemens AG, Germany).

Briefly, with this method the ability of endothelium to release nitric monoxide in response to shear stress after inducing temporary forearm ischemia, leading to vasodilatation, is evaluated.

FMD is performed in supine position. The brachial artery of the upper arm is visualized longitudinally with the ultrasound probe supported by a mechanical arm to assure stability, giving care to achieve a sharp and continuous near and distal wall. The arterial diameter is measured continuously for 10 minutes using a dedicated FMD software (FMD-Studio, Pisa, Italy) [27,28]. After a one-minute baseline measurement, a blood pressure cuff is inflated 50 mmHg above systolic pressure at the lower arm for 5 minutes. Then, the blood pressure cuff is released and hyperemia occurs. The FMD outcome is plotted as the percent increase in diameter from the baseline period mean diameter to the maximum diameter after cuff release.

Endothelial independent effect is also measured using pharmacological dilatation of the brachial artery after glycerol trinitrate (GTN). One sublingual hub of GTN spray is given (0.4 mg sublingual dose, nitrolingual spray, Pohl-Boskamp, Germany) and the brachial artery diameter is recorded for 6 minutes after drug administration.

GTN administration is avoided by decision of the investigator, if clinical contraindications, such as hypotension, are present.

#### 2.3.6. Arterial stiffness.

Arterial stiffness is assessed non-invasively by planar tonometry technique to evaluate pulse wave velocity (PWV) and pulse wave analysis (PWA) by use of the SphygmoCor® system (AtCor Medical, Germany) [29]. The PWA is evaluated at the level of the radial artery. The relevant outcome parameter is augmentation index normalized to a heart rate of 75 beats per minute. The PWV is measured between the carotid and femoral artery according to the recent guidelines for assessment of arterial compliance [30,31].

#### 2.3.7. Health-related quality of life and sleep in heart failure.

Quality of life and quality of sleep are assessed via standardized Kansas City Cardiomyopathy Questionnaires (KCCQ) [32] and Pittsburgh Sleep Quality Index (PSQI) questionnaires [33] respectively, which are completed during the baseline visit and the last follow-up visit.

#### 2.3.8. Transthoracic echocardiogram.

A complete transthoracic echocardiogram, including 3D-LVEF measurement, atrial and ventricular strain analyses, is performed according to current guidelines [34,35] in the echocardiography lab of University Heart Center Zurich.

#### 2.3.9. Cardiopulmonary exercise testing (CPET).

CPET is performed at the facilities of the outpatient clinic of cardiology in University Heart Center Zurich under supervision of a study physician, according to standardized protocols [36].

#### 2.3.9. Assessment of safety outcomes.

Adverse events and serious adverse events, including rehospitalizations due to decompensated HF, are routinely assessed by standardized questions, review of medical records and laboratory findings, and are recorded during every study visit. No serious adverse events necessitated early study treatment discontinuation.

### 2.4. Study outcomes

The primary study outcome is the mean change in PVS from baseline to 12 weeks of dapagliflozin treatment in comparison to placebo. The PVS is defined as the percentage of deviation of actual PV (aPV) from the sex-dependent ideal PV (iPV) as calculated by use of the following formula: *PVS = [(aPV – iPV)/ iPV)] x 100%*.

The aPV can be either calculated using the Hakim formula [37] or measured, e.g., via OpCOT. In our study, aPV is measured via OpCOT, as described above (aPV = PV). The iPV is calculated with the following sex-dependent simple formula [38]:

iPV* *=* *c x body weight [kg], constant c = 39 in males and c = 40 in females

The secondary outcomes include differences between the two study groups regarding blood volume profile, body water distribution, flicker-light induced retinal arteriolar and venular dilatation, PWV, FMD and GTN, as well as changes in various biomarkers and HF-related quality of life. For patients included in the advanced study program, differences in peak oxygen uptake during CPET and in echocardiographic parameters (LVEF, strain analyses, pulmonary artery systolic pressure) will be also assessed. Safety outcomes will be reported, including adverse and serious adverse events, changes in renal function, incidence of hypotension, urogenital infections and diabetic ketoacidosis.

### 2.5. Sample size estimation

A sample size estimation was performed for the primary study outcome (change in PVS) using R (V3.6.1) and PASS (V20.0.1). For the primary outcome a mixed model for repeated measures will be applied.

By using the data obtained from the work of Dekkers et al. [39] on dapagliflozin on estimated PV, a power calculation for a 2-level hierarchical, longitudinal mixed model design (2 group means up to the end of follow-up) [40] was performed and revealed a power > 0.8 for a sample size of 80 patients for the primary outcome. Accounting for about 10% dropouts, inclusion of 90 patients was aimed.

## 3. Data collection and management

All patients who either entered the study or were considered not eligible or were eligible, but not enrolled into the study, are documented on a screening log.

For data and query management, monitoring, reporting and coding, an internet-based secure data base secuTrial®, developed in agreement to Good Clinical Practice-guidelines and provided by the Clinical Trial Center Zurich, is used for this study. Data are collected using a specific electronic case report form (eCRF) with appropriate coded identification for each patient. Essential documents will be retained for at least 10 years after the regular end or a premature termination of the study. Any patient files and source data will be archived for the longest possible period (10 years).

## 4. Data analysis

Data will be exported from the secuTrial® electronic database for statistical analysis.

Statistical analyses will be performed by the investigators with support from a biostatistician using R statistics software V4.3.2 (R foundation of statistical computing). The dataset will comprise all patients initially randomized (intention-to-treat).

All continuous outcome data will be evaluated for normal distribution by graphical evaluation using histograms and Q-Q-plots. In case of strong deviations from normality, the continuous outcomes will be either logarithmically transformed to approximate normality before parametric modelling will be applied, or an alternative parametric model will be chosen. A p-value < 0.05 is considered to indicate statistical significance for the primary hypothesis. All other p-values will be considered exploratory.

### 4.1. Primary analysis

For the primary analysis, a linear mixed effects model for repeated measures will be applied for continuous outcome variables (e.g., change in PVS) to quantify the treatment effect between groups (dapagliflozin vs. placebo). Time will be coded as factor variable, and the model will include random intercepts for patients. The baseline value of the respective outcome will additionally be included as fixed effect. For testing of potential associations between outcome variables, Pearson’s correlation coefficient (r) will be used or in case of deviations from normality, Spearman’s rank correlation coefficient (ρ) will be used. A p-value < 0.05 is considered to indicate statistical significance.

### 4.2. Secondary analyses

As the secondary analysis, considering the changes of outcome variables from baseline over time, again a linear mixed effects model with the same specification as for primary analysis (treatment as fixed effect, time coded as factor variable, patient as random effect, baseline value as fixed effect) will be used to evaluate the interaction between time and treatment.

### 4.3. Interim analyses

No interim analysis of the primary outcome has been planned. However, if the descriptive evaluation of defined safety outcomes indicates higher incidences of adverse reactions than expected, an independent biostatistician, who will not be involved in the trial, might perform an unplanned interim analysis. At the time of manuscript’s submission, such an analysis was not required.

### 4.4. Safety analysis

Descriptive safety analyses will be performed based on defined safety outcome variables. However, formal statistical inference analysis will be performed.

### 4.5. Missing data and dropout

Study dropouts will be listed, showing reasons for discontinuation at study flow chart. All patients originally randomized will be included in the intention-to-treat analysis [41]. Additional patients will be recruited to replace potential dropouts in order to maintain sufficient statistical power for the primary outcome analysis. To handle missing data, 10-fold multiple imputation will be performed for the analysis of primary and secondary outcomes.

## 5. Discussion

To our knowledge, this study is the first double-blind, randomized, placebo-controlled trial evaluating the effects of dapagliflozin on blood volume profile and vascular function in HF patients after an ADHF event. Addressing the current lack of evidence, our study will expand the existing knowledge on mechanisms, on how SGLT-2i lead to the already proven favorable effects in HF patients irrespective of LVEF [10,11].

Adequate volume management in order to reach and sustain euvolemia and to avoid residual congestion is the main clinical challenge in management of HF patients, not only after an ADHF event, but also in the chronic stable state.

Considering the main study objective of the present trial, various favorable effects of SGLT-2i on important target organs of body fluid volume regulation including heart, kidney, vasculature and interstitial tissue were reported in literature recently. These effects may have the potential to improve body fluid volume balance and blood volume profile and furthermore support decongestion therapy in HF patients. At the intravascular level, SGLT-2i may alter blood volume constitution leading to a decrease in PV and eventually an increase in RBCV in a multi-factorial and complex manner and shift the PVS towards ‘ideal’ (normal) levels of healthy individuals as hypothesized in the present study (Fig 3). Ultimately, these beneficial effects on body fluid volume balance may improve tissue oxygen availability and subsequently functional capacity in HF patients.

Overall, the “volume-regulation-based” hypothesis is among a variety of other proposed mechanisms being responsible for the evident clinical benefits of SGTLT-2i in heart failure, which are currently extensively and critically discussed in several reviews [42–44].

Assessing the effects of dapagliflozin on blood volume profile and, especially, on plasma volume will provide valuable mechanistic insights into the role of SGLT-2 inhibition in heart failure. Martens et al. [18] reported that a higher estimated PVS was independently associated with a higher risk for HF hospitalization and all-cause mortality in a large cohort of HF patients from the entire LVEF-range. Kobayashi et al. [45] found that the estimated PVS at hospital discharge was independently associated with post-discharge clinical outcomes, such as re-hospitalization, worsening of HF or all-cause mortality, in three cohorts of patients admitted for ADHF. Data from our group [46], showed that stable patients with HF with preserved ejection fraction (n = 20, 25% females, mean age 71.5 ± 7.3 years, mean LVEF 55.7 ± 4.7%) and hypovolemia, have reduced Hb_mass_ and an overactivation of hormonal cascades regulating erythropoiesis and fluid homeostasis compared to healthy age- and sex-matched controls.

If our hypothesis that dapagliflozin in addition to GDMT leads to a more stable, euvolemic clinical state by improving estimated PVS compared to placebo from baseline to 12 weeks of treatment will be confirmed, a “volume-regulation-based” explanation for the beneficial effects of SGLT-2i in HF may be further supported. This may help to a better patient characterization and may lead to a more suitable, personalized volume management in HF patients by establishing the measurement of a novel clinical marker, such as PVS using OpCOT, in daily clinical practice. OpCOT is easily applicable and safe, rendering the blood volume profile estimation feasible in both inpatient and outpatient settings.

Furthermore, the trial seeks to provide novel randomized data about the effects of dapagliflozin on micro- and macro-vascular function. We showed that patients with compensated chronic HF (mean age 63.5 ± 11.2 years, 32% female, mean LVEF 37 ± 12.8%) have impaired retinal microvascular function [24]. If and to which extent dapagliflozin is able to improve the vascular function in these patients remains unclear. Our trial intends on revealing for the first time the underlying association between dapagliflozin and micro- and macro-vascular function outcomes.

In conclusion, the current trial will add further mechanistic information about the effects of SGLT-2 inhibition on intra- and extravascular volumes, as well as on micro- and macro-vascular function in HF patients. The results of our study will be discussed in detail in the final manuscript, which will be published in a peer-reviewed medical journal.

## Supporting information

S1 File Original study protocol. Study protocol as reviewed and accepted by ethics authorities.
(PDF)

S2 File SPIRIT checklist. (PDF)

## Abbreviations

aPV: Actual plasma volume
iPV: Ideal plasma volume
ADHF: Acute decompensated heart failure
BIA: Bioelectrical impedance analysis
BV: Blood volume
CO: Carbon monoxide
COHb: Carboxyhemoglobin
CPET: Cardiopulmonary exercise testing
eCRF: Electronic case report form
eGFR: Estimated glomerular filtration rate
FMD: Flow-mediated vasodilation
GTN: Glycerol trinitrate
GDMT: Guideline-directed medical therapy
Hb: Hemoglobin
Hb_mass_: Total hemoglobin mass
Hct: Hematocrit
HF: Heart failure
IRT: Interactive response technology
KCCQ: Kansas City Cardiomyopathy Questionnaire
LVEF: Left ventricular ejection fraction
OpCOT: Optimized carbon monoxide rebreathing technique
PQSI: Pittsburgh Sleep Quality Index
PWA: Pulse wave analysis
PWV: Pulse wave velocity
PV: Plasma volume
PVS: Plasma volume status
RBCV: Red blood cell volume
RVA: Retinal vessel analysis
SGLT-2i: Sodium glucose cotransporter-2 inhibitors
SpO_2_: Oxygen saturation by pulse oximeter
