# Supplementary material for: Effects of dapagliflozin on blood volume status and vascular outcomes in clinically stabilized heart failure patients after an acute decompensated heart failure event (DAPA-VOLVO study): Protocol of a double-blind randomized controlled clinical trial
Source: PLoS One. 2025 Jul 2;20(7):e0325668. doi: 10.1371/journal.pone.0325668 (PMC12221041; doi:10.1371/journal.pone.0325668)
Supplement: S1 File — Study protocol as reviewed and accepted by ethics authorities. (PDF) [file pone.0325668.s001.pdf]

Clinical Study Protocol

Effects of Dapagliflozin on blood volume status and vascular function in clinically compensated heart failure patients after an acute heart failure event.

A Double-Blind Randomized Controlled Phase IV Clinical Trial

DAPAGLIFLOZIN ON **VOL**UME **V**ASCULAR **O**UTCOMES, **DAPA-VOLVO**

Study Type:	Clinical trial with Investigational Medicinal Product (IMP)
Study Categorisation:	Category C
Study Registration:	Clinicaltrials.gov<br>NCT ID: NCT04869124
Study Identifier:	DAPA-VOLVO
Sponsor, Sponsor-Investigator or Principal Investigator:	Prof. Dr. med. Frank Ruschitzka<br>University Heart Center Zurich<br>Cardiology<br>Rämistrasse 100<br>CH-8091 Zurich<br>[illegible]<br>[illegible]<br>[illegible]
Investigational Product:	Dapagliflozin propanediol (FORXIGA™)
Protocol Version and Date:	
	**4.0, 30.04.2023**

[illegible]

Signature Page(s)

Study number	BASEC-Nr.: 2020-01920
Study Title	Effects of Dapagliflozin on blood volume status and vascular function in clinically compensated heart failure patients after an acute heart failure event. A Double-Blind Randomized Controlled Phase IV Clinical Trial<br>DAPAGLIFLOZIN ON VOLUME VASCULAR OUTCOMES, DAPA-VOLVO

The Sponsor-Investigator and trial statistician have approved the protocol version 4.0 (dated 30.04.2023), and confirm hereby to conduct the study according to the protocol, current version of the World Medical Association Declaration of Helsinki, ICH-GCP guidelines or ISO 14155 norm if applicable and the local legally applicable requirements.

Sponsor-Investigator: Prof. Dr. med. Frank Ruschitzka

Zürich,	
Place/Date	Signature

Table of Contents

STUDY SYNOPSIS **6**
ABBREVIATIONS **13**
STUDY SCHEDULE **19**
1. STUDY ADMINISTRATIVE STRUCTURE **22**
1.1 Sponsor, Sponsor-Investigator 22
1.2 Principal Investigator(s) 22
1.3 Statistician ("Biostatistician") 23
1.4 Laboratory 23
1.5 Monitoring institution 23
1.6 Data Safety Monitoring Committee 23
1.7 Investigators 23
1.8 Any other relevant Committee, Person, Organisation, Institution 25
2. ETHICAL AND REGULATORY ASPECTS **27**
2.1 Study registration 27
2.2 Categorisation of study 27
2.3 Competent Ethics Committee (CEC) 27
2.4 Competent Authorities (CA) 27
2.5 Ethical Conduct of the Study 27
2.6 Declaration of interest 28
2.7 Patient Information and Informed Consent 28
2.8 Participant privacy and confidentiality 28
2.9 Early termination of the study 28
2.10 Protocol amendments 28
3. BACKGROUND AND RATIONALE **30**
3.1 Background and rationale in heart failure 30
3.1.1 Acute decompensated heart failure events and congestion in heart failure patients 30
3.1.2 Implications of relative plasma volume status in heart failure 33
3.1.3 SGLT-2is and cardioprotection in heart failure- a ‘volume-based’ perspective 35
3.2 Investigational product and indication 39
3.2.1 Drug description and treatment indication 39
3.2.2 Pharmacokinetics and drug metabolism in humans 40
3.3 Preclinical evidence 40
3.4 Clinical evidence 41
3.4.1 Dapagliflozin on primary composite outcome of DAPA-HF trial 41
3.4.2 Additional findings of DAPA-HF sub-analyses and DEFINE-HF outcomes 43
3.5 Dose rationale: Rationale for the intended purpose in study 43
3.6 Explanation for choice of placebo 43
3.7 Risks / benefits 43
3.8 Justification of choice of study population 44
4. STUDY OBJECTIVES **45**
4.1 Overall Objective 45
4.2 Primary Objective 45
4.3 Secondary Objectives 45
4.4 Safety Objectives 45

5. STUDY OUTCOMES 46
5.1 Primary Outcome 46
5.2 Secondary Outcomes 46
5.3 Other Outcomes of Interest 46
5.4 Safety Outcomes 47
6. STUDY DESIGN 48
6.1 General study design and justification of design 48
6.1.1 General study design 48
6.1.2 Justification of study design 49
6.2 Methods of minimising bias 49
6.2.1 Randomisation 49
6.2.2 Blinding procedures 50
6.2.3 Other methods of minimising bias 50
6.3 Unblinding procedures (code break) 50
7. STUDY POPULATION 51
7.1 Eligibility criteria 51
7.1.1 Inclusion criteria 51
7.1.2 Exclusion criteria 51
7.2 Recruitment and screening 52
7.3 Assignment to study groups 52
7.4 Criteria for withdrawal / discontinuation of participants 52
8. STUDY INTERVENTION 53
8.1 Identity of investigational products (treatment) 53
8.1.1 Experimental intervention (treatment) 53
8.1.2 Control intervention (placebo treatment) 53
8.1.3 Packaging, labelling and supply (re-supply) 53
8.1.4 Storage Conditions 54
8.2 Administration of experimental and control interventions 54
8.2.1 Experimental and control intervention (placebo) 54
8.3 Dose modifications 54
8.4 Compliance with study intervention 55
8.5 Data collection and follow-up for withdrawn participants 55
8.6 Trial specific preventive measures 55
8.7 Concomitant interventions (treatments) 56
8.8 Study drug accountability 56
8.9 Return or destruction of study drug 56
9. STUDY ASSESSMENTS 56
9.1 Study flow chart and table of study procedures and assessments 56
9.2 Assessments of outcomes 56
9.2.1 Assessment of primary outcome 56
9.2.2 Assessment of secondary outcomes 59
9.2.3 Assessment of other outcomes of interest 61
9.2.4 Assessment of safety outcomes 64
9.2.5 Assessments in participants who prematurely stop the study 64
9.3 Procedures at each visit 64
9.3.1 Visit 1 (V1, Screening visit) 64

9.3.2 Visit 2/5 (V2/V5, Baseline visit/Final visit) ... 65
9.3.3 Visit 2a/5a ... 65
9.3.4 Visit 3/4 (V3/4, Follow-up visit 1/2) ... 66
9.3.5 Visit 6 (V6, Follow-up visit of prospective observational study) ... 66
10. SAFETY ... 67
10.1 Drug studies ... 67
10.1.1 Definition and assessment of (serious) adverse events and other safety related events ... 67
10.1.2 Reporting of serious adverse events (SAE) and other safety related events ... 68
10.1.3 Follow up of Serious Adverse Events ... 69
11. STATISTICAL METHODS ... 69
11.1 Hypothesis ... 69
11.2 Determination of Sample Size ... 70
11.3 Statistical criteria of termination of trial ... 70
11.4 Planned Analyses ... 70
11.4.1 Datasets to be analysed, analysis populations ... 70
11.4.2 Primary Analysis ... 71
11.4.3 Secondary Analyses ... 71
11.4.4 Interim analyses ... 71
11.4.5 Safety analysis ... 71
11.4.6 Deviation(s) from the original statistical plan ... 71
11.5 Handling of missing data and drop-outs ... 71
12. QUALITY ASSURANCE AND CONTROL ... 72
12.1 Data handling and record keeping / archiving ... 72
12.1.1 Case Report Forms ... 72
12.1.2 Specification of source documents ... 73
12.1.3 Record keeping / archiving ... 73
12.2 Data management ... 73
12.2.1 Data Management System ... 73
12.2.2 Data security, access and back-up ... 74
12.2.3 Analysis and archiving ... 74
12.2.4 Electronic and central data validation ... 74
12.3 Monitoring ... 74
12.4 Audits and Inspections ... 75
12.5 Confidentiality, Data Protection ... 75
12.6 Storage of biological material and related health data ... 76
13. PUBLICATION AND DISSEMINATION POLICY ... 77
14. FUNDING AND SUPPORT ... 77
14.1 Funding ... 77
14.2 Other Support ... 77
15. INSURANCE ... 77
16. REFERENCES ... 77
17. LIST OF TABLES AND FIGURES ... 83
17.1 List of tables ... 83
17.2 List of figures ... 83
18. APPENDICES ... 83

STUDY SYNOPSIS

**Sponsor / Sponsor-Investigator**	Prof. Dr. med. Frank Ruschitzka
**Study Title:**	Effects of Dapagliflozin on blood volume status and vascular function in clinically compensated heart failure patients after an acute heart failure event.<br>A Double-Blind Randomized Placebo-Controlled Phase IV Clinical Trial
**Short Title / Study ID:**	DAPAGLIFLOZIN ON VOLUME VASCULAR OUTCOMES,<br>DAPA-VOLVO
**Protocol Version and Date:**	4.0, 30.04.2023
**Trial registration:**	Clinicaltrials.gov: NCT04869124
**Study category and Rationale**	Clinical trial with IMP category C
**Clinical Phase:**	4
**Background and Rationale:**	Patients with heart failure (HF) have a high overall mortality and poor prognosis. Fluid volume overload and congestion, frequently combined with anemia [1] are central pathophysiological issues in HF patients leading to increased hospitalization rates and mortality [2]. Functional disturbances of the cardio-renal-axis are supposed to play a key role in this process [3]. The sodium-glucose cotransporter 2 inhibitors (SGLT-2is) were shown in major trials to be cardio- and reno-protective with additional trials ongoing to further address this topic [4]. Most importantly, the recently published results of the DAPA-HF trial demonstrated that treatment with the SGLT-2i Dapagliflozin reduced the risk of worsening heart failure or death from cardiovascular causes in patients with HF with reduced ejection fraction irrespective of diabetes [5]. The beneficial effects of SGLT-2is on blood volume balance and erythropoiesis and subsequent amelioration of systemic hemodynamics, vascular function and oxygen transport all resulting in improved tissue oxygen supply have emerged as a mechanistic hypothesis to explain the cardio-reno-protective effects of Dapaglifozin in particular [6]. Thus, treatment with SGLT-2is might be particularly beneficial in clinically compensated HF patients after hospitalization due to an acute decompensated heart failure (ADHF) event. These HF patients frequently present with persistent blood volume imbalances (e.g. plasma volume overload) and thus an higher risk of worsening heart failure and re-hospitalization [7], especially during the 'vulnerable' phase within the first 1-3 months after clinical discharge [8].
**Objective(s):**	The primary study objective is whether Dapagliflozin on top of recommended therapy improves relative plasma volume status (PVS) in clinically stabilized heart failure patients after an acute decompensated heart failure (ADHF) event. The main secondary study objectives are whether Dapagliflozin also improves macro- and micro-vascular function in these patients.

**Outcome(s):**	Primary study outcome:<br>• Mean change in relative plasma volume status (ΔPVS) from baseline to 12 weeks of dapagliflozin treatment in comparison to placebo;<br>Secondary study outcomes:<br>• Blood volume profile (Optimized CO-rebreathing, OpCO) ¤;<br>• Body water content and distribution (Bioimpedance analysis, BIA) ¤;<br>• Retinal vessel function (Retinal vessel analysis, RVA) §;<br>• Arterial stiffness (Pulse wave analysis, PWA) §;<br>• Endothelial function (Flow-mediated dilation, FMD) §;<br>• HF-hospitalization (either or: worsening of heart failure, WHF*) °;<br>• HF-related quality of life (Kansas City cardiomyopathy questionnaire, KCCQ) °;<br>• Percentage of patients being within the optimal PVS divergence interval for HF ($\%PVS_{opt}$) ¤;<br>Tertiary study outcomes:<br>• Exercise capacity (CPET: VO_{2peak}, $VEVCO_{2slope}$, 6MWD) °;<br>• Left ventricular morphology and function (TTE: LVM, LVEF) °;<br>• Sympathetic activity (MSNA) °;<br>• Tissue sodium content (Na-MRI: skin, muscle, cartilage) ¤<br>• Renal perfusion and renal oxygen consumption (ASL ^{1}H-MRI and BOLD MRI);<br>• Total amount of albumin and other blood constituents (OpCO) ¤;<br>• Cardio-renal biomarkers (e.g. FGF23) °;<br>• Iron metabolic biomarkers (serum ferritin, TSAT, hepcidin) °;<br>Safety outcomes (main study: V2-V5):<br>• AEs/SAEs;<br>• Renal function (eGFR);<br>• Incidence/events of symptomatic hypotension (systolic BP< 90 mmHg in 3 consecutive measurements);<br>• Incidence/events of genital (GI-) and urinary tract infections (UTI);<br>• Incidence/ events of back pain;<br>• Incidence/events of diabetic ketoacidosis (DKA);<br>• Incidence/events of Fournier gangrene (FG);<br>• Laboratory biomarker of heart failure (NT-proBNP);<br>• Other relevant laboratory parameter;<br><br>**¤** Primary volume-related outcomes, **§** primary vascular function-related outcomes, **°** other outcomes;<br><br>* WHF: Requiring intensification of therapy in the ambulatory or hospital setting including i.v. diuretics, i.v. nitrates, or other medications for HF, or institution of mechanical or ventilator support.<br># $\%PVS_{opt}$: Percent of patients within proposed optimal PVS divergence interval for HF as defined by the combined divergence interval with the lowest reported hazard ratio (H_R) for HF-hospitalization and mortality: -4% to -18% of iPV [9, 10]);
**Study design:**	Randomized, placebo controlled, parallel group design;

**Inclusion / Exclusion criteria:**	Inclusion criteria:<br>1. Male or female, age of 18 or older;<br>2. Patients with documented diagnosis of chronic or de novo heart failure (NYHA II-IV) and clinically stabilized (considered for hospital discharge) after hospitalization/ ambulatory care because of an acute decompensated (congestive) heart failure (ADHF) event;<br>3. eGFR ≥ 30 mL/min/1.73 m^2 (CKD-EPI formula) at enrolment;<br>4. Provision of signed informed consent prior to any study specific procedure;<br>Exclusion criteria:<br>1. Contraindications to the class of drugs under study, e.g. known hypersensitivity or allergy to class of drugs or the investigational product;<br>2. Receiving therapy with an SGLT2 inhibitor within 8 weeks prior to enrolment or previous intolerance of an SGLT2 inhibitor;<br>3. Participation in another study with investigational drug within the 30 days preceding and during the present study;<br>4. Type 1 diabetes mellitus;<br>5. Symptomatic hypotension or systolic blood pressure <90 mmHg at 2 out of 3 measurements either at visit 1 or visit 2;<br>6. Coronary revascularization (percutaneous coronary intervention because of STEMI or coronary artery bypass grafting) or valvular repair/replacement within 12 weeks prior to enrolment or planned to undergo any of these operations after randomization;<br>7. Implantation of a CRT device within 12 weeks prior to enrolment or intent to implant a CRT device during 12 weeks of study observation period if indicated according to the actual guidelines [11];<br>8. Previous cardiac transplantation or implantation of a ventricular assistance device or similar device, or implantation expected after randomization;<br>9. HF due to restrictive cardiomyopathy, active myocarditis, constrictive pericarditis, hypertrophic obstructive cardiomyopathy or uncorrected primary valvular disease;<br>10. Symptomatic bradycardia or second or third degree heart block without a pacemaker;<br>11. Severe (eGFR <20 mL/min/1.73 m^2 by CKD-EPI), unstable or rapidly progressing renal disease;<br>12. Women who are pregnant or breast feeding;<br>13. Intention to become pregnant during the course of the study;<br>14. Known or suspected non-compliance, drug or alcohol abuse;<br>15. Inability to follow the procedures of the study, e.g. due to language problems, psychological disorders, dementia, etc. of the participant;<br>16. Patients with severely restricted liver function;<br>17. Patients with recurrent mycotic genital infections;

**Measurements and procedures:**	• Blood volume (BV) and BV-related parameters (OpCO);<br>• Body water content and distribution (BIA);<br>• Macro-vascular function (PWA, FMD);<br>• Micro-vascular function (RVA);<br>• Sympathetic activity (MSNA);<br>• Cardiac function (TTE);<br>• Exercise performance (CPET);<br>• Tissue sodium content (Na-MRI);<br>• Renal perfusion and renal oxygen consumption (ASL ^{1}H-MRI, BOLD-MRI);<br>• Clinical outcome measures (WHF and re-hospitalization rate);<br>• Health-related quality of life for HF (KCCQ);<br>• Venous blood and urine sample analysis (e.g. cardio-renal biomarkers);
**Study Product / Intervention:**	Dapagliflozin propanediol (FORXIGA™): 10 mg once daily p.o. on top of recommended standard therapy;<br>Duration of drug administration: 12 weeks;
**Control Intervention (if applicable):**	Placebo: Once daily p.o. on top of recommended standard therapy;<br>Duration of product administration: 12 weeks;
**Number of Participants with Rationale:**	80 patients (40 patients per group, 2-treatment arms);<br>Rationale: A priori power analysis for primary outcome (see 11.2);
**Study Duration:**	2 years (optimal recruitment estimation) to 4 years (conservative recruitment estimation)
**Study Schedule:**	Month Year of First-Participant-In (planned): 04/2021<br>Month Year of Last-Participant-Out (planned): 12/2024 (conservative recruitment estimation)

Investigator(s):	
**Investigator(s):**	Name: Frank Ruschitzka, MD<br>Address: Rämistrasse 100, 8091 Zürich<br><br>Name: Andreas Flammer, MD<br>Address: Rämistrasse 100, 8091 Zürich<br><br>Name: Isabella Sudano, MD, PhD<br>Address: Rämistrasse 100, 8091 Zürich<br><br>Name: Thomas Haider, MD<br>Address: Rämistrasse 100, 8091 Zürich<br><br>Name: Jens Barthelmes, MD<br>Address: Rämistrasse 100, 8091 Zürich<br><br>Matthias Nägele, MD<br>Address: Rämistrasse 100, 8091 Zürich<br><br>Name: Leonie Kreysing, MD<br>Address: Rämistrasse 100, 8091 Zürich<br><br>Name: Delia Nebunu, MD<br>Address: Rämistrasse 100, 8091 Zürich

	Name: Mattia Arrigo, MD<br>Address: Birmensdorferstrasse 497,<br>8063 Zürich<br><br>Name: David Niederseer, MD, PhD<br>Address: Rämistrasse 100, 8091 Zürich<br><br>Name: Ulrike Held, PhD<br>Address: Hirschgraben 84, 8001 Zürich<br><br>Name: Cristina Rossi, PhD<br>Address: Rämistrasse 100, 8091 Zürich<br><br>Name: Natallia Laptseva, MD<br>Address: Rämistrasse 100, 8091 Zürich<br><br>Name: Kostantinos Bitos<br>Address: Rämistrasse 100, 8091 Zürich<br><br>Name: Elena Osto, MD, PhD<br>Address: Wagistrasse 14, 8952 Schlieren

	Name: Carsten Wagner, MD<br>Address: Winterthurerstrasse 190, 8057 Zürich
**Study Centre(s):**	Single-centre
**Statistical Considerations:**	Sample size calculation for primary study outcome (ΔPVS);<br>• Normal-distribution check: Shapiro-Wilk test, histograms, QQ-plots;<br>• Homogeneity of variance check: Levene's test;<br>• Primary analysis: Mixed model for repeated measures (MMRM) for primary endpoint analysis;<br>Association analysis: Pearson's correlation coefficient or Spearman's ranked correlation coefficient;<br>• Secondary analysis: MMRM to evaluate treatment effect at different time points along the observation period or ANCOVA for outcomes, which are only measured at baseline (V2) and at the end of treatment period (V5);<br>• Significance level: $p < 0.05$;
**GCP Statement:**	**This study will be conducted in compliance with the protocol, the current version of the Declaration of Helsinki, the ICH-GCP as well as all national legal and regulatory requirements.**

ABBREVIATIONS

^{125}I	Radioisotope of iodine 125
^{14}C	Radiocarbon
6MWD	6-min walk distance
6MWT	6-min walk test
ADHF	Acute decompensated heart failure
AE	Adverse event
AHF	Acute heart failure
Aix@75	Augmentation index normalized to a heart rate of 75 beats per minute
ALT	Alanine transaminase
AP	Alcalic phosphatase
aPV	Actual plasma volume
AS	Arterial stiffness
ASL	Arterial spin labelling
ASR	Annual safety report
AUC	Area under the curve
AVR	Arterio-venule-ratio
AZ	AstraZeneca
BASEC	Business administration system for ethical committees
BIA	Bioimpedance analysis
BNP	Brain natriuretic peptide
BOLD	Blood oxygenation level dependent
BP	Blood pressure
BSA	Body surface area
BUN	Blood urea nitrogen
BV	Blood volume
BV_{ratios}	Blood volume ratios
CA	Competent authority (e.g. Swissmedic)
Ca^{2+}	Calcium
CABG	Coronary artery bypass graft surgery
CAD	Coronary artery disease
CCD	Charge-coupled device
CEC	Cantonal ethics committee
cFGF23	C-terminal fragment of fibroblast growth factor 23
CHF	Chronic heart failure
CKD	Chronic kidney disease
CKD-EPI	Chronic kidney disease-epidemiology collaboration

ClinO	Ordinance on clinical trials in human research<br>*(in German: KlinV, in French: OClin, in Italian: OSRUm)*
C_{max}	Maximal serum concentration
COB	Carbon monoxide bolus
CPET	Cardiopulmonary exercise testing
CPT	Cold pressor test
CRA	Cardio-renal axis
CRAE	Central retinal artery equivalent
CRF	Case report form
CRP	C-reactive protein
CRT	Cardiac resynchronization therapy
CRVE	Central retinal vein equivalent
CTCAE	Common terminology criteria for adverse events
CT-proAVP	C-terminal pro arginine vasopressin
CV	Coefficient of variation
CYP	Cytochrome P450
d	Effect size
d	Days
DAPA	Dapagliflozin
DAPA-CKD	Dapagliflozin in chronic kidney disease
DAPA-HF	Dapagliflozin in heart failure
DKA	Diabetic ketoacidosis
DSUR	Development safety update report
DVA	Dynamic vessel analysis
E2	Estradiol
ECG	Electrocardiography
eCRF	Electronic case report form
ECW	Extra-cellular water
EF	Ejection fraction
eGFR	Estimated glomerular filtration rate
EPO	Erythropoietin
ePV	Estimated plasma volume
ESR	Externally sponsored research
FG	Fournier gangrene
FGF23	Fibroblast growth factor 23
FIDa	Flicker-light induced retinal artery dilatation
FIDv	Flicker-light induced retinal vein dilatation
FMD	Flow mediated dilatation
FMS	Finapress medical systems
FP	Flushing phase

FPFV	First patient first visit
FSR	First study results
fT3	Free triiodothyronine
fT4	Free tetraiodthyronine
GCP	Good clinical practice
GFR	Glomerular filtration rate
GI	Genital infection
GTN	Glyceryl trinitrate
H_0	Null hypothesis
H_1	Alternative hypothesis
Hb	Hemoglobin concentration
HbCO	Carboxyhemoglobin concentration
Hb_{mass}	Total hemoglobin mass
Hct	Hematocrit
HFmrEF	Heart failure with mid-range ejection fraction
HFpEF	Heart failure with preserved ejection fraction
HFrEF	Heart failure with reduced ejection fraction
H_R	Hazard ratio
HR	Heart rate
HRA	Federal act on research involving human beings
HRQoL	Health-related quality of life
hsTnT	High-sensitive troponin T
i.v.	Intravenous injection
IB	Investigator’s brochure
ICW	Intra-cellular water
IF	Interstitial fluid
iFGF23	Intact form of fibroblast growth factor 23
IHD	Ischemic heart disease
IIT	Investigator-initiated trial
IL-6	Interleukine 6
iPV	Ideal plasma volume
IRT	Interactive response technique
ISO	International organisation for standardisation
ITT	Intention to treat
K	Kelvin
K^+	Potassium
KCCQ	Kansas City cardiomyopathy questionnaire
LA	Lactate
lme	Linear mixed effect model
LPFV	Last patient first visit

LPLV	Last patient last visit
LVEF	Left ventricular ejection fraction
LVM	Left ventricular mass
LVMI	Left ventricular mass indexed (BSA)
m	Months
MCHC	Mean corpuscular haemoglobin concentration
MCV	Mean corpuscular volume
MD	Medical Device
MedDO	Medical device ordinance
MMRM	Mixed model for repeated measures
MPO	Myeloperoxidase
mPV	Measured plasma volume
MSNA	Muscle sympathetic nerve activity
N.A.	Not applicable
Na^+	Sodium
Na-MRI	Sodium (^{23}Na) magnetic resonance imaging
$nCO_{absorbed}$	Amount of absorbed carbon monoxide
nHb_{tagged}	Amount of tagged hemoglobin
nHb_{total}	Total amount of hemoglobin
NIBPD	Non-invasive blood pressure device
NS	Not significant
NT-proBNP	N-terminal-pro hormone brain natriuretic peptide
NYHA	New York heart association
OpCO	Optimized carbon monoxide rebreathing
P_{atm}	Atmospheric pressure
PI	Principal investigator
PK	Pharmacokinetic
PLA	Placebo
PR	Patient recruitment
PS	Patient security
PSO	Primary study outcome
PTH	Parathyroide hormone
PV	Plasma volume
PVS	Plasma volume status
PWA	Pulse wave analysis
PWV	Pulse wave velocity
R	Gas constant
r	Pearson’s correlation coefficient
RAAS	Renin angiotensin aldosterone system
RBCM	Red blood cell mass

RBCV	Red blood cell volume
RBP	Rebreathing phase
RP	Resting phase
RVA	Retinal vessel analysis
SABP	Systolic arterial blood pressure
SafeSO	Safety study outcomes
SaO_2	Peripheral oxygen saturation
SD	Standard deviation
SDV	Source data verification
SGLT2-i(s)	Sodium glucose co-transporter 2 inhibitor(s)
SI	Systemic inflammation
SOP(s)	Standard operating procedure(s)
SPC	Summary of product characteristics
SSO	Secondary study outcomes
STEMI	ST-elevation myocardial infarction
SUSAR	Suspected unexpected serious adverse reaction
SVA	Static vessel analysis
T	Room temperature in Kelvin
t1/2	Half life time
T2DM	Type II diabetes mellitus
TBW	Total body water
TE	Typical error
TMAO	Trimethylaminoxide
t_{max}	Time take to reach C_{max}
TMF	Trial master file
TMS	Treatment start
TSAT	Transferrin saturation
TSH	Thyoroid-stimulationg hormone
TSO	Tertiary study outcomes
TTE	Transthoracic echocardiography
UF	Ultra filtration
USZ	University Hospital Zurich
UTI	Urinary tract infection
Val-HeFT	Valsartan in heart failure trial
$VCO_{absorbed}$	Absorbed volume of carbon monoxide
VEGF	Vascular endothelial growth factor
$VEVCO_{2slope}$	Ventilation carbon dioxide excretion slope
VO_{2max}	Maximal oxygen uptake
VO_{2peak}	Peak oxygen uptake
VOLVO	Volume vascular outcomes

w	Weeks
WHF	Worsening heart failure
Y_0	Variable Y mean at baseline
Y_1	Variable Y mean after a certain treatment period
ΔHbCO	Difference in carboxyhemoglobin concentration
ΔPVS	Change in plasma volume status
ρ	Spearman's rank correlation coefficient

STUDY SCHEDULE

DAPA-VOLVO Flow Chart:

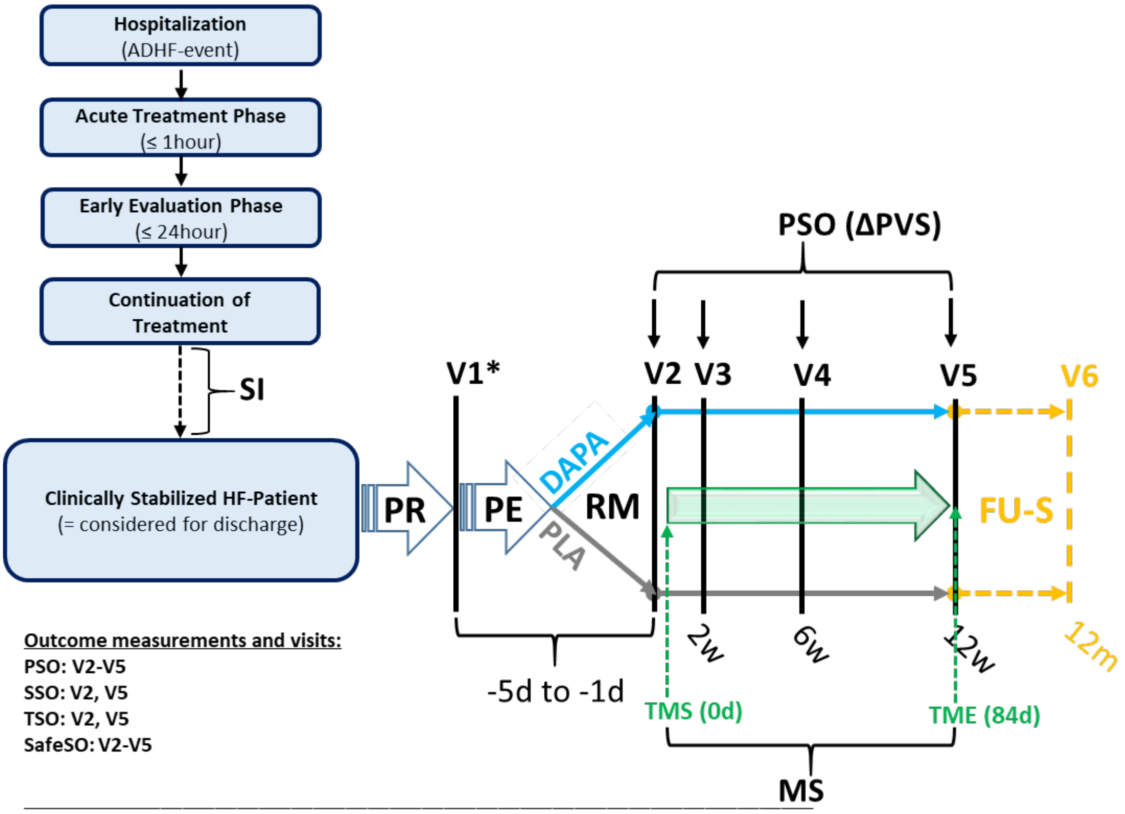

SI: Provision of study information in oral and written form ≥ 24h prior to V1;
V1: 1st Visit: Screening visit (-5d to -1d): Patient enrolment, randomization;
V2: 2nd Visit: Baseline visit (-2d to -1d): PSO (SSO, TSO, SafeSO) measurement at baseline (PSO ≤ 48h prior to treatment start = 0d);
V3: 3rd Visit (2w): PSO follow-up measurement 2 weeks post treatment start;
V4: 4th Visit (6w): PSO follow-up measurement 6 weeks post treatment start;
V5: 5th Visit: (12w): PSO follow-up measurement 12 weeks post treatment start;
V6: 6th Visit: Follow-up visit: Prospective observational outcome evaluation 12 months post treatment end (V5);
* V1 will be integrated into V2 whenever logistically feasible/possible
PSO: Primary study outcome, **SSO:** Secondary study outcomes, **TSO:** Tertiary study outcomes, **SafeSO:** Safety Outcomes, **ΔPSV:** Mean change in PVS from baseline to 12 weeks of treatment, **PR:** Patient recruitment, **PE:** Patient enrolment, **RM:** Randomization, **DAPA:** Dapagliflozin treatment (10mg/day), **PLA:** Placebo, **TMS:** Treatment start (0d), **TME:** Treatment end (84d), **MS:** Main study, **FU-S:** Follow-up study, **d:** days, **w:** weeks, m: months;

Fig.1: DAPA-VOLVO study flow chart. After being hospitalized/ or admitted for ambulatory care due to an ADHF event and an initial clinically treatment phase, the HF-patient will be contacted and the study information (SI) will be provided in oral and written form at least 24 hours prior to visit 1. The clinically stabilized (considered for discharge) and interested HF-patient will be recruited (PR) for the screening visit (V1) and if criteria are fulfilled enrolled (PE) and randomly (RM) assigned to either dapagliflozin treatment arm (DAPA) or placebo arm (PLA). Note: V1 will be integrated into V2 whenever logistically feasible/possible. The baseline assessment of study outcomes, especially of the primary study outcome (PSO) should be performed within 48 hours prior to treatment start (TMS, 0d) or it should be documented 'why' baseline assessment/ treatment start had to be delayed. Following IMP treatment start follow-up visits will be performed after 2 weeks (V3), 6 weeks (V4) and 12 weeks (V5) of treatment. The optional visit 12 months post IMP treatment end (V6) is part of the prospective observational follow-up study (FU-S) and will be evaluated and analyzed independently from the main study (V2-V5).

Table 1. Overview on study visits and interventions along the study period.

Study Periods (0d= treatment start)	Screening	Baseline [#, *]/Follow-up				Follow-up study (optional)[§]
Visit	1	2	3	4	5	6
Time [o]: days (d), weeks(w), months (m);	-5 to -1d	-2 to -1d	2w ± 5d	6w ± 5d	12w ± 5d	12m ± 2w
Patient Information and Informed Consent	x[1]					
Demographics	x					
Medical History	x[2]	x[3]	x[3]	x[3]	x[3]	x[3]
In- /Exclusion Criteria	x	x	x	x	x	
Physical Examination	x	x	x	x	x	x
Vital Signs	x	x	x	x	x	x
Laboratory Tests	x[4]	x[6]	x[5]	x[5]	x[6]	x[5]
Pregnancy Test	x[7]					
Randomization		x				
Primary study outcome (PVS)		x	x	x	x	x
Administer Study Medication		x[8]	x	x	x	
Primary Variables		x	x	x	x	x
Secondary Variables		x			x	
Tertiary Variables		x			x	
Safety Variables		x	x	x	x	
Recommended standard therapy	x	x	x	x	x	x
Adverse Events		x	x	x	x	

[1] Study information (SI) will be provided to the study candidate ≥ 24 hours prior to screening visit (V1);

[2] Includes complete cardiac history, NYHA-graded symptoms, assessment of cardiovascular risk factors (smoking, alcohol, drugs), current medications, allergies, clinical examination;

[3] Includes reported cardiovascular complaints and symptoms, NYHA-graded symptoms, current medication, clinical examination focused on symptoms of heart failure and congestion, body weight;

[4] Screening laboratory examinations: Red blood cell count, platelet count, leukocyte count, hemoglobin, hematocrit, eGFR (creatinine), sodium, potassium, GPT, alkaline phosphatase, NT-proBNP;

[5] Safety laboratory examinations plus cardio-renal biomarkers: eGFR, creatinine, NT-proBNP, FGF23;

[6] Complete laboratory examinations (see 9.2.3);

[7] Premenopausal females or females in menopause ≤ 2 year;

[8] The treatment will start in every study patient after the baseline testing is completed;

[#] The baseline measurement of primary study outcome should be performed ≤ 48h prior to treatment start (0d);

[*] The treatment should start prior to hospital discharge whenever feasible;

[§] The prospective observational follow-up study visit 12 months after IMP treatment stop (Visit 6) will be evaluated and analysed independently from the main study (Visit 2 to Visit 5);

[o] The time frame is referred to the IMP treatment start (0d) except from V6, which is referred to the IMP treatment end (84d). If for a particular reason the enrolment of the hospitalized/ admitted for ambulatory care patient and/or

the IMP treatment start has to be delayed to the post discharge phase the reason should be documented as note to file and the relative study time frame maintained.

1. STUDY ADMINISTRATIVE STRUCTURE

1.1 Sponsor, Sponsor-Investigator

Prof. Dr. med. Frank Ruschitzka
University Heart Center Zurich
Cardiology
Rämistrasse 100
CH-8091 Zurich

1.2 Principal Investigator(s)

Prof. Dr. med. Frank Ruschitzka (Sponsor/PI)
University Heart Center Zurich
Cardiology
Rämistrasse 100
CH-8091 Zurich

Andreas Flammer, MD (Co-PI)
University Heart Center Zurich
Rämistrasse 100, 8091 Zürich

Isabella Sudano, MD, PhD (Co-PI)
University Heart Center Zurich
Rämistrasse 100, 8091 Zürich

Dr. med. univ. Thomas Haider (Co-PI)
University Heart Center Zurich
Cardiology
Rämistrasse 100

CH-8091 Zurich

1.3 Statistician ("Biostatistician")

Prof. Dr. Ulrike Held, PhD
Epidemiology, Biostatistics and Preventive Institute (EBPI)
Address: Hirschgraben 84CH-8001 Zurich

1.4 Laboratory

Name: Prof. Dr. med. Arnold von Eckardstein
Address: University Hospital Zurich, Rämistrasse 100, 8091 Zürich

1.5 Monitoring institution

Clinical Trials Center (CTC)
Tasks: Study monitoring
UniversitätsSpital Zürich
Rämistrasse 100 / MOU2
8091 Zürich

1.6 Data Safety Monitoring Committee

N.A.

1.7 Investigators

Name: Frank Ruschitzka, MD
Address: Rämistrasse 100, 8091 Zürich

Name: Andreas Flammer, MD

Address: Rämistrasse 100, 8091 Zürich

Name: Isabella Sudano, MD, PhD
Address: Rämistrasse 100, 8091 Zürich

Name: Elena Osto, MD, PhD
Address: Wagistrasse 14, 8952 Schlieren

Name: Thomas Haider, MD
Address: Rämistrasse 100, 8091 Zürich

Matthias Nägele, MD
Address: Rämistrasse 100, 8091 Zürich

Name: Leonie Kreysing, MD
Address: Rämistrasse 100, 8091 Zürich

Name: Delia Nebunu, MD
Address: Rämistrasse 100, 8091 Zürich

Name: Kostantinos Bitos
Address: Rämistrasse 100, 8091 Zürich
[illegible]

Name: Mattia Arrigo, MD
Address: Birmensdorferstrasse 497, 8063 Zürich

Name: David Niederseer, MD, PhD
Address: Rämistrasse 100, 8091 Zürich

Name: Cristina Rossi, PhD
Addresse: Rämistrasse 100, 8091 Zürich

Name: Carsten Wagner, MD
Addresse: Winterthurerstrasse 190, 8057 Zürich

1.8 Any other relevant Committee, Person, Organisation, Institution

Name: Michaela Gemperle
Tasks: Study coordinator
Address: Rämistrasse 100, 8091 Zürich

Name: KEK (Zürich)
Tasks: Competent ethics committee (CEC)
Address: Stampfenbachstrasse 121, 8090 Zürich

Name: Swissmedic
Tasks: Competent authority (CA)
Address: Hallerstrasse 7, 3012 Bern

2. ETHICAL AND REGULATORY ASPECTS

Before the study will be conducted, the protocol, the proposed patient information and consent form as well as other study-specific documents will be submitted to a properly constituted Competent Ethics Committee (CEC) and competent authorities (Swissmedic) in agreement with local legal requirements, for formal approval. Any amendment to the protocol must as well be approved (if legally required) by these institutions.

The decision of the CEC and Swissmedic competent authority concerning the conduct of the study will be made in writing to the Sponsor-Investigator before commencement of this study. The clinical study can only begin once approval from all required authorities has been received. Any additional requirements imposed by the authorities shall be implemented.

2.1 Study registration

The study will be registered in a registry (https://clinicaltrials.gov) listed in the WHO International Clinical Trials Registry Platform (ICTRP, http://www.who.int/ictrp/en/) [12]. In addition, registration in a national language in the Swiss National Clinical trial Portal (SNCTP via BASEC) is required.

2.2 Categorisation of study

This study is a category C study because the study product (Dapagliflozin) is authorized for medical use by Swissmedic in Switzerland, however the product colorant and engraving is different from the commercially available product. The study investigations only involve minimal risks for the patients.

2.3 Competent Ethics Committee (CEC)

The responsible investigator at each site ensures that approval from an appropriately constituted Competent Ethics Committee (CEC) is sought for the clinical study.

The reporting duties and allowed time frame (all changes in the research activity and all unanticipated problems involving risks to humans; including in case of planned or premature study end and the final report) are respected. No substantial changes/amendments are made to the protocol without prior Sponsor and CEC approval, except where necessary to eliminate apparent immediate hazards to study participants.

Premature study end or interruption of the study is reported within 15 days. The regular end of the study is reported to the CEC within 90 days, the final study report shall be submitted within one year after study end. Amendments are reported according to chapter 2.10.

2.4 Competent Authorities (CA)

The Sponsor will obtain approval from the competent authority (Swissmedic) before the start of the clinical trial following the legal instruction that CA approval is necessary for all studies category B and C (MD).

The reporting duties and allowed time frame to CA including the reporting duties in case of planned or premature study end and the final report are respected. Reporting duties and timelines are the same as for CEC (see 2.3), except of non-substantial amendments that shall be reported as soon as possible. Amendments are reported according to chapter 2.10.

2.5 Ethical Conduct of the Study

The study will be carried out in accordance to the protocol and with principles enunciated in the current version of the Declaration of Helsinki [13], the guidelines of Good Clinical Practice (GCP) issued by ICH [14, 15], the Swiss Law and Swiss regulatory authority's requirements. The CEC and regulatory authorities will receive annual safety and interim reports and be informed about study stop/end in agreement with local requirements.

2.6 Declaration of interest

The principal investigator declares no conflict of interest with the conduct of the study.

2.7 Patient Information and Informed Consent

The investigators will explain to each participant the nature of the study, its purpose, the procedures involved, the expected duration, the potential risks and benefits and any discomfort it may entail. Each participant will be informed that the participation in the study is voluntary and that he/she may withdraw from the study at any time and that withdrawal of consent will not affect his/her subsequent medical assistance and treatment.

The participant must be informed that his/her medical records may be examined by authorised individuals other than their treating physician.

All participants for the study will be provided a participant information sheet and a consent form describing the study and providing sufficient information for participant to make an informed decision about their participation in the study. If needed or requested by the potential study candidate, a time frame of at least 24 hours will be given to the study candidate to decide whether to participate or not (see also Fig. 1, Table 1).

The participant information sheet and the consent form will be submitted with the protocol for review and approval for the study by the CEC and by Swissmedic.

The formal consent of a participant, using the approved consent form, must be obtained before the participant is submitted to any study procedure.

The participant should read and consider the statement before signing and dating the informed consent form, and should be given a copy of the signed document. The consent form must also be signed and dated by the investigator (or his designee) at the same time as the participant sign, and it will be retained as part of the study records.

2.8 Participant privacy and confidentiality

The investigator affirms and upholds the principle of the participant's right to privacy and that they shall comply with applicable privacy laws. Especially, anonymity of the participants shall be guaranteed when presenting the data at scientific meetings or publishing them in scientific journals.

Individual subject medical information obtained as a result of this study is considered confidential and disclosure to third parties is prohibited. Subject confidentiality will be further ensured by utilising subject identification code numbers to correspond to treatment data in the computer files.

For data verification purposes, authorised representatives of the Sponsor (-Investigator), a competent authority (e.g. Swissmedic), or an ethics committee may require direct access to parts of the medical records relevant to the study, including participants' medical history.

2.9 Early termination of the study

The Sponsor-Investigator (and any competent authority) may terminate the study prematurely according to certain circumstances:

- ethical concerns,
- insufficient participant recruitment,
- when the safety of the participants is doubtful or at risk, respectively,
- alterations in accepted clinical practice that make the continuation of a clinical trial unwise,
- early evidence of benefit or harm of the experimental intervention;

2.10 Protocol amendments

The study investigators are allowed to amend the protocol or to provide suggestions for a protocol amendment in correspondence with the study PI. Important protocol modifications will be communicated by either by the study coordinator or the PI to the study staff.

Significant changes to be authorized by the CEC are the following:

- changes affecting the participants' safety and health, or their rights and obligations;
- changes to the protocol, and in particular changes based on new scientific knowledge which concern the trial design, the method of investigation, the endpoints or the form of statistical analysis;
- a change of trial site, or conducting the clinical trial at an additional site; or
- a change of sponsor, coordinating investigator or investigator responsible at a trial site.

Substantial amendments are only implemented after approval of the CEC and CA respectively.

Under emergency circumstances, deviations from the protocol to protect the rights, safety and well-being of human subjects may proceed without prior approval of the sponsor and the CEC/CA. Such deviations shall be documented and reported to the sponsor and the CEC/CA as soon as possible.

All non-substantial amendments are communicated to the CA as soon as possible if applicable and to the CEC within the Annual Safety Report (ASR).

3. BACKGROUND AND RATIONALE

3.1 Background and rationale in heart failure

3.1.1 Acute decompensated heart failure events and congestion in heart failure patients

3.1.1.1 The recurring cycle of decompensation in heart failure- an urgent need for improvement

Despite major advancements in the diagnosis, prevention and treatment of chronic heart failure (CHF), prognosis and quality of life in HF patients remain limited with an average life expectancy as short as in many types of neoplastic malignant diseases [16]. An important and crucial factor that substantially contributes to the poor prognosis in HF patients is the frequent occurrence of acute decompensated heart failure (ADHF) events resulting in hospitalization and intensified medical care in these patients [2].

The majority of decompensated HF patients are presented with certain degree of volume overload and congestion, clinically termed as 'wet' phenotype [17] and thus undergo volume-lowering treatment, which comprises different phases from acute to stable conditions with the ultimate goal to reach 'euvolemia' and clinical discharge [7, 18]. However, as stated previously the assessment of volume status is currently based primarily on clinical signs and indirect measures (e.g. changes in body weight) and thus remains a clinical challenge [18].

As a consequence persistent blood volume imbalances at hospital discharge are very common in HF patients after an ADHF event and are associated with a higher risk of worsening of heart failure (WHF) and re-hospitalization, especially within the following 1 to 3 months after clinical discharge- also known as the 'vulnerable' phase [7, 8].

The current lack of established methods to accurately and reliable assess the volume status in HF patients in clinics in combination with sub-optimal therapeutic interventions to reach and maintain 'euvolemia' in these patients results in a recurring cycle of decompensation (Fig. 2) [19] with its negative consequences on disease outcomes and health-related quality of life in HF-patients and its substantial contribution to the high socio-economic burden of heart failure globally [17].

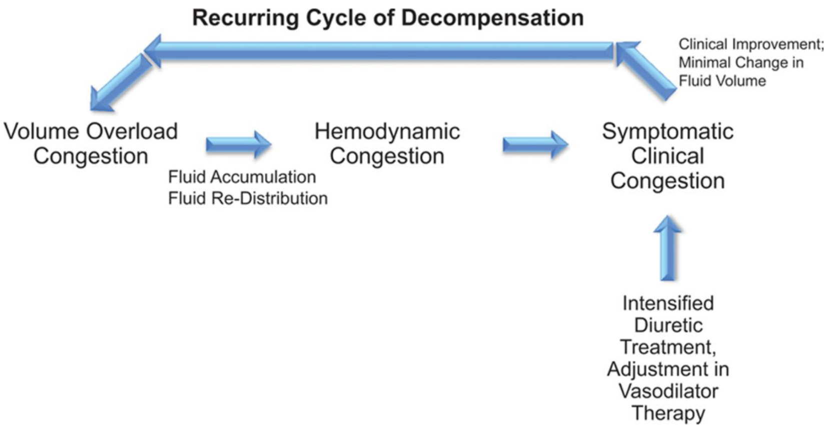

Fig. 2: Recurring cycle of decompensation in heart failure (adapted from Miller et al. [19]).

3.1.1.2 A short glimpse into the pathophysiology of volume overload and congestion in heart failure

The pathophysiology of volume overload and congestion in heart failure is complex and yet not fully understood. Multiple organs (e.g. kidney, liver, gut, bone marrow) and physiological control systems of body homeostasis including the immune system and neuroendocrine systems, e.g. the renin-angiotensin-aldosterone system (RAAS) are involved in this process [19]. The cardio-renal axis (CRA) and its functional disturbance, clinically named 'cardio-renal syndrome' [20], frequently leads to sodium and fluid retention within the patients body.

Thus, CRA dysfunction can be seen as the pathophysiological epicentre of volume overload and congestion in HF (Fig. 3).

Ultimately, the malfunction of the CRA typically results in a combined disturbance of body fluid balance and erythropoiesis with negative functional and structural consequences on the cardiovascular system. The process of congestion that often proceeds an ADHF event has a profound

time variability ranging from several days to weeks up to eventually years and is often bi-phasic, starting with a symptomatically ‘silent’ phase of slow volume expansion, partly driven by sodium and fluid retention, which is stored and therefore buffered as ‘cardio-inactive’ volume, primarily in the interstitial fluid compartment (Fig. 4) but also in the intravascular capacity sections (e.g. the splanchnic venous bed) [19, 21].

This often asymptomatic phase is then followed by a clinically symptomatic (e.g. dyspnoea and cough) phase of ‘active’ volume overload and cardio-pulmonary congestion [19]. The ‘phase-transition’ between the two symptom-based volume states can either occur solely over time with a predominant filling of the extravascular compartments until the capacity limit is exceeded and volume drains into the intravascular compartment or relatively fast via stress-induced sympathetic nerve activation and subsequent fluid redistribution of cardio-inactive fluid volume, e.g. from the ‘splanchnic venous reservoir’ [21].

Systemic inflammation (SI) as a pathophysiological response of the immune system also plays an important role in the process of volume imbalance and congestion [22]. As recently reviewed by Linthout et al. [23] SI can be a cause and/or consequence of congestion in heart failure. For example, venous congestion can directly induce SI and SI itself can substantially contribute to congestive heart failure [24].

On the other hand, SI can trigger hypoalbuminemia, endothelial cell activation and increased cytokine release (e.g. IL-6), which can lead to increased capillary leakage and subsequently to a disturbance of fluid-shifts between the intravascular and interstitial fluid compartments [22]. SI can also induce iron deficiency mainly mediated via inflammatory driven up-regulation of hepcidin with or without presence of anemia [25].

Indeed, iron deficiency and anemia are both common pathologic conditions in CHF patients and associated with reduced exercise capacity, impaired health-related quality of life and increased mortality risk [26, 27].

Another mechanism by which the immune system (both, innate and adaptive branch) interacts with the regulation of body fluid balance is the control of tissue sodium content, e.g. within the skin and muscles [28].

Recent pre-clinical data revealed that high-salt diet leads to hypertonic (in comparison to plasma) sodium accumulation in the skin, which stimulates macrophages to release vascular endothelial growth factor (VEGF)-C to bind to its receptor, which is expressed on skin lymphatic vessels and induces hyperplasia of the cutaneous lymph capillary network with profound consequences on interstitial fluid (IF) regulation [29].

Thus, the sodium retention observed in HF patients may lead to an increased interstitial sodium content impacting IF compartment and contributing to the process of volume overload and congestion in these patients.

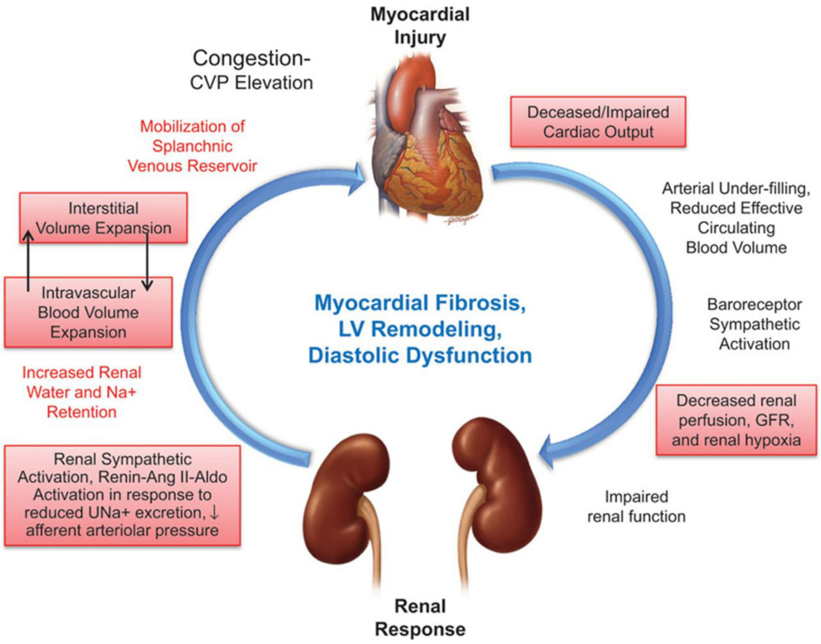

Fig.3 Cardio-renal interactions in volume expansion and congestion in chronic heart failure. GFR: glomerular filtration rate, LV: left ventricle (adapted from Miller et al. [19]).

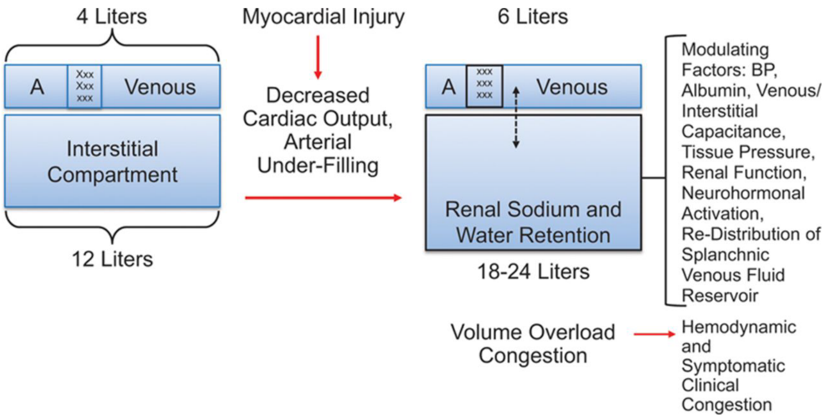

Fig. 4: Paradigm of interstitial and intravascular volume expansion in chronic heart failure. BP: blood pressure (adapted from Miller et al. [19]).

3.1.1.3 The heterogeneity of imbalanced volume profiles in heart failure

In HF patients a vast heterogeneity of volume profiles exists (Fig. 5) [19].

For example, hypervolemia can be the result of solely plasma volume expansion with normal red blood cell mass (RBCM) and can lead to hemodilution, which is reflected by the term 'dilutional' or 'pseudo'-anemia [30, 31].

In contrast, 'true' anemia is defined by a reduction in RBCM and volume (RBCV) [32, 33].

Here, Otto et al. [33] recently reported that Hb and total haemoglobin mass (Hb_{mass}) correlated well in healthy control subjects while this relationship was 'poor' in CHF patients.

However, hypervolemia in CHF patients can be also the result of both PV and RBCV expansion. Indeed, a recent study in compensated CHF patients with reduced left ventricular ejection fraction (HFrEF) concluded that an RBCV is a 'relevant' contributing factor to blood volume (BV) expansion.

Furthermore, we could recently show that hypovolemia can be also present in HF patients as shown in a cohort of HF patients with preserved ejection fraction (HFpEF), eventually as a result of overshooting volume lowering therapy [34].

Thus, the accurate evaluation of the volume profile in HF patients, e.g. by use of the CO-rebreathing technique (OpCO) may profoundly improve therapy and clinical outcomes in HF-patients.

Indeed, a recently published retrospective study analysis performed by Strobeck et al. [35] in a large cohort of acute heart failure (AHF) patients with mixed ejection fractions revealed that blood volume (BV)-guided management in these HF-patients reduced mortality and rehospitalization due to HF.

However, efficient therapeutic strategies are warranted to not just help to re-balance the volume status after an ADHF event but also to help to maintain and stabilize an euvolemic status in treated patients.

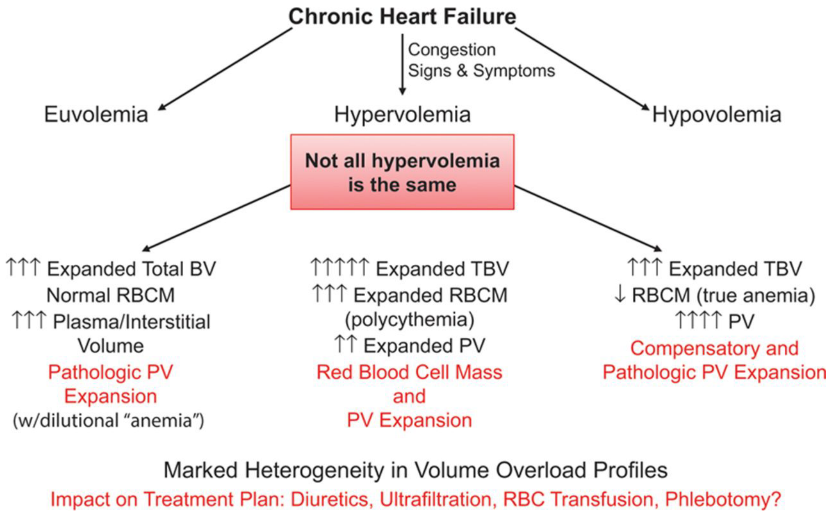

Fig. 5: Heterogeneity in hypervolemic profiles in heart failure. Quantitative blood volume (BV) analysis identifies multiple plasma volume (PV) and red blood mass (RBCM) profiles, which affects approach to treatment. UF: ultrafiltration (adapted from [19]).

3.1.2 Implications of relative plasma volume status in heart failure

3.1.2.1 Relative plasma volume status and clinical outcomes in heart failure

The relative plasma volume status (PVS) is a parameter that reflects the relative deviation of the actual plasma volume (aPV) from the calculated ideal plasma volume (iPV) of a person.

The aPV can be either estimated (ePV), e.g. by the 'Hakim' formula [36], which is based on the actual subjects hematocrit, body weight and sex-dependent constant factors or measured (mPV), e.g. by use of the optimized CO rebreathing (OpCO) technique. The iPV is calculated using a well-established formula based on body weight and on a sex-dependent constant factor.

The PVS has been recently shown to be predictive for clinical outcomes [9, 10, 37]. For example, Ling et al. showed that the PVS was associated with morbidity and mortality in a large cohort (more than 5000 patients of the Valsartan in Heart Failure Trial, Val-HeFT) of HF patients with reduce ejection fraction (HFrEF) [9].

Furthermore, Grodin et al. [37] found that the PVS was independently associated with long-term clinical outcomes (e.g. all-cause mortality and HF hospitalization) in a large cohort (more than 3000 patients) of HF patients with preserved ejection fraction (HFpEF).

Martens et al. [10] reported that a higher PVS was independently associated with a higher risk for HF hospitalization and all-cause mortality in a large cohort of HF patients with mixed (HFrEF, HFmrEF, HFpEF) ejection fractions. Interestingly, the hazard ratio (H_R) plot for HF hospitalization and all-cause mortality against PVS showed a J-shape curve (Fig. 6) with a prominent incline in H_R starting at a PVS value of +2% percent and at > -20% on the negative scale in relation to iPV. The graphical illustration also reveals that above a PVS of +2% every additional 2% increase is associated with an increase in the event H_R. The same authors also reported that the calculated 'optimal' PVS defined as PVS with lowest event H_R is a slightly contracted (-6.5%) volume status in comparison to the calculated iPV (Fig. 6).

Additionally, Kobayashi et al. found that the estimated PVS at hospital discharge was independently associated with post-discharge clinical outcomes (e.g. re-hospitalization, worsening of HF or all-cause mortality) in 3 cohorts of patients admitted for ADHF. The estimated PVS in this study was calculated by use of the 'Strauss formula', which is based on actual hematocrit levels and hemoglobin concentrations of the patients.

Also, in patients undergoing coronary artery bypass graft surgery (CABG) a higher PVS was associated with worse inpatient outcomes after CABG [38].

In summary, the PVS clearly seems to have important prognostic implications in chronic HF patients with mixed ejection fractions and also in hospitalized acute decompensated (congestive) heart failure

(ADHF) patients. Thus, therapies that improve the PVS towards 'euvolemia' (PVS= 0, or slightly below) by shifting the actual plasma volume (aPV) closer to ideal plasma volume (iPV) may improve clinical outcomes in HF patients in general but in ADHF patients in particular.

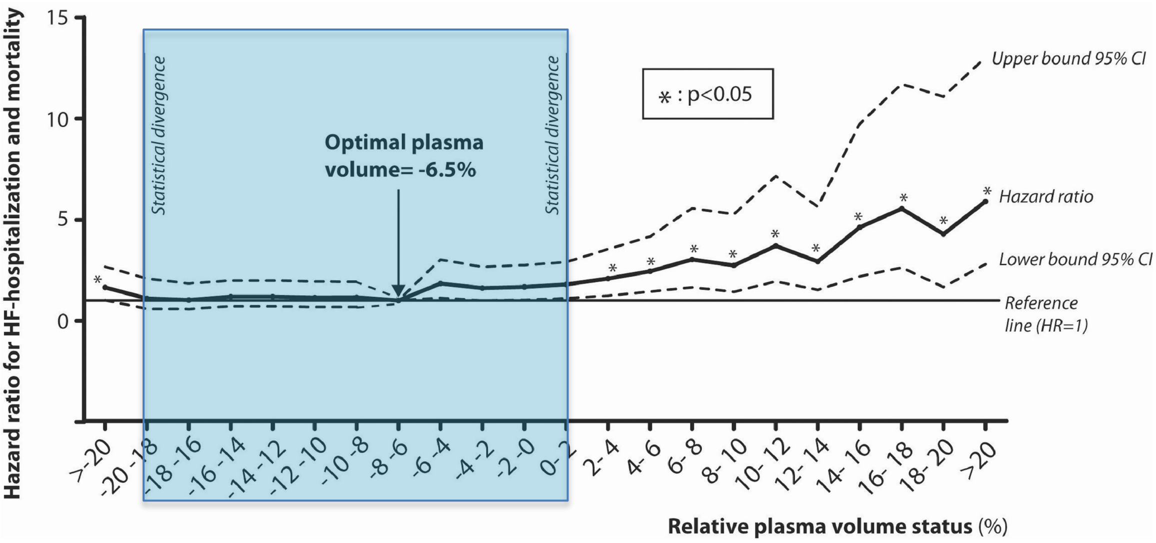

Fig. 6: Relationship between plasma volume status and risk of heart failure hospitalization and all-cause mortality is J-shaped. The blue square indicates the optimal plasma volume divergence interval without an increase in hazard ratio (H_R) for HF-hospitalization and mortality (adapted and modified from Martens et al. [10]).

3.1.2.2 Relative plasma volume status measured with the optimized CO-rebreathing technique

The plasma volume (PV) will be measured by use of the OpCO technique to calculate the PVS as the primary outcome variable. Although the OpCO technique is not yet established in clinical routine it is a highly accurate and reliable method as reflected by a reported low typical error (TE, 1-2%) and coefficient of variation (CV, 2.2%) for measuring total haemoglobin mass (Hb_{mass}), the basis to calculate intravascular volumes [39-41].

The safety and feasibility of the OpCO technique in HF patients, even in those with a severely reduced left ventricular function as indicated by a very low (< 40%) LVEF have been recently proven [42].

Furthermore, the recent applications of the OpCO technique to measure blood- and plasma volumes in patients with chronic kidney disease [43], dialysis-dependent kidney failure [44, 45] and in patients with chronic liver disease [46] further emphasizes the feasibility of applying OpCO technique in the clinical setting and also shows the brought ranged potential of this technique in the clinics.

Additional validation studies that compare the OpCO technique with other gold standard methods are currently ongoing (internal communication with Detalo Healths company, unpublished data). A direct comparison between the estimated PV (ePV) assessed by the Hakim formula [36] and the measured PV (mPV) by OpCO technique to calculate the PVS has not yet been performed.

However, a recent study performed by Martens et al. [10] could demonstrate that the measured PV in HF patients by use of a radioactive isotope (technetium-99) red blood cell (RBC) labelling technique correlated well with the estimated PV as calculated by the Hakim formula.

In this context, Gore et al. [39] could further show in a meta-analysis that the measurement error of CO-rebreathing is comparable (even lower) to radioactive RBC labelling technique. Thus, we think that the measured PV by use of OpCO technique will reflect well the estimated PV based on the 'Hakim' formula.

Actually, we think it may reflect 'true' PV levels in HF patients more accurately than with the 'Hakim' formula as it's based on the direct measurement of volume independent Hb_{mass} (for further details see also 9.2.1) and not solely on concentration dependent measures (e.g. Hct).

Nevertheless, we will compare (correlate) the PVS obtained from the 2 different approaches with each other. Importantly, the OpCO technique is in contrast to radioactive isotope labelling techniques relatively easy to apply and fast (results within 15 minutes), and does not expose patients to radiation.

3.1.3 SGLT-2is and cardioprotection in heart failure- a 'volume-based' perspective

3.1.3.1 SGLT-2is- from diabetes to heart failure with and without kidney disease

Originally designed to treat diabetes, the sodium-glucose-co-transporter 2 inhibitors (SGLT-2is), which are gathered under the term 'gliflozins', were proven in several large controlled trials to be cardio- and also reno-protective [47].

For example, the study results of the EMPA-REG OUTCOME trial with more than 7000 enrolled patients revealed that the SGLT-2i empagliflozin substantially reduced the risk (in comparison with placebo) of major adverse cardiovascular events, cardiovascular (-38%) and all-cause death (-32%) as well as hospitalization for heart failure (-38%) in patients with type 2 diabetes mellitus and atherosclerotic cardiovascular disease [48, 49].

Most importantly, the DAPA-HF trial with 4744 enrolled patients was the first study to show that the SGLT-2i dapagliflozin lowered the risk of worsening of heart failure or death from cardiovascular causes (primary composite study outcome) in HFrEF patients independent from the presence or absence of diabetes [5, 50] (for more details see also 3.4.1).

The currently ongoing trials (overview about finished and ongoing outcome trials are summarized by Lytvyn et al. [51] and Patel et al. [6]) will further reveal whether the cardio-protective effect of SGLT-2is is also present in HF-patients with preserved ejection fraction (HFpEF) [6].

Apart from the heart, the SGLT-2is were also shown to be reno-protective [52]. In this context, it has been recently announced by AstraZeneca that the ongoing DAPA-CKD trial [53] will be stopped early due to an 'overwhelming' efficiency [54].

Mechanistically, SGLT-2is were designed to primarily target the co-transporter for sodium and glucose in the renal proximal tubule and subsequently reduce their reabsorption in the kidney.

However, the potential mechanisms that mediate the cardio-reno-protective effects of SGLT-2is in HF patients are pleiotropic (Fig. 7) and currently intensively investigated and also debated as summarized in several recent reviews on this topic [4, 6, 51, 55-59].

3.1.3.2 SGLT-2i-mediated cardioprotection in heart failure- a 'volume-based' hypothesis

Among the various proposed mechanistic hypothesis about the cardio-protective effect of SGLT-2is, the beneficial impact on volume regulation and body fluid balance has especially come into scientific focus. Indeed, a retrospective analysis of the EMPA-REG OUTCOME trial found that changes in markers related to plasma volume (e.g. Hct, Hb, albumin) were the most important mediators of risk reduction for cardiovascular deaths after the treatment with SGLT-2i in comparison with placebo [60].

As illustrated in Fig. 7 (blue squares) the treatment with SGLT-2is modulates fluid volume regulation in multiple ways. For example, the SGLT-2i dapagliflozin was shown to exert both natriuretic and osmotic diuretic effects in patients with type 2 diabetes and kidney damage [61]. Furthermore, it was proposed by use of mathematical modelling of data on plasma-/urinary sodium and water that dapagliflozin reduces the interstitial fluid volume relative to intravascular volume to greater extent than classical loop-diuretics, e.g. bumetanide [62].

Additional evidence comes from another recently published study in patients with diabetic kidney disease (DKD) [63]. The study results showed by use of bio-impedance analysis (BIA) that dapagliflozin treatment reduced intracellular (ICW)-, extracellular (ECW)- and total body water (TBW) in these patients after 1 week of treatment.

Importantly, the study authors found that only in patients with dapagliflozin treatment (in contrast to furosemide treatment) the baseline ECW/TBW correlated with the observed changes in ECW/TBW ($r = -0.590$, $p < 0.001$) and ECW ($r = -0.374$, $p = 0.025$) after 1 week of treatment. The study authors concluded that the effect magnitude of fluid volume reduction induced by dapagliflozin is dependent on the a priori 'volume-status' of the patient, suggesting that the drug effect is eventually more pronounced in patients with severe extracellular fluid retention than in patients with less, which further indicates a 'volume-stabilizing' effect of the drug.

Of note, Ohara et al. [64] reported in another work published previously that dapagliflozin treatment affected the fluid volume distribution in CKD patients differently than other classical diuretics such as furosemide, which further emphasizes the unique effects of SGLT-2is on the 'volume-status'.

Considering the above findings, the treatment with SGLT-2is might be in particular beneficial in HF

patients after being hospitalized due to an ADHF event as these patients are frequently present with ‘subclinical’ volume overload and congestion, especially within the interstitial fluid compartment, which potentially contributes to their high re-hospitalization rate after clinical discharge [7].

As also illustrated in Fig. 7, SGLT-2 inhibition has a dual effect on blood volume that is primarily mediated via the kidney. SGLT-2is reduce plasma volume and concomitant stimulate erythropoiesis [65, 66], which results in an increased oxygen transport capacity. Additionally, the SGLT-2is can lower albuminuria in HF patients [67], which leads to a reduced loss of the osmotic active proteins via the kidney and may help to maintain normal blood albumin levels and subsequently stabilize the volume distribution between the intravascular and interstitial fluid compartments.

Hypoalbuminemia is very common in HF patients and is associated with an increased risk of death [68, 69]. Here, a recent multivariate analysis showed that the serum albumin levels among other factors, e.g. blood pressure and hemoglobin concentration were independent predictors of long-term mortality in acute heart failure patients [70].

Another important volume stabilizing effect of SGLT-2is is their anti-inflammatory potential. As stated previously (see section 3.1.1.2), systemic inflammation also contributes to volume dysregulation and congestion in heart failure.

In this context, the inner layer of every vessel, named the endothelium and its function may play a central role. Endothelial dysfunction is a ‘hallmark’ of heart failure condition and associated with increased mortality [71, 72]. The endothelium not only controls the vessel wall diameter with its paracrine and endocrine secretory activity but also its integrity and permeability, which is crucial for fluid exchange between the intravascular and interstitial fluid compartment [73].

The anti-inflammatory effect of SGLT-2is in combination with the lowering effect on blood pressure protects the endothelium and its function, which may translate to an improved volume balance and distribution. Indeed, several studies found evidence for a beneficial effect of SGLT-2is on endothelial function [74].

In terms of relative plasma volume status (PVS), a recent a pooled data analysis in 4533 patients with type 2 diabetes showed that dapagliflozin decreased the estimated PV (ePV) by 9.6% compared to placebo after 12 months of treatment [75].

In the same study, the investigators could also show that the calculated ePV by use of the ‘Strauss’ formula correlated well with the actually measured PV (mPV) using the ^{125}I-human serum albumin labelling method. The findings suggest that treatment with SGLT-2is can improve PVS, however it needs to be shown whether these findings can be translated to clinically stabilized HF patients after being hospitalized due to an ADHF event.

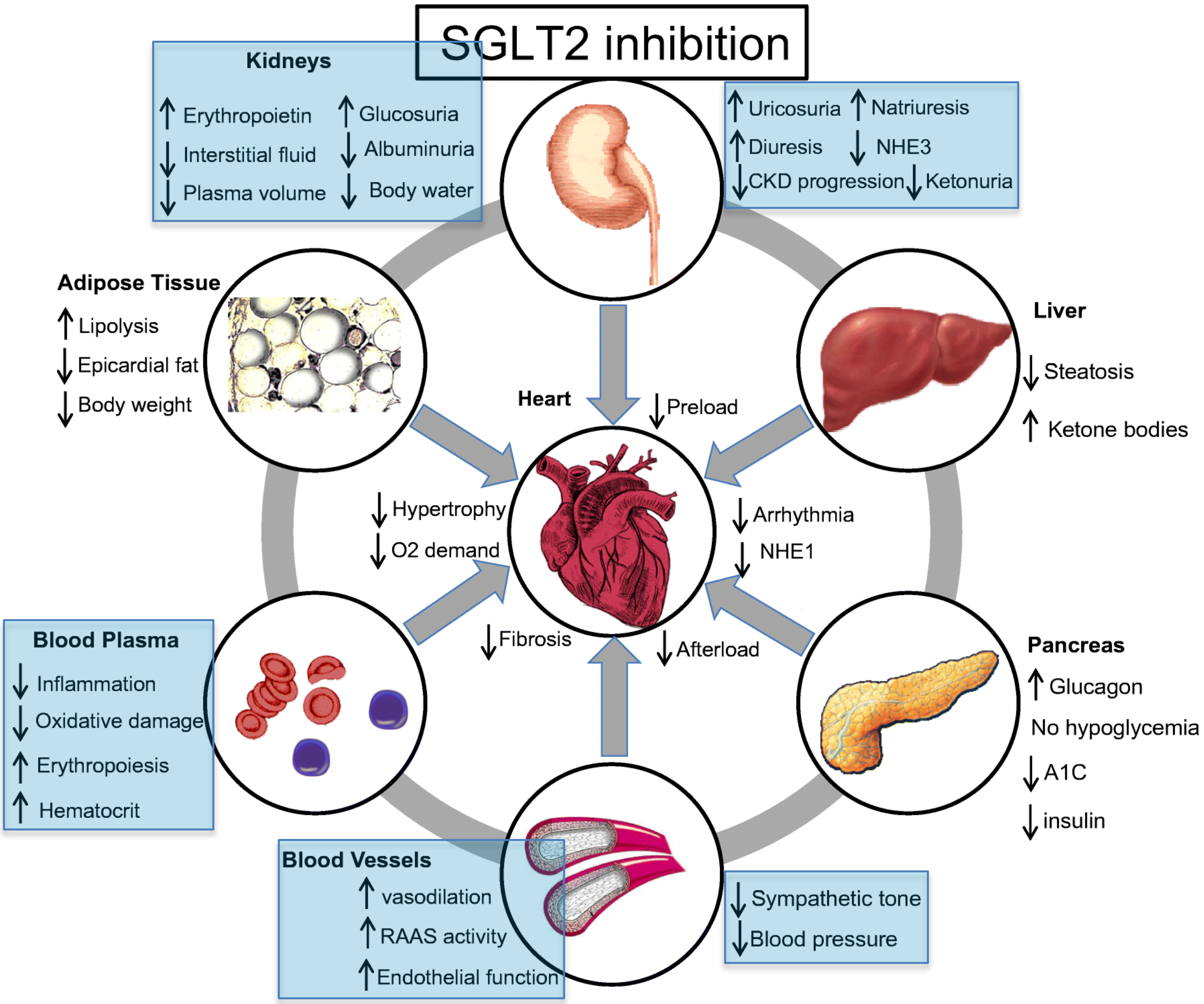

Fig. 7: A schematic representation of the different mechanisms implicated in the cardiovascular benefits of SGLT2 inhibitors. The blue squares underline the mechanistic structures affected by SGLT2-is with volume modulating potential (modified from Wojcik et al. [55]).

3.1.3.3 *Linking relative plasma volume status to myocardial energy metabolism to cardio-protection in heart failure*

Albeit, it is clear that various mechanisms mediate the cardio- but also reno-protective effect of SGLT-2is in heart failure the underlying key mechanisms still have to be uncovered.

As stated before, there is accumulating evidence from several recent studies for the beneficial effects of SGLT-2is on blood volume regulation, body fluid balance and volume distribution, which may play a key role in mediating SGLT-2is induced cardio-protection. These 'volume-based' alterations may improve systemic and cardiac hemodynamics as well as oxygen transport (increased erythropoiesis) leading to improved tissue oxygen supply at lower relative energetic costs- particularly in the heart (Fig. 8).

Under physiological conditions the myocardial oxygen demand and supply via the coronary vessels are well-balanced to maintain a proper heart function- continuously without a brake [76]. However, in heart failure this balance between fuel demands and supply is frequently disrupted by multiple factors.

For example, on the supply side, pre-existing coronary artery disease (CAD) is associated with reduced coronary blood flow and subsequently oxygen supply to the cardiac muscle causing ischemic heart disease (IHD), a major risk factor for heart failure [77]. On the demand side, volume overload and congestion induce increased myocardial (increased cardiac preload) and systemic stress (increased sympathetic activity), which results in an increased oxygen demand of the heart [78].

Additionally, volume overload leads to functional (e.g. diastolic dysfunction) and structural alterations

(e.g. cardiac remodelling and myocardial hypertrophy) within the heart resulting in increased ventricular stiffness with negative consequences on myocardial energetic efficiency [78, 79].

Furthermore, VO induces increased mitochondrial oxidative stress, cytoskeletal disruption and mitochondrial damage in cardiomyocytes, which contributes to myocardial malfunction in heart failure [78, 80].

Following the 'volume-based' hypothesis, the treatment with SGLT-2is may improve the relative plasma volume status (PVS) in HF patients, especially after being hospitalized due to an ADHF event.

An improved PVS is defined by a reduced deviation of the actual plasma volume (aPV) to the calculated ideal plasma volume (iPV) and might be the result of a complex interplay between multiple factors and regulatory systems with the cardio-renal-axis as mechanistic epicentre.

As the PVS was shown to have prognostic implications in HF patients, the potential effects of SGLT-2is on PVS may help to prevent adverse clinical outcomes, e.g. worsening heart failure and re-hospitalization in these patients.

However, the hypothetical assumption of an improved PVS after dapagliflozin treatment on top of recommended therapy in comparison to placebo with its potential beneficial effects on cardiac and vascular function in clinically stabilized HF patients after hospitalization because of an ADHF event needs to be confirmed in a clinical trial setting.

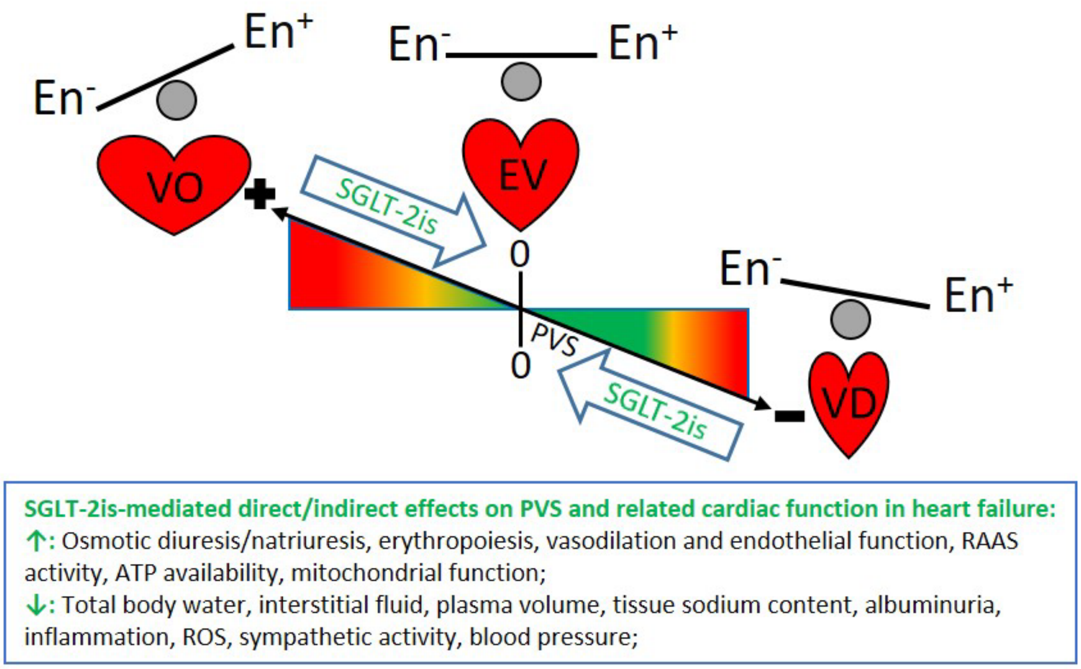

Fig. 8: SGLT2-is treatment effect in clinically stabilized HF patients after an ADHF event- a 'volume-based' hypothesis. Persistent volume overload (VO) and congestion at clinical discharge are frequently present in HF-patients after hospitalization because of and ADHF event but treatment induced volume depletion (VD) may also occur. VO is reflected by a positive (**+**) relative plasma volume status (PVS) and VD by a negative (**-**) PVS on the double-arrowed PVS-axis. The color code on the PVS axis indicates the increase in hazard ratio (HR) (from green low to red high) for hospitalization and all-cause mortality in HF patients dependent on the percent deviation from 'euvolemia' (EV, PVS= 0). VO induces increased myocardial stress with negative functional and structural consequences for the heart. These cardiac alterations can lead to an imbalance between cardiac energetic demands (En^-) and supply (En^+), which may result in cardiac energy depletion, cardiac malfunction and worsening heart failure (WHF). Treatment with SGLT-2is reduce VO and eventually also VD and thus improve PVS by shifting it towards 'euvolemia' (indicated by the blue arrows) via a complex interaction with the cardio-renal-axis (CRA). The SGLT-2is induced reduction in VO (VD) and concomitant increase in erythropoiesis may lead to improved cardiac hemodynamics and tissue oxygen supply with beneficial effects on cardiac function and myocardial energy homeostasis.

3.2 Investigational product and indication

3.2.1 Drug description and treatment indication

Dapagliflozin propanediol (FORXIGA™) is a drug developed by AstraZeneca AG (for detailed information see also the actual Investigator's Brochure provided by the company). Dapagliflozin is orally available and requires once-daily dosing.

Dapagliflozin (DAPA) is a highly potent, selective, and reversible inhibitor of SGLT2 that improves glycemic control in patients with diabetes mellitus and provides cardio-renal benefits in patients with type 2 diabetes mellitus (T2DM) and without diabetes.

Dapagliflozin is currently undergoing regulatory review for marketing authorizations(s) for use in adults for the treatment of HFrEF.

In Switzerland, dapagliflozin is approved to treat T2DM, either as monotherapy or as add-on combination therapy with blood glucose lowering drugs (for detailed information see also the actual Swissmedic approved national professional information for A10BK01 dapagliflozin [81]).

Dapagliflozin film-coated tablets used in clinical studies are available in strengths of 2.5, 5, and 10 mg. The tablets contain dapagliflozin propanediol (BMS-512148) drug substance, microcrystalline cellulose, anhydrous lactose, crospovidone, silicon dioxide, magnesium stearate, and coating material

(Opadry® II). The physical and chemical properties are provided in table 2.

Table 2 Physical and chemical properties of dapagliflozin.

Substance code	BMS-512148-05
Molecular formula	C21H25ClO6•C3H8O2•H2O
Formula weight	502.98
Molecular weight	408.87 (dapagliflozin)
Appearance	White to off-white powder which may contain lumps
pH	6.9 (1.5 mg/mL)
pKa	Non-ionizable compound

3.2.2 Pharmacokinetics and drug metabolism in humans

3.2.2.1 *Drug absorption and distribution*

Dapagliflozin is rapidly and well absorbed after oral administration and can be administered with or without food. Maximum dapagliflozin plasma concentrations are usually attained within 2 hours after administration in the fasted state. The C_{max} and AUC values increase proportionally to the increment in dapagliflozin dose. The absolute oral bioavailability of dapagliflozin following the administration of a 10 mg dose is 78%. Food has relatively modest effects on the PK of dapagliflozin in healthy subjects. Administration with a high-fat meal decreases dapagliflozin C_{max} by up to 50% and prolonged t_{max} by approximately 1 hour, but does not alter AUC as compared with the fasted state. These changes are not considered to be clinically meaningful.

Dapagliflozin is approximately 91% protein bound. Protein binding is not altered in various disease states (eg, renal or hepatic impairment).

3.2.2.2 *Drug metabolism and elimination*

Dapagliflozin is a C-linked glucoside, meaning the aglycone component is attached to glucose by a carbon-carbon bond, thereby conferring stability against glucosidase enzymes. The mean plasma t1/2 for dapagliflozin is 12.9 hours following a single oral dose of dapagliflozin 10 mg to healthy subjects. Dapagliflozin is extensively metabolized, primarily to yield dapagliflozin 3-O-glucuronide, which is an inactive metabolite. Dapagliflozin 3-O-glucuronide accounted for 61% of a 50 mg [^{14}C]-dapagliflozin dose and is the predominant drug-related component in human plasma, accounting for 42% (based on AUC 0-12 hour]) of total plasma radioactivity, similar to the 39% contribution by parent drug. Based on AUC, no other metabolite accounts for > 5% of the total plasma radioactivity. Dapagliflozin 3-O-glucuronide or other metabolites do not contribute to the glucose-lowering effects. The formation of dapagliflozin 3-O-glucuronide is mediated by UGT1A9, an enzyme present in the liver and kidney, and CYP-mediated metabolism is a minor clearance pathway in humans.

Dapagliflozin and related metabolites are primarily eliminated via urinary excretion, of which less than 2% is unchanged dapagliflozin. After administration of 50 mg [^{14}C]-dapagliflozin dose, 96% is recovered; 75% in urine and 21% in feces. In feces, approximately 15% of the dose is excreted as parent drug.

Look-alike placebo tablets are supplied in the same container/closure system. The placebo tablets contain lactose monohydrate, microcrystalline cellulose, magnesium stearate, and coating material (Opadry® II).

3.3 Preclinical evidence

N.A. (detailed preclinical information about dapagliflozin can be obtained from the investigators brochure provided by AstraZeneca AG).

3.4 Clinical evidence

3.4.1 Dapagliflozin on primary composite outcome of DAPA-HF trial

The DAPA-HF trial (clinicaltrials.gov identifier: NCT03036124) was an international, multicenter, randomized, double-blind, placebo-controlled study in patients with heart failure (NYHA functional class II-IV) with reduced ejection fraction (LVEF ≤ 40%) to determine the effect of dapagliflozin compared with placebo, when added to background standard of care therapy, on the incidence of cardiovascular (CV) death and worsening heart failure [5, 82].

The overall study objective was to determine whether dapagliflozin prevents CV death and worsening heart failure, and if dapagliflozin improves heart failure symptoms.

Of 4744 patients, 2373 were randomized to dapagliflozin 10 mg and 2371 to placebo and followed for a median of 18 months. The mean age of the study population was 66 years, 77% were male, 70% White, 5% Black or African-American, and 24% Asian.

At baseline, 67.5% patients were classified as NYHA class II, 31.6% class III, and 0.9% (43/4744 patients) class IV, median LVEF was 32%, 42% of the patients in each treatment group had a history of T2DM, and an additional 3% of the patients in each group were classified as having T2DM based on a HbA1c ≥ 6.5% at both enrolment and randomization.

Patients were on standard of care therapy; 94% of patients were treated with an ACE inhibitor, ARB, or ARNI, 11% with ARNI, 96% with beta-blocker, 71% with mineralocorticoid receptor antagonist, 93% with diuretic, and 26% had an implantable device.

As stated previously, DAPA-HF was the first trial to show that the treatment with dapagliflozin improves clinical outcomes in heart failure patients with reduced left ventricular ejection fraction (HFrEF) independent of presence or absence of diabetes and therefore considered as a ‘breakthrough’ in the search to treat HFrEF patients [83].

The study authors of DAPA-HF [5] reported the main study results (Fig. 9) as follows:

‘Over a median of 18.2 months, the primary outcome occurred in 386 of 2373 patients (16.3%) in the dapagliflozin group and in 502 of 2371 patients (21.2%) in the placebo group (hazard ratio, 0.74; 95% confidence interval [CI], 0.65 to 0.85; P<0.001). A first worsening heart failure event occurred in 237 patients (10.0%) in the dapagliflozin group and in 326 patients (13.7%) in the placebo group (hazard ratio, 0.70; 95% CI, 0.59 to 0.83).

Death from cardiovascular causes occurred in 227 patients (9.6%) in the dapagliflozin group and in 273 patients (11.5%) in the placebo group (hazard ratio, 0.82; 95% CI, 0.69 to 0.98); 276 patients (11.6%) and 329 patients (13.9%), respectively, died from any cause (hazard ratio, 0.83; 95% CI, 0.71 to 0.97). Findings in patients with diabetes were similar to those in patients without diabetes (Fig. 10).

Importantly, the frequency of adverse events related to volume depletion, renal dysfunction, and hypoglycemia did not differ between treatment groups’.

The study authors concluded that ‘among patients with heart failure and a reduced ejection fraction, the risk of worsening heart failure or death from cardiovascular causes was lower among those who received dapagliflozin than among those who received placebo, regardless of the presence or absence of diabetes’ [5].

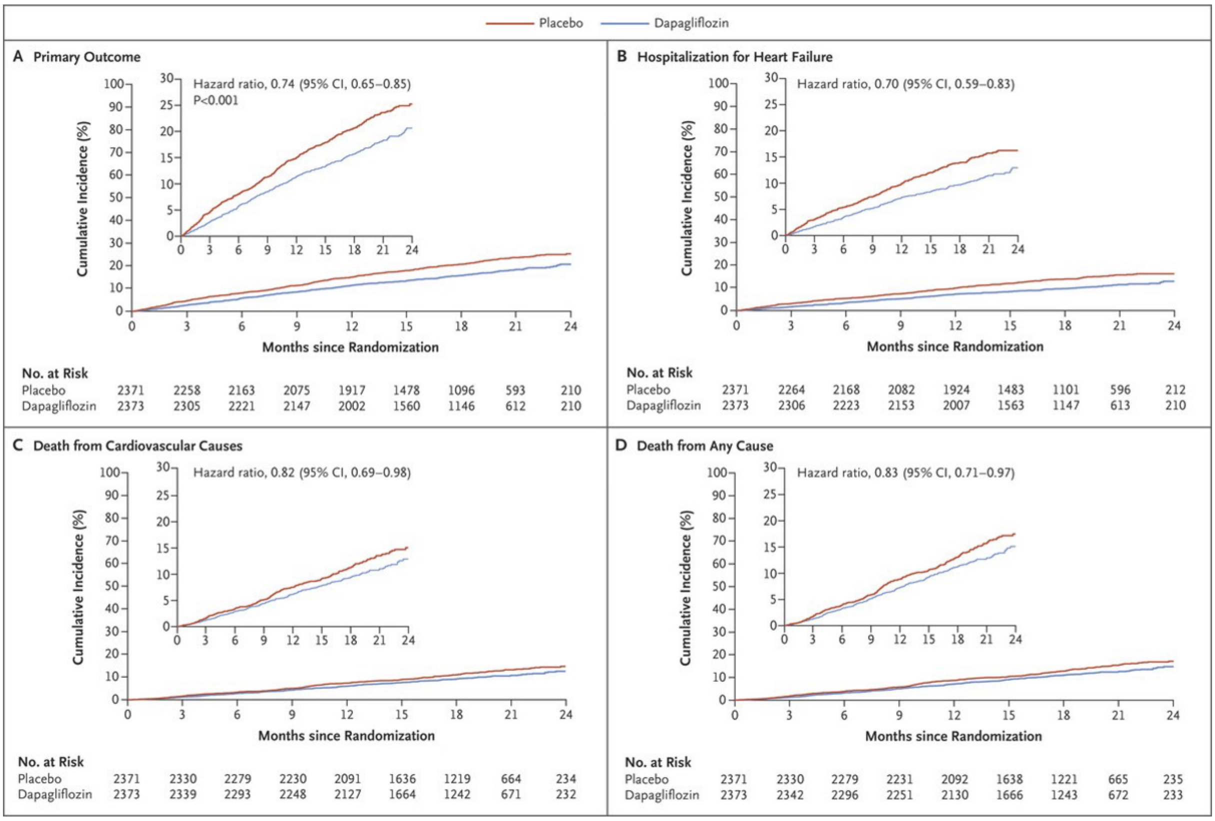

Fig.9: Cardiovascular Outcomes of DAPA-HF. The primary outcome was a composite of death from cardiovascular causes, hospitalization for heart failure, or an urgent visit resulting in intravenous therapy for heart failure (Panel A). The cumulative incidences of the primary outcome, hospitalization for heart failure (Panel B), death from cardiovascular causes (Panel C), and death from any cause (Panel D) were estimated with the use of the Kaplan–Meier method; hazard ratios and 95% confidence intervals were estimated with the use of Cox regression models, stratified according to diabetes status, with a history of hospitalization for heart failure and treatment-group assignment as explanatory variables. Included in these analyses are all the patients who had undergone randomization. The graphs are truncated at 24 months (the point at which less than 10% of patients remained at risk). The inset in each panel shows the same data on an enlarged y axis (adapted from McMurray et al. [5]).

A Patients without diabetes

Primary composite outcome	No. of patients/total No. (%) Dapagliflozin	Placebo	Absolute risk reduction (95% CI), %	Hazard ratio (95% CI)	P value for interaction
Overall effect	386/2373 (16.3)	502/2371 (21.2)	4.9 (2.7 to 7.1)	0.74 (0.65 to 0.85)	
Type 2 diabetes at baseline[a]					
Yes	215/1075 (20.0)	271/1064 (25.5)	5.5 (1.9 to 9.0)	0.75 (0.63 to 0.90)	.80
No	171/1298 (13.2)	231/1307 (17.7)	4.5 (1.7 to 7.3)	0.73 (0.60 to 0.88)	
Patients without type 2 diabetes at baseline					
Normoglycaemic	53/438 (12.1)	71/419 (16.9)	4.8 (0.1 to 9.6)	0.67 (0.47 to 0.96)	.72
Prediabetes	118/860 (13.7)	160/888 (18.0)	4.3 (0.9 to 7.7)	0.74 (0.59 to 0.94)	

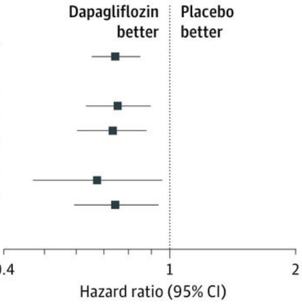

Fig. 10: Primary composite end point based on diabetes status and glycated hemoglobin in a study of the effect of dapagliflozin on worsening heart failure and cardiovascular death in patients with heart failure with and without diabetes. [a] In patients without diabetes, analysis of patients with a glycated hemoglobin greater than or equal to 5.7% (prediabetes) and less than <5.7% (normoglycemia) was prespecified (modified from Petrie et al. [50]).

3.4.2 Additional findings of DAPA-HF sub-analyses and DEFINE-HF outcomes

A recent post hoc sub-group analysis of DAPA-HF data revealed that the beneficial effect of dapagliflozin on the primary composite outcome was consistent regardless of background therapy for HF [84], which may indicate a unique mechanism by which the drug mediates its beneficial effects on the heart and the kidney.

Another recent sub-analysis on data obtained from DAPA-HF focused on the effects of dapagliflozin on a broad range of health status outcomes using the Kansas City Cardiomyopathy Questionnaire (KCCQ) and revealed that patients treated with dapaglifozin had a clinical meaningful greater improvement in mean KCCQ- total symptoms score (TSS), clinical summary score, and overall summary score at 8 months observation period (2.8, 2.5 and 2.3 points higher versus placebo; P<0.0001 for all) and improved symptom burden, functional status, and quality of life in comparison to placebo [85].

Interestingly, Martinez et al. [86] found in a sub-analysis of the DAPA-HF data found that dapagliflozin reduced the risk of death and WHF and improved symptoms across a broad spectrum of age with no imbalance in tolerability or safety events even in the elderly.

The DEFINE-HF (clinicaltrials.gov identifier: NCT 02653482) was an IIT, multi-center, randomized controlled trial of patients with HFrEF, New York Heart Association (NYHA) class II-III, estimated glomerular filtration rate ≥30 mL/min/1.73m^2, and elevated natriuretic peptides. In total, 263 patients were randomized to dapagliflozin 10 mg daily or placebo for 12 weeks. Dual primary outcomes were (1) mean NT-proBNP (N-terminal pro b-type natriuretic peptide) and (2) proportion of patients with ≥5-point increase in HF disease-specific health status on the Kansas City Cardiomyopathy Questionnaire (KCCQ) overall summary score, or a ≥20% decrease in NT-proBNP.

Albeit, there was no significant difference in average 6- and 12-week adjusted NT-proBNP with dapagliflozin versus placebo (1133 pg/dL (95% CI 1036–1238) vs 1191 pg/dL (95% CI 1089–1304), P=0.43) the second dual-primary outcome of a meaningful improvement in the KCCQ met this end point with a overall summary score of 61.5% of dapagliflozin-treated patients versus 50.4% with placebo (adjusted OR 1.8, 95% CI 1.03-3.06, nominal P=0.039).

The results were consistent among patients with or without type 2 diabetes mellitus, and other pre-specified subgroups (all P values for interaction= NS).

3.5 Dose rationale: Rationale for the intended purpose in study

Dapagliflozin will be provided as film-coated tablets containing 10mg of dapagliflozin propanediol per tablet, which will be orally taken by the patients, once daily (1 tablet/day) and on top of recommended therapy for 12 weeks in total.

The dosing of dapagliflozin (10mg/day) is based on the dosage regimen used in major trials, e.g. DAPA-HF [5] and DEFINE-HF [87], which found beneficial treatment effects of dapagliflozin in HF-patients as described previously and also reported that treatment regimen was well-tolerated by the patients.

Furthermore, the dosing follows the recommendations of the Swissmedic approved national professional information sheet [81] as listed in the Swiss drug compendium (https://compendium.ch).

3.6 Explanation for choice of placebo

The comparator chosen for the study is placebo. The study treatment (dapagliflozin, placebo) will be administered on top of recommended therapy, which ensures that all HF-patients are treated according to the actual therapeutic guidelines for heart failure [11]. The effect of this class of drugs is quiet unique and therefore no alternative active comparator is currently available to compare with the study drug. Furthermore, the period of potential placebo treatment is relatively short (3 months) in comparison to other trials. Therefore, we think that placebo is ethically justified to choose.

3.7 Risks / benefits

Dapagliflozin has been recently shown in a major trial (DAPA-HF) to improve clinical outcomes (e.g. reduction in mortality and worsening heart failure) and quality of life in heart failure patients with or without diabetes (see also 3.4).

Therefore, it is a very promising drug for the future treatment of a brought spectrum of HF-patients.

Indeed, dapgliflozin got recently FDA-approval for the treatment of adult patients with HFrEF to reduce cardiovascular death and hospitalization for heart failure [88].

However, it is also of utmost importance to investigate and uncover the potential key mechanisms that mediate the observed cardio-protective and also reno-protective effects of SLGT-2is such as dapagliflozin. A better mechanistic understanding of the drug may help to improve actual therapeutic strategies and may also help to avoid potential adverse events, e.g. due to inadequate drug combinations or indications.

Importantly, dapagliflozin was shown to be well-tolerated in large cohorts (more than 15.000) of diabetes patients but also in more than 2000 HF-patients (see also investigators brochure). The safety profile of dapagliflozin was overall consistent across the studied indications.

The most common observed side effects in a 13-study short-term, placebo-controlled pooled cohort were genital infections (GI, DAPA: 5.5% vs. PLA: 0.6%) and urinary tract infections (UTI, DAPA: 4.7% vs. PLA: 3.5%). However, these infections were usually mild and mostly responded to initial standard treatment and furthermore rarely resulted in discontinuation from the study (GI, DAPA: 0.2% vs. PLA: 0%, UTI, DAPA: 0.2% vs. 0.1%).

Of note, a recent meta-analysis of the data of the 2 major trials in heart failure with and without diabetes (DAPA-HF, EMPEROR-reduced) revealed that the beneficial effects of SGLT2-inhibitors were highly consistent, irrespective of diabetes status, age, sex, eGFR, and concomitant therapy and that the descriptive adverse event profile was comparable to placebo [89].

On the other hand, the planed measurements and interventions (e.g. blood volume, vascular function, etc., see also 9.2) are all non-invasive (except from venous blood sample collection) and don't involve radiation. Based on yearlong internal experience they are generally well-tolerated by the study participants and do pose a very low risk.

For example, allergic reactions to glycerol trinitrate (GTN) or tropicamide eye drops are possible but very rare (see also 9.2). Rarely GTN can induce transient symptomatic hypotension. Blood pressure will be measured before and after the test and care will be taken that patients don't get up from the supine position too quickly to avoid orthostatic dysregulation.

In summary, the risk/benefit ratio for the participants in this study is reasonable in relation to the anticipated knowledge gained from the study results.

3.8 Justification of choice of study population

The study will recruit adult patients with a diagnosis of de novo or chronic heart failure that have been clinically stabilized (planed for clinical discharge) after encountering an acute decompensated heart failure (ADHF) event. The study treatment has been studied in HF-patients and has been recently approved for the treatment of HFrEF-patients with and without diabetes on top of recommended standard therapy. A recent retrospective analysis of the DAPA-HF study revealed that the absolute risk reduction of cardiovascular events and worsening of heart failure was even more pronounced in patients with a recent ADHF event [90]. Of importance, the time to clinical benefit of Dapagliflozin treatment was 28 days post treatment start [90]. The study results of the EMPA-RESPONSE-AHF trial (NCT03200860) showed that the use of the SGLT2-inhibitor Empagliflozin was well-tolerated and most importantly safe also in de novo ADHF patients (proportion of de novo AHF in trial: 47%) [91]. Additionally, the follow-up analysis revealed a reduction in the combined clinical outcome including worsening heart failure (WHF), re-hospitalization and death [91]. A multi-centre clinical trial currently investigating the effects of Dapagliflozin treatment in acute heart failure (DICTATE-HF: NCT04298229) including de novo HF is currently ongoing [92]. Furthermore, another active trial investigates the effects of Dapagliflozin on top of recommended standard therapy on hospitalization for heart failure (HHF) or cardiovascular (CV) death in patients with acute myocardial infarction (DAPA-MI, NCT04564742).

To summarize, growing research evidence suggests that patients with either de novo or chronic HF may profit from the treatment with SGLT2-inhibitors starting in the early phase after an ADHF event and thereafter. On the other hand, the use of SGLT2-inhibitors in these patients was shown to be well-tolerated and safe in these patients. However, the underlying mechanistic background of the beneficial drug effects are still strongly debated and yet not fully understood and thus need to be further investigated to optimize the implementation of SGLT2-inhibitors in the recommended therapy of HF-patients. Therefore, the choice of study population is justified. The study does not recruit vulnerable participants.

4. STUDY OBJECTIVES

4.1 Overall Objective

The general purpose of the DAPA-VOLVO study project is to evaluate the effects of dapagliflozin on top of recommended therapy and in comparison to placebo on volume status and on vascular function as well as its mechanistic interactions from an integrative perspective in clinically compensated heart failure patients after hospitalization/ambulatory care because of an acute decompensated (congestive) heart failure event.

The study results may contribute to a better understanding of the underlying mechanisms that mediate the cardio-protective effect of the drug in heart failure patients.

4.2 Primary Objective

The study evaluates the effects of dapagliflozin on blood volume (BV) profile measured via optimized CO-rebreathing (OpCO) technique and on vascular function assessed via retinal vessel analysis (RVA) and pulse wave analysis (PWA) in clinically compensated heart failure patients after hospitalization due to an acute decompensated (congestive) heart failure (ADHF) event.

4.3 Secondary Objectives

The study also seeks to determine whether the effects of dapagliflozin on blood volume profile and vascular function translates to structural and functional improvements of the heart (e.g. exercise performance) and improvements in patient's health-related quality of life (e.g. KCCQ score) as well as in heart failure related laboratory parameters (e.g. NT-proBNP) in comparison to placebo. Furthermore, the study aims to provide further mechanistic insight into the cardio-protective effects of dapagliflozin in HF-patients by additionally evaluating body fluid distribution, autonomic function (MSNA), tissue sodium content (Na^+-MRI) and novel laboratory parameters for heart failure (e.g. FGF23).

4.4 Safety Objectives

The study aims to assess whether dapagliflozin leads to a differential adverse profile (e.g. incidences of hypotension, renal dysfunction, genital infections (GI) and urinary tract infections (UTI), diabetic ketoacidosis, etc.) and general adverse event profile in comparison to placebo.

5. STUDY OUTCOMES

5.1 Primary Outcome

The primary study outcome is the mean change in relative plasma volume status (ΔPVS) from baseline to 12 weeks of dapagliflozin treatment in comparison to placebo.

Rationale: The PVS was shown to be associated with prognostic outcomes in heart failure patients in terms of hospitalization and mortality [9, 10, 37]. Furthermore, the estimated PVS at hospital discharge was independently associated with post-discharge clinical outcomes in 3 different ADHF cohorts [93]. Thus, any therapeutic intervention that leads to an improvement in PVS as reflected by shift towards euvolemia (PVS= 0) may also lead to improved clinical outcomes in HF-patients. Following the 'volume'-based hypothesis, the treatment with dapagliflozin may shift and also stabilize the PVS in HF-patients at levels closer to euvolemia.

5.2 Secondary Outcomes

- Blood volume profile (Optimized CO-rebreathing, OpCO) ¤;
- Body water content and distribution (Bioimpedance analysis, BIA) ¤;
- Retinal vessel function (Retinal vessel analysis, RVA) §;
- Arterial stiffness (Pulse wave analysis, PWA) §;
- Endothelial function (Flow-mediated dilation, FMD) §;
- HF-hospitalization (either or: worsening of heart failure, WHF*) °;
- HF-related quality of life (Kansas City cardiomyopathy questionnaire, KCCQ) °;
- Percentage of patients being within the optimal PVS (% PVS_{opt} #) °;

* WHF: Requiring intensification of therapy in the ambulatory or hospital setting including i.v. diuretics, i.v. nitrates, or other medications for HF, or institution of mechanical or ventilator support.

# %PVS_{opt}: Percent of patients within proposed optimal PVS divergence interval for HF as defined by the combined divergence interval with the lowest reported hazard ratio (H_R) for HF-hospitalization and mortality: -4% to -18% of iPV [9, 10]);

¤ Primary volume-related outcomes, § Primary vascular function-related outcomes, ° Other outcomes;

Rationale: The secondary outcomes are add-on study measures, which are directly or indirectly linked to the primary study outcome (PVS) and are supposed to be altered by dapagliflozin treatment according to scientific literature.

Thus, these parameters may help to provide a more detailed mechanistic insight into the effects of dapagliflozin treatment on volume status and vascular function as potential mediators of cardio-protection.

Additionally, the effects of dapagliflozin treatment on selected clinical outcomes will be also assessed and potential associations to measured functional parameters will be evaluated.

5.3 Other Outcomes of Interest

Tertiary Outcomes:

- Exercise capacity (CPET: VO_{2peak}, $VEVCO_{2slope}$, 6MWD) °;
- Left ventricular morphology and function (TTE: LVM, LVEF) °;
- Sympathetic activity (MSNA) °;
- Tissue sodium content (Na-MRI: skin, muscle, cartilage) ¤;
- Renal perfusion and oxygenation (ASL ^{1}H-MRI and BOLD MRI) °;
- Total amount of albumin and other blood constituents (OpCO) ¤;
- Cardio-renal biomarkers (e.g. FGF23) °;
- Iron metabolic biomarkers (serum ferritin, TSAT, hepcidin) °;

Rationale: The tertiary study outcomes are supposed to be affected by dapagliflozin treatment according to available scientific literature. The selected study measures may provide an even more advanced and partially novel mechanistic insight into the effects of dapagliflozin treatment on cardiac and vascular function. Furthermore, the tertiary outcomes will evaluate whether the treatment with dapagliflozin has beneficial effects on cardiac function (e.g. LVEF) and exercise performance (e.g. VO_{2peak}) in comparison to placebo in this particular cohort of heart failure patients.

5.4 Safety Outcomes

The following safety outcomes of the main study (V2-V5) are defined:

- AEs/SAEs;
- Renal function (eGFR);
- Incidence/events of hypotension (systolic BP < 90mmHg in 3 consecutive measurements);
- Incidence/events of GI-/UTI-infections;
- Incidence/events of diabetic ketoacidosis (DKA);
- Incidence/events of Fournier gangrene (FG);
- Incidence/events of back pain;
- Laboratory biomarker of heart failure (NT-proBNP);
- Other relevant laboratory parameter;

Rationale: The patient’s safety has absolute priority. The defined safety outcome variables are primarily chosen based on the safety reports of previous trials in HF-patients (e.g. DAPA-HF).

Of note, the defined safety outcomes will not be assessed after the end of the main study period (V5).

6. STUDY DESIGN

6.1 General study design and justification of design

6.1.1 General study design

The DAPA-VOLVO study project is a randomized, double-blind, placebo-controlled parallel-group trial to evaluate the superiority of dapagliflozin on top of recommended therapy in comparison to placebo on blood volume profile and vascular function in clinically stabilized heart failure patients after hospitalization/ ambulatory care because of an acute decompensated (congestive) heart failure event.

A total of 80 HF-patients (40 patients per each treatment arm) will be recruited at the University Hospital Zurich.

DAPA-VOLVO- Main study:

The main study will comprise of 5 visits at the study site within the study observation period, albeit V1 will be integrated into V2 whenever logistically feasible. The primary goal must be to follow the planed study frame, however if for particular reasons a study patient can only be enrolled in the short-term post hospitalization phase, e.g. due to the patients wish because of stress overload induced by clinical interventions/procedures during the hospitalization/ ambulatory care phase, a note to file should be generated incl. the reason for the 'postponed' study start.

The relative visit schedule should be maintained as planned (see Fig.1, Table 1).

Important: The study information (SI) must be provided to the potential study candidate at least 24 hours prior to V1. The main study ends after the completion of V5, which also represents the end of IMP-treatment.

DAPA-VOLVO- Advanced session(s):

In order to get a better mechanistic insight into the dapagliflozin effect in HF-patients after an ADHF event an optional advanced session(s) program is implemented. The patients can voluntary choose on the informed consent sheet via check box whether they want to participate or not. The program (Fig. 13) comprises additional tests (e.g. CPET) in close proximity to V2 (V2a) and V5 (V5a).

At visit 1 (V1, screening visit, -5d to -1d)) after signing the informed consent, the patients will be evaluated first against the inclusion and exclusion criteria (see 7.1). Eligible patients will be enrolled into the study and randomized to 12 weeks of daily oral dapagliflozin treatment (10mg/day) or placebo. Also, a short medical anamnesis and a short standard physical examination will be performed including venous and urine sampling for laboratory analysis and the dates for the baseline visit and follow-up visits will be arranged. Of note, V1 will be integrated into V2 whenever this is logistically feasible/possible.

At visit 2 (V2, baseline visit, -2d to 0d), the primary outcome and secondary/tertiary outcomes will be assessed at baseline (see 9.) after a short medical anamnesis and physical examination. Additionally, the venous blood and urine samples will be collected for laboratory analysis. Of note, the baseline measurement of the primary study outcome (PVS) should be performed ≤ 48 hours prior to treatment start (0d).

Treatment start (d0):

The treatment will start after the baseline measurements are finished, however depending on the clinical setting/ status of the patients the treatment start may be delayed but the reason for the delay must be documented (e.g. via note to file). The patients will be introduced to continue the medication for the study duration but to maintain a time gap of at least 12 hours between each drug intake.

At visit 3 (V3, Follow-up visit, 2w ± 5d post treatment start), the primary outcome and selected secondary and tertiary outcomes including safety outcomes will be assessed after a short medical anamnesis and physical examination. Additionally, the venous blood and urine samples will be collected for laboratory analysis.

At visit 4 (V4, Follow-up visit, 6w ± 5d post treatment start), the visit procedures will be repeated similar to visit 2.

At visit 5 (V5, Follow-up visit, after 12 weeks (± 5d) of treatment), patients will be invited for the final study visit to re-evaluate the primary, secondary and tertiary study outcomes as well as defined safety outcomes similar to baseline visit. At the end of the study, the IMP-treatment will be stopped and regular recommended therapy as clinically indicated will be continued in all patients.

Advanced session(s) (V2a/V5a):

The study patients who are willing to participate will perform additional tests in the advanced session(s) program (see Fig. 13) either at the same day or in close as possible/ feasible proximity to V2 (V2a) and V5 (V5a) but prior to treatment start. In dependence of the feasibility/capacity, the additional program includes tests to evaluate the exercise capacity, cardiac- and autonomic function as well as MRI-imaging (see also 9.).

DAPA-VOLVO- Follow-U (V6):

The prospective observational follow-up study will include another optional visit (V6) at the study site to re-evaluate selected outcome measures of the main study 12 months (± 2w) after the main study end and treatment stop with the IMP (V5). The patients can decide by marking the corresponding check box on the informed consent sheet, whether they are willing to also participate in the follow-up study or not.

Of note, the follow-up study will be evaluated and analysed separately from the main study (see also 6.1.1, 11.4.1, Fig. 1, Table 1).

Table 3. Study tasks and dates (ideal recruitment rate).

Task	Date
First patient first visit (FPFV)	04/2021
Last patient first visit (LPFV)	07/2022
Last patient last visit (LPLV)	10/2022
First study results (FSR)	11/2022
Submission of 1st manuscript	01/2023

6.1.2 Justification of study design

The study design of the DAPA-VOLVO project follows the actual GCP guidelines [14] for RCTs and is considered as adequate for the current study purpose. The main study limitation is the relative small sample size, however a priori sample size calculation for the primary outcome was performed using a statistical test (MMRM) with a relatively 'robust' analysis to minimize the limitation factor (see 11.2).

6.2 Methods of minimising bias

6.2.1 Randomisation

Eligible patients will be randomized to dapagliflozin treatment group or placebo group in a 1:1 allocation ratio using block randomization. Randomization will be performed with a random computerized algorithm using the secuTrial ® software system via interactive response technology (IRT). The investigator or his/her delegate will contact the IRT after confirming that the patient fulfils all the inclusion and none of the exclusion criteria. The IRT will assign a randomization number to the patient, which will be used to link the patient to a treatment arm and will specify a unique medication number for the first package of investigational treatment to be dispensed to the patient. The randomization list is generated by the secuTrial ® software system and responsible staff at the Clinical Trial Center Zurich. The secuTrial ® software system ensues that the randomization list is concealed from the study team and investigators (unless in case of necessary unblinding as depicted in 5.4).

6.2.2 Blinding procedures

Patients, investigator staff, persons performing the assessments and data analysts will remain blind to the identity of the treatment from the time of randomization until database lock, using the following methods:

1. Randomization data are kept strictly confidential until the time point of unblinding, and will not be accessible by anyone involved in the study.
2. The identity of the treatments will be concealed by the use of investigational treatment that are all identical in packaging, labelling, schedule of administration, appearance, taste and odor.

6.2.3 Other methods of minimising bias

N.A.

6.3 Unblinding procedures (code break)

A code break will be available to the investigator and is supplied by the secuTrial® software system. This code break should be opened only in emergency situations when the identity of the investigational product must be known by the investigator in order to provide appropriate medical treatment. For any patient whose treatment code has been broken the patient must discontinue the study treatment.

7. STUDY POPULATION

Patients with a diagnosis of de novo or chronic heart failure and clinically stabilized (planed for hospital discharge) after hospitalization/ ambulatory care because of an acute decompensated (congestive) heart failure (ADHF) event will be enrolled in this study. The total number of participants is 80, studied at one treatment site (University Hospital Zurich). During the enrolment period of 20 months, we expect to include an average of 4.1 patients per month, which corresponds to 1 patient per week (optimal recruitment conditions).

If the planed enrolment goals are not met, the enrolment period may be extended and additional staff allocated to the study and/or another medical study centre within Switzerland or outside might be recruited to guarantee sufficient numbers of enrolled patients for primary study outcome.

7.1 Eligibility criteria

7.1.1 Inclusion criteria

Participants fulfilling all of the following <u>inclusion</u> criteria are eligible for the study, for example:

1. Male or female, age of 18years or older;
2. Patients with documented diagnosis of de novo or chronic heart failure (NYHA II-IV) and clinically stabilized (considered for hospital discharge) after hospitalization/ ambulatory care because of an acute decompensated (congestive) heart failure (ADHF) event;
3. eGFR ≥ 30 mL/min/1.73 m^2 (CKD-EPI formula) at enrolment;
4. Provision of signed informed consent prior to any study specific procedure;

7.1.2 Exclusion criteria

The presence of any one of the following <u>exclusion</u> criteria will lead to exclusion of the participant, for example:

1. Contraindications to the class of drugs under study, e.g. known hypersensitivity or allergy to class of drugs or the investigational product;
2. Receiving therapy with an SGLT2 inhibitor within 8 weeks prior to enrolment or previous intolerance of an SGLT2 inhibitor;
3. Participation in another study with investigational drug within the 30 days preceding and during the present study;
4. Type 1 diabetes mellitus;
5. Symptomatic hypotension or systolic blood pressure < 90 mmHg at 2 out of 3 measurements either at visit 1 or visit 2;
6. Coronary revascularization (percutaneous coronary intervention because of STEMI or coronary artery bypass grafting) or valvular repair/replacement within 12 weeks prior to enrolment or planned to undergo any of these operations after randomization;
7. Implantation of a CRT device within 12 weeks prior to enrolment or intent to implant a CRT device during 12 weeks of study observation period if indicated according to the actual guidelines [11];
8. Previous cardiac transplantation or implantation of a ventricular assistance device or similar device, or implantation expected after randomization;
9. HF due to restrictive cardiomyopathy, active myocarditis, constrictive pericarditis, hypertrophic obstructive cardiomyopathy or uncorrected primary valvular disease;
10. Symptomatic bradycardia or second or third degree heart block without a pacemaker;
11. Severe (eGFR <20 mL/min/1.73 m^2 by CKD-EPI), unstable or rapidly progressing renal disease;
12. Women who are pregnant or breast feeding;
13. Intention to become pregnant during the course of the study;
14. Known or suspected non-compliance, drug or alcohol abuse;
15. Inability to follow the procedures of the study, e.g. due to language problems, psychological disorders, dementia, etc. of the participant;
16. Patients with severely restricted liver function;
17. Patients with recurrent mycotic genital infections;

7.2 Recruitment and screening

Patients will be recruited from the inpatient and outpatient clinics of the University Hospital Zurich (USZ) and the Triemli Hospital Zurich. The Triemli Hospital is located in close proximity to the USZ and the hospital takes over heart failure patients and potential study candidates within the Zurich region. The USZ and the Triemli Hospital work together in a collaborative effort, however the Triemli Hospital will only inform the USZ study team about potential study candidates admitted to the hospital. The USZ study team will then contact the patients and inform about the DAPA-VOLVO trial. Of note, the Triemli Hospital does not serve as an additional study centre and all study relevant procedures (e.g. patient recruitment, patient screening, study visits etc.) will be performed only by the USZ study team at the USZ. Briefly, hospitalized/ ambulatory care patients will be asked if they are interested in participating in the study directly by the physician or a member of the study team. Interested participants will then be invited to the screening visit where the inclusion and exclusion criteria will be checked first. The screening assessments are described in section 9.3. Patients require laboratory results, which are assessed during the screening visit. Patients will receive no payment for study participation other than reimbursement for travel costs.

7.3 Assignment to study groups

Eligible participants will be randomized and assigned to the study groups as described in section 6.2.1.

7.4 Criteria for withdrawal / discontinuation of participants

The following circumstances will require withdrawal/discontinuation of a study participant:

- Withdrawal of informed consent;
- Investigator thinks that continuation would be detrimental to the patient's well-being;
- Pregnancy;

Study drug may be discontinued at the investigator's discretion if any of the following occurs:

- Any severe suspected drug-related AE;
- Any other protocol deviation that results in a significant risk to the patient's safety;

Patients that permanently interrupt study treatment should continue their recommended therapy.

Patients that are excluded from the study due to above reasons will be replaced.

Data and blood samples collected up to the time of withdrawal/discontinuation will be analyzed.

Data will be kept anonymously and all samples will be destroyed after publication of the study results.

The description of follow-up procedures is provided in section 9.2.5.

8. STUDY INTERVENTION

8.1 Identity of investigational products (treatment)

8.1.1 Experimental intervention (treatment)

Group A: Dapagliflozin (DAPA);

Generic name: Dapagliflozin propanediol;

ATC code: A10BK01, www.whocc.no;

Brand name: Forxiga ®;

Supplier and source: AstraZeneca AG;

Pharmaceutical form: Film-coated tablet;

Mode of administration: oral;

Color and appearance: Green, plain, and diamond shaped or yellow, biconvex, diamond shaped with number 10 debossed on 1 side and number 1428 on the other side;

Strength: 10mg/per tablet (Swissmedic number: 65176, www.swissmedicinfo.ch);

8.1.2 Control intervention (placebo treatment)

Group B: Placebo (PLA);

Supplier and source: AstraZeneca AG;

Pharmaceutical form: Film-coated tablet;

Mode of administration: oral;

Color and appearance: Look-alike placebo tablets are supplied in the same container/closure system. The placebo tablets contain lactose monohydrate, microcrystalline cellulose, magnesium stearate, and coating material (Opadry ® II).

8.1.3 Packaging, labelling and supply (re-supply)

The investigational medicinal product (IMP) dapagliflozin (10mg/tablet) and corresponding placebo (= study drugs) will be supplied by AstraZeneca AG in packaging of identical appearance in form of primary packed unlabelled bottles, containing 35 tablets per bottle.

The Sponsor is responsible for study specific labelling of study drugs. For the present study, the blinding and labelling of study drug bottles will be executed by the hospital pharmacy (Kantonsapotheke Zürich, KAZ).

The randomisation list will be generated via the secuTrial software and forwarded in numeric form (01-100) by the CTC to the KAZ.

The investigational treatment pack will be a zip-pack kit containing 3 labelled bottles per each patient (labelling and secondary packaging performed by KAZ). Each patient kit has a 2-part label. A unique medication number is printed on each part of this label which corresponds to one of the 2 treatment arms. Investigator staff will identify the investigational treatment package(s) to dispense to the patient by contacting the IRT and obtaining the medication number(s).

Immediately before dispensing the package to the patient, investigator staff will detach the outer part of the label from the packaging and affix it to the source document (drug label form) for that patient's unique subject number from 01 to 100.

Medication labels will be in the local language and comply with the legal requirements of each country. They will include storage conditions for the investigational treatment but no information about the patient except for the medication number.

Upon receipt of the study treatment supplies, an inventory must be performed and a drug receipt log filled out and signed by the person accepting the shipment. It is important that the designated study staff counts and verifies that the shipment contains all the items noted in the shipment inventory. Any damaged or unusable study drug in a given shipment (active drug or placebo) will be documented in the study files.

Shelf life of the study drugs is long enough to cover the entire study period. In case of early expiry or

loss, the study drugs may be resupplied by AstraZeneca AG.

8.1.4 Storage Conditions

Investigational treatment must be received by a designated person at the study site, handled and stored safely and properly, and kept in a secured location to which only the investigator and designees have access. Upon receipt, all investigational treatment should be stored according to the recommended storage conditions and the instructions specified on the labels.

The study product is to be stored below 30°C (range: 15°C - 30°C) and should be kept away from light and moisture. Clinical supplies are to be dispensed only in accordance with the protocol.

For the present study, the investigational treatment will be stored at the study site (Rämistrasse 100, room RAE E30) in a secured cupboard equipped with a temperature log system.

8.2 Administration of experimental and control interventions

8.2.1 Experimental and control intervention (placebo)

AstraZeneca AG will supply the investigators with all study medications (dapagliflozin, placebo) required for the course of the study. Patients will be provided with medication packs containing study drug corresponding to their assigned treatment arm (A= DAPA or B= PLA), sufficient to last until the next scheduled visit.

The treatment regimen of dapagliflozin (1 tablet/day, dose: 10 mg, route: orally) is based on major trials (e.g. DAPA-HF) showing beneficial effects in HF-patients while being well-tolerated by patients with this treatment setting.

Dapagliflozin can be principally taken at any time and independent of meals according to the professional drug information sheet [81].

Patients will be instructed to take their study drug doses (1 tablet per day per oral) regularly in the morning to ensure a consistent and comparable treatment setting. The study drugs should be taken with water, with or without food.

The first IMP dose will be taken after the baseline testing is completed (Table 1). The patients will be instructed to take each daily dose separated by ≥ 12 hours from the previous one. If the patient misses taking any study drug dose, he/she should take it as soon as possible, unless it is less than 12 hours until the following scheduled dose.

In this case of < 12 hours, the patient should skip the missed dose and return back to his/her regular study drug administration schedule.

All dosages prescribed and dispensed to the patient and all dose changes during the study must be recorded on the dosage administration record CRF.

8.3 Dose modifications

The following situations require modifications of the study drug or other medications as described below:

- Symptomatic hypotension;
- Hypovolemia;
- Hypoglycemia;

For patients who are unable to tolerate the protocol-specified dosing scheme interruptions of study treatment are permitted in order to keep the patient on study drug. The following guidelines should be followed:

- Every attempt should be made to maintain patients at the target study drug dose level throughout the trial. If the patient does not tolerate the target study drug dose level, the investigator can adjust or stop concomitant background medications for co-morbid conditions to rectify the situation, before considering down-titration to the next lower study drug dose

level.

- For hypotension or dizziness, consideration should be given to reduce the dose of concomitant antihypertensive agents and non-antihypertensive agents that lower BP, or the dose of diuretic can be reduced.
- For hypovolemia consideration should be given to reduce the dose of concomitant diuretic agents and non-diuretic agents that lower volume status.
- For hypoglycemia (e.g. T2DM) consideration should be given to reduce the dose of concomitant anti-diabetic agents (e.g. insulin) and non-anti-diabetic agents that lower glucose levels.

If despite adjustment of concomitant medications the situation is not rectified, the investigator may consider discontinuation of study treatment.

8.4 Compliance with study intervention

The investigator should promote compliance by instructing the patient to take the drug exactly as prescribed and by stating that compliance is necessary for the patient's safety and the validity of the study. The patient should be instructed to contact the investigator if he/she is unable for any reason to take the study drug as prescribed. Drug compliance will be tracked as described in section 8.8.

Non-adherence to study treatment will be defined as intake of less than 80% of the study drug dose as determined, using drug accountability logs.

Non-adherent patients will remain in the study and will be motivated to increase drug consumption and come to all study visits. Non-adherent patients will enter final data analysis in the intention-to-treat population but will not be analyzed in the per-protocol data set.

8.5 Data collection and follow-up for withdrawn participants

Patients that withdraw from the study drug will be encouraged to participate in all regular study visits and assessments. Patients that withdraw consent will be included in the intention-to-treat analysis with the measurements that have been obtained until the withdrawal.

8.6 Trial specific preventive measures

As stated, the study treatment will be taken on top of recommended therapy in heart failure. The trial specific preventive measures are adapted partially from the DAPA-HF trial [5] where the efficacy and safety were shown for both study arms in heart failure patients.

Dapagliflozin or placebo will be discontinued in case of:

- Pregnancy;
- Diabetic ketoacidosis (DKA);
- Fournier gangrene (FG);
- Severe recurrent genital infections (GI) and urinary tract infections(UTI);

A temporary discontinuation is permitted in case of:

- An acute, unexpected decline in the eGFR;
- Volume depletion, or hypotension (or to avoid these conditions);
- Hypoglycemia;

A subsequent restarting of treatment is intended, if possible.

The use of these trial specific preventive measures will be recorded in the eCRF.

8.7 Concomitant interventions (treatments)

The investigator should instruct the patient to notify the study site about any new medications he/she takes after the patient was enrolled into the study. All medications, procedures and significant non-drug therapies (including physical therapy and blood transfusions) administered after the patient was enrolled into the study must be recorded in the eCRF.

There are no specific or relevant concomitant care and interventions that are permitted (additional treatments) during the trial.

8.8 Study drug accountability

Investigational product supplies, which will be provided to the Sponsor-Investigator, must be kept in a secure, limited access storage area under the recommended storage conditions. The investigator must maintain accurate and adequate records including dates, lot number, quantities received and individual usage.

The study drug reconciliation will be performed at the study end to document drug assigned; drug consumed, and drug remaining. This reconciliation will be logged on the drug accountability form, and signed and dated by the study team.

Patients will be asked to return all unused investigational treatment and packaging at the end of the study or at the time of discontinuation of investigational treatment.

At the conclusion of the study, and as appropriate during the course of the study, the investigator will return all unused investigational treatment, packaging, drug labels, and a copy of the completed drug accountability log to the monitor.

8.9 Return or destruction of study drug

At the completion of the study, there will be a final reconciliation of drug shipped, drug consumed, and drug remaining. This reconciliation will be logged on the drug accountability form, signed and dated. Any discrepancies noted will be investigated, resolved, and documented prior to destruction of unused study drug on site. Drugs destroyed on site will be documented in the study files.

9. STUDY ASSESSMENTS

9.1 Study flow chart and table of study procedures and assessments

The study flow chart and table of procedures and assessments are depicted in the summary part of the protocol.

9.2 Assessments of outcomes

9.2.1 Assessment of primary outcome

The primary study outcome is the change in relative plasma volume status (ΔPVS) from baseline to 12 weeks of dapagliflozin treatment on top of recommended therapy in comparison to placebo.

9.2.1.1 Assessment of relative plasma volume status

The relative plasma volume status (PVS) has been proposed to have prognostic implications in heart failure patients and patients with cardiovascular diseases (see also 3.1.2). The PVS is defined as the % deviation of actual plasma volume (aPV) from the sex-dependent ideal plasma volume (iPV) as calculated by use of the Hakim-formula [36]:

1) PVS = [(aPV – iPV) / iPV)] x 100%;

The ***actual plasma volume*** (aPV) can be either estimated (ePV) by use of the following sex-

dependent formula or measured (mPV):

2) aPV = (1 – hematocrit) x [a + (b x weight in kg)], a = 1530 in males, a = 864 in females and b = 41 in males, b = 47.9 in females;

The ***ideal plasma volume*** (iPV) can be calculated with the following sex-dependent simple formula:

3) iPV = c x body weight (kg), constant c = 39 in males and c = 40 in females;

In the present study, the PVS will be calculated using the optimized carbon monoxide rebreathing (OpCO) technique to measure the actual plasma volume (aPV = mPV).

9.2.1.2 Assessment of relative plasma volume status by use of the optimized carbon monoxide rebreathing technique

As stated, the relative plasma volume status will be calculated using the OpCO technique to measure the actual plasma volume (mPV) in heart failure patients by use of the Hakim formula as described previously (see 9.2.1.1):

PVS = [(mPV – iPV) / iPV)] x 100%;

The OpCO technique is not yet established in clinical routine, however it is highly accurate and reliable method to measure total haemoglobin mass (Hb_{mass}), the basis for calculation of intravascular volumes, e.g. plasma volume.

This is reflected by a reported low typical error (TE, 1-2%) and coefficient of variation (CV, 2.2%) for measuring Hb_{mass} [39-41].

Importantly, the OpCO technique has been recently shown to be safe and well-tolerated, and feasible to apply in heart failure patients with severe (LVEF< 30%) left ventricular dysfunction [42] but in contrast to radioactive isotope labelling techniques this technique does not expose patients to radiation.

Recent applications of the optimized CO-rebreathing technique to measure blood- and plasma volumes in patients with chronic kidney disease [43], dialysis-dependent kidney failure [44, 45] and patients with chronic liver disease [46] further emphasizes the brought ranged potential of this technique in the clinics.

A direct comparison between the measured plasma volume (mPV) assessed by OpCO technique and the estimated PV (ePV) assessed by the Hakim formula [36], which was used by others [10, 37, 93], has not yet been performed.

However, a recent study by Martens et al. [10] could demonstrate that the estimated PV in HF patients by use of a radioactive isotope (technetium-99) red blood cell (RBC) labelling technique correlated well with the estimated PV as calculated by the Hakim formula. In this context, Gore et al. [39] could show in a meta-analysis that the measurement error of CO-rebreathing is comparable (even lower) to radioactive RBC labelling technique.

Thus, the measured PV by use of OpCO technique (see formulas below) should reflect well the estimated PV based on the Hakim formula. Actually, it may reflect ‘true’ PV levels more accurately as it’s also based on the direct measurement of volume independent total haemoglobin mass (Hb_{mass}, see formula below) and not solely on concentration dependent measures (e.g. Hct). Nevertheless, we will compare (correlate) the 2 different PV estimations with each other.

The underlying formulas used by us [45] and others [44] to calculate blood volume using OpCO technique are as follows:

1) $nCO_{absorbed} = P_{atm} \times VCO_{absorbed} / (R \times T)$;

2) $nHb_{tagged} = nCO_{absorbed} / 4$; (1 mole Hb binds 4 moles CO);

3) $nHb_{total} = (nHb_{tagged} / \Delta HbCO) \times 100\%$;

4) $Hb_{mass} = nHb_{total}$ x 6.44 x 104 g/mol (= molar mass of Hb);

5) RBCV = Hb_{mass} x Hct / Hb;

6) BV = RBCV / Hct;

7) PV (mPV) = BV – RBCV;

*Hct should be corrected to whole-body Hct by the factor 0.91 [94].

Abbreviations: $nCO_{absorbed}$: amount of absorbed CO in moles after CO-rebreathing, P_{atm}: atmospheric pressure, $VCO_{absorbed}$: absorbed volume of CO in liters, R: gas constant (0.08206 l atm / (mol·K), T: room temperature in Kelvin, nHb_{total}: total amount of Hb in moles, nHb_{tagged}: amount of tagged Hb in moles, ΔHbCO (%): difference in carboxyhemoglobin (HbCO) measured in venous blood samples pre- and post CO-rebreathing, Hb_{mass} (g): total hemoglobin mass, RBCV (l): red blood cell volume, BV: blood volume, mPV: measured plasma volume;

9.2.1.3 Assessment of blood volume by use of the optimized carbon monoxide rebreathing technique

The blood volume (BV) will be determined by applying OpCO technique using a fully automated device (Detalo Instruments, Denmark) as illustrated (Fig. 10) with a very low typical error (≤ 1%).

Briefly, the participant will rest for at least 10 minutes in a supine position (resting phase, RP) prior to the start of the BV-measurements.

During this period a venous catheter (20G venflon, BD, USA) will be inserted into an antecubital vein and a first blood sample (2ml) will be taken shortly before the start of the rebreathing test to measure the percent carboxy hemoglobin (HbCO), the hemoglobin concentration (Hb), oxygen saturation (SaO2) and hematocrit (Hct) with a hemoximeter (ABL800, Radiometer, Denmark).

At the beginning of the rebreathing manoeuver, the patient will breathe 100% of oxygen for 1 (flushing period, FP) through a mouth piece (mask), which is connected via a breathing filter and tubing system to the automated rebreathing device.

Thereafter, a bolus of 1 ml/kg of chemically pure carbon monoxide (CO, 99.997%, CO N47, Air Liquide, France) will be administered into the closed breathing circuit and the resulting gas mixture will be rebreathed for at least 2 to max. 10 minutes (rebreathing period, RBP) before patient will be disconnected from the rebreathing circuit.

The second venous blood sample will be taken from the same venous catheter 10 minutes after the start of the RBP to measure again the aforementioned parameters. The blood pressure and heart rate of the study participant will be assessed noninvasively before and after the BV-measurements by use of a non-invasive hemodynamic monitoring device (Finometer Midi, FMS, The Netherlands) or comparable NIBPD.

Duration of intervention: 20 min;

Main outcome parameters (units): PVS (%), Hb_{mass} (g), BV (ml), RBCV (ml), PV (ml), BV_{ratios} ();

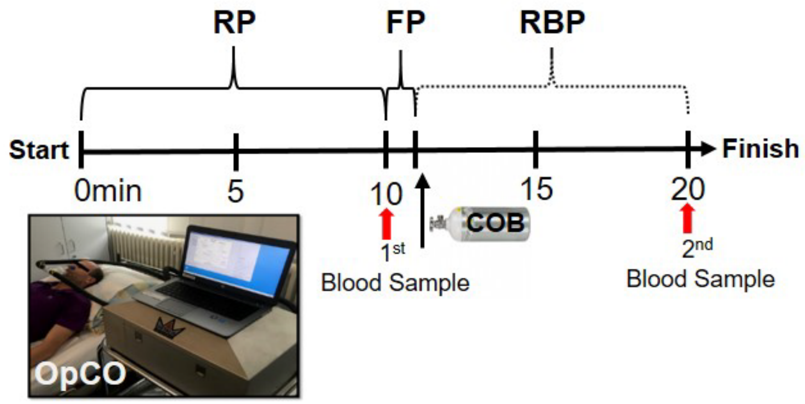

Fig. 11: Blood volume assessment using fully-automated optimized carbon monoxide rebreathing (OpCO) technique. A venous catheter will be placed during the 10-min resting phase (RP) in supine position and a 1st venous blood sample will be taken shortly prior to the rebreathing measurement. After connecting the patients to the rebreathing circuit via mouth piece (mask) the BV assessment will start with a 1-min flushing period (FP), followed by a CO-bolus injection (COB) based on subjects body weight, which will be rebreathed for at least 2 minutes to maximal 10 minutes (rebreathing phase, RBP). The 2nd venous blood sample will be taken 10 minutes after the beginning of the rebreathing measurement.

9.2.2 Assessment of secondary outcomes

9.2.2.1 Micro-vascular endothelial function via retinal vessel analysis

Static and dynamic retinal vessel analysis (RVA) will be assessed as a measure of microvascular endothelial function (Fig. 12). The RVA is conducted using an Imedos dynamic retinal vessel analyzer (Imedos, Jena, Germany). This device uses a Zeiss FF450 plus fundus camera (Carl Zeiss Meditec AG, Jena, Germany) connected with two charge-coupled device (CCD) cameras (Imedos, Jena, Germany) for image acquisition and a customized computer software system for image analysis (Imedos, Jena, Germany). The RVA will be conducted using our established protocols [95].

Briefly, one eye will be randomly selected. Mydriasis will be induced with 0.5% tropicamide eye drops (1-2 drops). After a waiting time of 10 minutes to achieve sufficient mydriasis, blood pressure will be measured (sitting position) and the patient positioned in front of the fundus camera. The analysis starts with retina in focus and the upper temporal fundus visualized. Then, a 1.0-1.5mm long segment of an artery and a vein will be selected using the RVA software.

First, the dynamic retinal vessel analysis will be performed (DVA, Fig. 12A). Therefore, the flicker-light response of 3 consecutive 20s-flicker-periods will be measured and the flicker-light induced dilatation of a selected retinal artery (FID_a, red arrow A1) and vein (FID_v, blue arrow V1) will be evaluated.

Thereafter, the static vessel analysis (SVA) will be performed by taking fundus pictures of the retina and measure the diameters of the retinal arterioles (central retinal artery equivalent, CRAE) and venules (central retinal vein equivalent, CRVE) in standardized concentric segments (Fig. 12B) and calculate the arterio-venule-ratio (AVR).

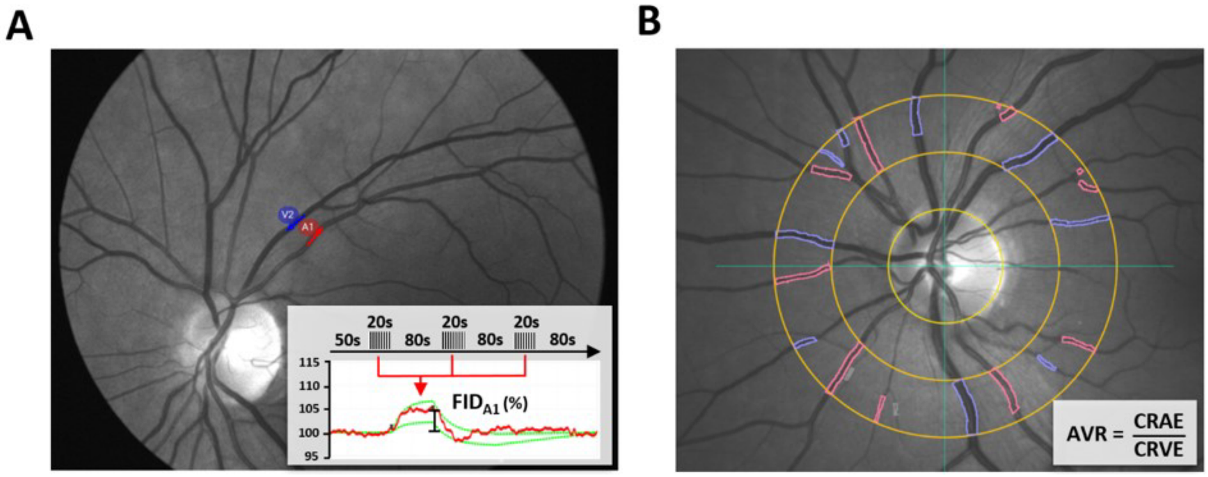

Fig. 12: Retinal vessel analysis (RVA) consists of a dynamic measurement (DVA) of flicker-induced dilatation (FID) of retinal arterioles (FIDa) and venules (FIDv) using a standardized flicker-protocol (A) and a static measurement (SVA) of arteriolar and venular diameters (B) to calculate the arterio-venule-ratio (AVR).

<u>Duration of study intervention:</u> 30 min

<u>Main outcome parameters (units):</u> FID_a (%), FID_v (%), CRAE (µm), CRVE (µm), AVR ();

9.2.2.2 *<u>Macro-vascular endothelial function via flow mediated vasodilatation</u>*

To non-invasively evaluate endothelial function of larger arterial vessels, the flow-mediated vasodilatation (FMD) will be measured using established protocols [96]. Vasodilation of the brachial artery is assessed by a high-resolution ultrasound vessel wall tracking device with a 10 MHz linear array transducer (Siemens Acuson X300, Siemens AG).

The measurement will always be performed in the morning in the fasted state after abstaining from nicotine and coffee for at least 24 hours. This method makes use of the property of endothelium to release NO in response to shear stress from increased flow after compression with a blood pressure cuff.

Briefly, while lying in the supine position, the brachial artery of the upper arm is visualized longitudinally with the ultrasound probe, giving care to achieve a sharp and continuous near and distal wall. Then, the arterial diameter will be measured continuously for 10 minutes in the brachial artery (FMD-Studio, Pisa, Italy). Measurement consists of a 1 minute baseline, a 5 minute occlusion and a 4 minute measurement and recovery period.

After a one minute baseline measurement, a blood pressure cuff is inflated 50 mmHg above systolic pressure at the lower arm for 5 minutes. Then the blood pressure cuff is released and hyperemia occurs. The FMD outcome is plotted as the percent increase in diameter from the baseline period mean diameter to the maximum diameter after cuff release.

For validation purposes of the measurement, the endothelial independent effect will be measured using pharmacological dilatation of the brachial artery after glycerol trinitrate (GTN). One spray of sublingual GTN is given (0.4 mg sublingual dose, nitrolingual spray, Pohl-Boskamp, Germany) and the brachial artery diameter and blood flow will be recorded.

GTN will not be given if there is a high risk of symptomatic hypotension by decision of the investigator. Inflation may lead to transient numbness of the hand and fingers. Of note, nitroglycerin can cause transient tension-headache and dizziness in about 3% of the patients. The reproducibility of our laboratory was already published [97].

<u>Duration of study intervention:</u> 40 min;

<u>Main outcome parameters (units):</u> FMD (%), GTN (%);

9.2.2.3 *Arterial stiffness via pulse wave analysis*

Arterial stiffness will be assessed non-invasively by planar tonometry technique to evaluate pulse wave velocity (PWV) and pulse wave analysis (PWA) by use of the SphygmoCor® system (AtCor Medical, Germany) [98]. The PWA will be evaluated at the level of the radial artery. The relevant outcome parameter is augmentation index normalized to a heart rate of 75 beats per minute (Aix@75). The PWV will be measured between the carotid and femoral artery according to the recent guidelines for assessment of arterial compliance [99, 100].

Duration of study intervention: 15 min;

Main outcome parameters (units): PWV (m/s), Aix@75 (%);

9.2.2.4 *Blood volume profile and body water distribution*

The blood volume profile will be assessed via the OpCO technique as previously described in detail in the primary outcome section (see 9.2.1). The body fluid volume and distribution will be measured non-invasively via the bioimpedance analysis (BIA) technique using InBody S10 device (InBody, Japan Inc., Tokyo, Japan) with 8 tactile electrodes as previously described by others [63, 64].

Briefly, the BIA will be performed according to the manufactures instructions in supine position after resting for 10 minutes prior to the measurement.

Duration of study intervention (BIA): 15 min;

Main outcome parameters for BIA (units): TBW (l), ICW (l), ECW (l), ICW/TBW ratio (), ECW/TBW ratio ();

9.2.2.5 *Health-related quality of life in heart failure via Kansas City cardiomyopathy questionnaire*

The health-related quality of life (HRQoL) will be assessed by use of the Kansas City cardiomyopathy questionnaire (KCCQ), which is self-administered 23-item questionnaire developed to provide a better description of HRQoL in patients with chronic heart failure. The KCCQ was previously proven to be valide, reliable and responsive [101].

Duration of study intervention: 5 minutes;

Main outcome measures: KCCQ overall scores and sub-scores ();

9.2.2.6 *Sleep quality in heart failure via Pittsburgh sleep quality index questionnaire*

The sleep quality of the study patients will be assessed via the well-established and validated Pittsburgh sleep quality index (PSQI) questionnaire [102], which comprises 10-items and is self-administered.

Duration of study intervention: 5 minutes;

Main outcome measures: PSQI overall scores and sub-scores ();

9.2.3 Assessment of other outcomes of interest

9.2.3.1 *Cardiorespiratory fitness via cardiopulmonary exercise testing*

Cardiopulmonary exercise testing (CPET) is an important and in the clinical routine frequently used method to estimate the cardiorespiratory fitness (CRF) in patients. The golden standard to estimate the CRF of a patient is the measurement of the maximal or peak oxygen uptake ($VO2_{max}$, $VO2_{peak}$), which are quantitative predictors of all-cause mortality and cardiovascular events in healthy subjects but also in heart failure patients and patients with other cardiovascular diseases [103, 104]. Briefly, the CPET test protocol is a stepwise graded exercise protocol, which will be performed either on a

treadmill or cycle ergometer until voluntary exhaustion or symptom-limited stop. The applied exercise test protocol follows the recommended guidelines for clinical CPET [105, 106]. Additionally, a 6-min walk test (6MWT) will be performed, which was shown to be a reliable and valid clinical exercise test in patients with chronic heart failure [107].

Duration of study intervention: CPET: 30 min, 6MWT: 15 min;

Main outcome parameters (units): CPET: VO_{2peak} (ml/min), $VEVCO_{2slope}$ (), 6MWT: 6MWD (m);

9.2.3.2 *Morphological and functional cardiac assessment via transthoracic echocardiography*

The transthoracic echocardiography (TTE) a non-invasive imaging technique and is frequently applied in clinical routine to assess the actual status of cardiac function (e.g. ventricular ejection fraction, EF). The TTE will be performed following actual standards and guidelines.

Duration of study intervention: 40 minutes;

Main outcome parameters: LVEF (%), LVM (g), LVMI (g/m^2), RWT (), PASP (mmHg), strain analysis parameters;

9.2.3.3 *Sympathetic outflow assessment via muscle sympathetic nerve activity*

An increased sympathetic drive is a hallmark of congestive heart failure but is generally frequently seen in heart failure patients and contributes to the pathophysiology of this condition [108].

The muscle sympathetic nerve activity (MSNA) will be assessed via minimally invasive microneurography technique according to actual guidelines [109]. Multi-fibre recordings of MSNA will be obtained from the peroneal nerve posterior to the fibular head with tungsten microelectrodes (200-µm shaft diameter, 1 to 5 µm uninsulated tip, Medical Instruments, University of Iowa).

Briefly, the study participant will be placed in a supine position. A reference electrode will be inserted subcutaneously 1 to 2 cm from the recording electrode. The recording electrodes are connected to a low-noise, high-gain amplifier NeuroAmp Ex (ADInstruments, Oxford, UK).

The MSNA and surface ECG are continuously recorded with the PowerLab 8/35 hardware connected to a laptop computer using the LabChart Pro software (Vers.8, ADInstruments, Oxford, UK). Signal processing will be performed with LabChart Pro software. MSNA is quantified in a computer-assisted evaluation of the frequency and the amplitude of the sympathetic bursts.

Cold pressor test (CPT):

The MSNA will be assessed at baseline and after stimulation via the well-established and also well-tolerated ‘cold pressor’ test during which the study patients hand will be immersed into an ice water bath for 2 minutes [110, 111].

Duration of study intervention: 60 minutes;

Main outcome parameters: burst frequency (bursts/min), burst incidence (bursts/100 heart beats), total activity as AUC ();

9.2.3.4 *Tissue sodium content assessment via sodium (^{23}Na)-magnet resonance imaging*

Salt- and water homeostasis are strongly linked to each other. Sodium is stored in the skin and muscle tissue bound to glycosaminoglycans, which act as sodium-buffers and subsequently affect the extracellular fluid compartment by water retention [28].

In heart failure patients, the dysbalance of sodium- and body fluid homeostasis seems to be the driving force of congestion, the most common reason for hospital admission in heart failure patients [112].

The tissue sodium content in calf skin, muscle, and bone tissue will be quantified using the non-invasive sodium (^{23}Na) magnetic resonance imaging (Na-MRI) technique, which was shown to have a high degree of reliability between scan and rescan tests by use of linear regression analysis [113].

A recent work could show by use of Na-MRI accumulation of Na^+ in muscle and skin in patients with acute heart failure in comparison to healthy subjects. Furthermore, the authors reported that diuretic

treatment with furosemide can at least partly mobilize Na^+-deposition.

Briefly, the study participant will placed in supine position into the MRI. The measurements will be performed at 3T using a dual-tunable ^{23}Na/1H coil as described previously [114] and by using a standardized test protocol. The Na-MRI is a non-invasive method and thus should not cause any harm to the patients.

In addition to ^{23}Na-MRI, the renal perfusion and renal oxygenation can be assessed without the use of contrast agents via arterial spin labelling MRI (ASL ^{1}H-MRI) [115] and blood oxygenation level dependent effect MRI (BOLD MRI) [116] as physiological parameters of kidney function.

Duration of study intervention: 60 to 120 minutes;

Main outcome parameters: tissue Na-content in different tissues (e.g. skin, muscle), renal perfusion, renal oxygen consumption;

9.2.3.5 Laboratory assessments and analyses

Peripheral venous blood samples (total sample blood volume/visit: 50ml) will be taken according to current medical standards during the screening visit (V1), baseline visit (V2) and during the follow up visits (V3-V6).

Advanced laboratory analysis (including most of the variables listed below) will only be performed at V2 (baseline visit) and V5 (final visit).

During V3-V4 (follow-up visits) primarily routine blood parameters (e.g. RBC count, Hb, Hct, etc.) and laboratory safety variables (e.g. eGFR, NT-proBNP) will be measured.

For the screening visit (V1) primarily laboratory parameters (e.g. eGFR) will be measured, which are necessary to check for defined in-, exclusion criteria (see 7.1).

Of note, during visits where CPET (V2/V4) and/or OpCO will be performed blood samples will be taken pre- and post test intervention.

The laboratory assessments will be performed in the fasted state (minimum of 6 hours without food; patients are allowed to drink water, but no coffee or tea) for hematology and clinical chemistry. The collected samples will be analysed by the Department of Clinical Chemistry and Haematology, University Hospital Zurich.

For specific sample analysis (blood/urine), which are not provided by the USZ internal laboratories and where samples cannot be stored at -80°C, e.g. for active exosome analysis [117] they will be immediately transported on ice to the external laboratories (Institute of Human Physiology/ Center for Molecular Cardiology, University of Zurich, UZH) for further processing.

For specific samples analysis (blood/urine), which are not provided by the USZ internal laboratories (e.g. FGF23) frozen plasma samples will be generated, frozen and stored to be later transported in cooled (dry ice) and insulated transport boxes to the external laboratories.

Blood samples and urine samples for later analysis will be collected in (BD Vacutainer, BD-Plymouth, UK) and immediately cooled and blood samples will be centrifuged to obtain the blood plasma. Thereafter, vials will be aliquoted in endotoxin-free tubes, frozen and stored at -80°C.

The samples will be kept frozen until analysis will be performed (including during shipment). The analysis of special plasma markers such as N-terminal pro brain natriuretic peptide (NT-pro BNP) will be conducted using commercially available ELISA kit technology.

Laboratory blood parameters (abbreviation, unit):

Hematology: Red blood cell count (RBC count, T/l), platelet count (G/l), leukocyte count (G/l), hemoglobin (Hb, g/l), hematocrit (Hct, ratio), mean corpuscular volume (MCV, fl), mean corpuscular hemoglobin (MCH, pg), mean corpuscular hemoglobin concentration (MCHC, g/l), red cell distribution width (RDW, %);

Biochemistry (abbreviation, unit): Sodium (Na^+, mmol/l), potassium (K^+, mmol/l), calcium (Ca^{2+}, mmol/l), phosphate (mmol/l), creatinine (µmol/l), glomerular filtration rate (eGFR, ml/min/1.73m^2), blood urea nitrogen (BUN, mg/dl), alanine transaminase (ALT, U/l), alcalic phosphatase (AP, U/l), glucose (mmol/l), lactate (LA, mmol/l), creatine kinase (CK, U/l), total cholesterol (mmol/l), low density

lipid protein (LDL, mmol/l), high density lipid protein (HDL, mmol/l), triglyceride (mmol/l), high-sensitivity troponin T (hsTnT, ng/l), natriuretic peptide (NT-proBNP, ng/l), thyroid-stimulating hormone (TSH, mU/l), cortisol (mmol/l), parathyroide hormone (PTH, ng/l), trimethylaminoxide (TMAO, µmol/l), erythropoietin (EPO, IU/l)), aldosterone (ng/l), renin (mU/l), copeptin/ C-terminal pro arginine vasopressin (CT-proAVP, pmol/l), fibroblast growth factor 23 (intact: iFGF23, c-terminal: cFGF23, pg/ml), Klotho (pg/ml), serum iron (µmol/l), serum ferritin (µg/l), transferrin saturation (TSAT, %), hepcidin (ng/ml), myeloperoxidase, (MPO, pmol/l), C-reactive protein (CRP, mg/l), interleukine 6 (IL-6, pg/ml).

Urine: Albumin (mg/mmol creatinine), creatinine (mmol/l), phosphate (mmol/l)
, calcium (mmol/l), cFGF23 (RU/ml);

9.2.4 Assessment of safety outcomes

9.2.4.1 Adverse events

Serious adverse events will be routinely assessed and recorded on main study visits (V2 to V5). The definition and way of such events to be assessed and reported is defined in section 10. On each visit V2-V5, standardized questions on the occurrence of any adverse events will be conducted.

Spontaneous events will also be recorded in the eCRF and reported as defined in section 10.

9.2.4.2 Laboratory parameters

Prespecified safety laboratory parameters are eGFR and NT-proBNP.

They will be assessed on each visit (V1-5) of the main study and sampled as described in section 9.2.3.

The following parameter is to be documented as an adverse event:

eGFR < 20 mL/min/1.73m^2 (CKD-EPI formula) or eGFR decrease > 50% of baseline;

Any aberration in other laboratory parameters that are assessed as described in section 9.2.3 and are regarded clinical significant by the investigator are to be documented as an adverse event.

9.2.4.3 Vital signs

Blood pressure, heart rate and peripheral oxygen saturation will be manually assessed in a sitting position after 5 minutes of resting.

9.2.5 Assessments in participants who prematurely stop the study

Participants who prematurely withdraw from the study will be invited for a final study visit where a medical examination and blood sampling will be performed and the incidence of adverse events, the eGFR, the blood pressure, and the cardiac biomarker (NT-proBNP) will be assessed and patients transitioned back to standard recommended therapy.

9.3 Procedures at each visit

9.3.1 Visit 1 (V1, Screening visit)

The study information (SI) will be provided to the potential study candidate at least 24 hours prior to enrolment (Fig.1).

The screening visit (day: -5d to -1d, duration 30 minutes) will be used to determine eligibility of the study participant. After receiving and signing the informed consent the patient will be tested against the inclusion and exclusion criteria. For this process, a complete medical history and a standard physical examination (incl. auscultation of the heart and lungs), laboratory parameters (screening) and

vital signs will be determined as described under section 9.2. Additionally, a pregnancy test will be performed in premenopausal (or postmenopausal < 2 years) female patients with childbearing potential and will be introduced orally and in written form (study information sheet, PIC) to use reliable forms of contraception throughout the study period and 3 months thereafter. The eligible patients will be enrolled into study and randomly assigned to either dapagliflozin (DAPA) or placebo (PLA) group by use of the secuTrial software system as described in section 6.2.1.

Of note, visit 1 will be integrated into visit 2 whenever feasible.

Of the in total 80 study patients to be enrolled into the main study those patients who are willing to voluntary participate in the optional advanced study program will perform additional tests as described in section 9.3.3.

9.3.2 Visit 2/5 (V2/V5, Baseline visit/Final visit)

The baseline/final visit (V2/V5, time points: -2d to -1d/12w ± 1w) the main test session (duration 240 min) will start in the morning and in fasted state. The schedule of planed study assessments and interventions are illustrated in Fig. 13. At V5 a specific focus will be on the assessment of AEs and safety measures since it is the last main study visit.

Main Session: First, a focused clinical anamnesis and standard physical examination (incl. auscultation of the heart and lungs) will be conducted including vital signs (e.g. blood pressure, heart rate) will be measured before blood urine samples will be collected. After filling out the clinical questionnaires (e.g. KCCQ) and the scheduling of the study visits the patient will receive a tropicamide eye drop in one eye.

Then, the measurements as described previously (see 9.2) will be start beginning with the evaluation of arterial stiffness (AS) via planar tonometry of the radial artery (PWA) and the assessment of the carotid to femoral pulse wave velocity (PWV), followed by the evaluation of microvascular function via retinal vessel analysis (RVA).

The RVA starts with the dynamic part (DVA), the flicker-light induced vasodilatation of retinal arterioles (FID_a) and venules (FID_v) and continuous after a short recovery brake with the static vessel analysis (SVA) by taking pictures of the retina with the fundus camera to estimate the arterio-venular ratio (AVR). Afterwards, the brachial-artery ultrasound with measurement of the flow-mediated (vessel) dilatation (FMD) and the dilatation-response to glyceryl trinitrate (GTN) will be conducted while the patient is continuously placed in a supine position. The GTN will only be performed when the a priori measured systolic blood pressure value is ≥ 100 mmHg.

Thereafter, the patients will rest for 15 minutes in supine position before the start of the final measurement block. During this resting phase the electrodes for the BIA to estimate body water content (TBW) and distribution (ICW, ECW) will be placed and a venous catheter will be inserted into one of the patients arm veins.

In the final part, the blood volume (BV) and BV-related parameters (PV, RBCV, Hb_{mass}) will be measured by use of the optimized CO-rebreathing (OpCO) technique.

Finally, the patients will be dispensed with the study drug and instructions on how to take it (V2).

9.3.3 Visit 2a/5a

As stated in 6.1.1 the advanced session(s) (duration: 240 min) will be performed either on the same day (afternoon after 1 hour recovery brake) or in close as possible/ feasible proximity to V2 (V2a)/V5 (V5a) and prior to treatment start. In dependence of the logistical feasibility and testing capacity, the testing will continue in the voluntary study participants with a sympathetic nerve activity test (MSNA), a transthoracic echocardiography (TTE), a sodium magnetic resonance imaging (Na-MRI) scan and finally a standardized cardiopulmonary exercise test (CPET) and 6-minute walk test (6MWT) (see 9.2).

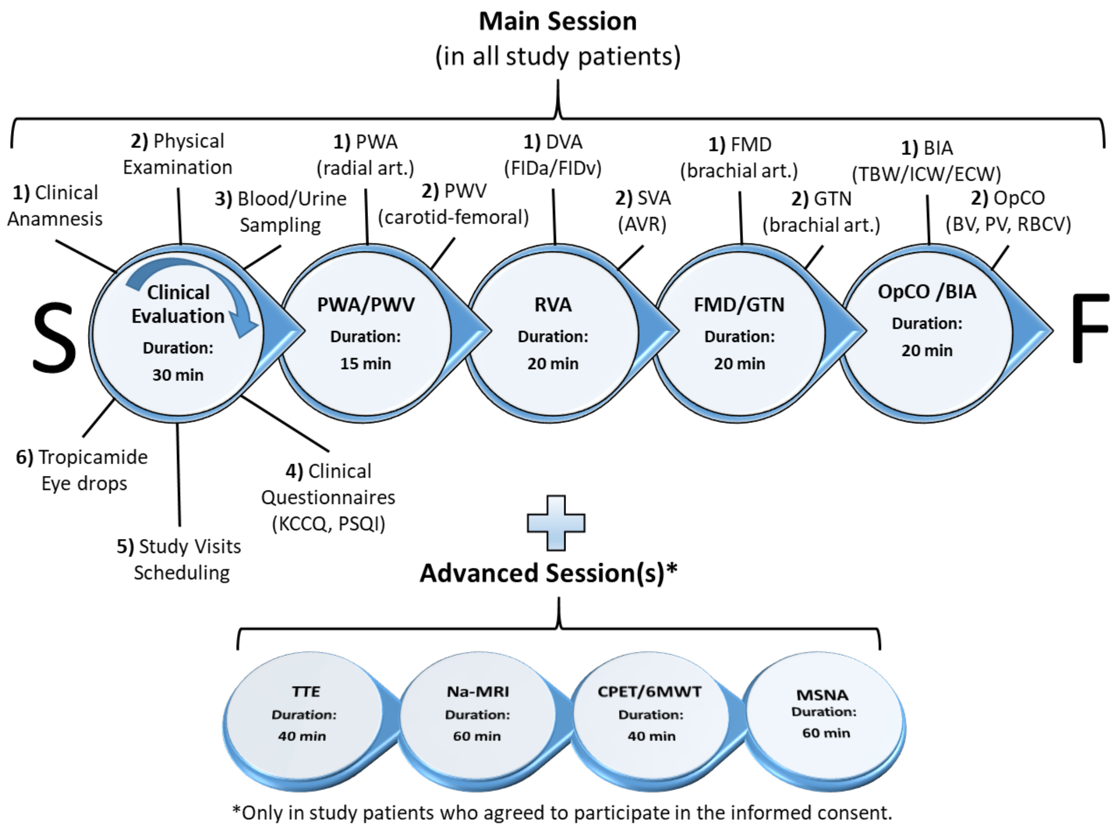

Fig. 13: Visit 2/5 (Baseline/Final visit, time: -2d to -1d/6w ± 5d): A graphical overview.

The main study session will be performed in the morning by all enrolled study participants. Patient who voluntary agreed to participate in the advanced session(s) program will additionally perform the advanced study tests either at the same day (afternoon, after 1 hour recovery brake) or in close proximity to V2/5 (V2a/V5a). Abbreviations: KCCQ: Kansas City cardiomyopathy questionnaire, PSQI: Pittsburgh sleep quality index, PWA: pulse wave analysis, PWV: pulse wave velocity, RVA: Retinal vessel analysis, DVA: Dynamic vessel analysis, FIDa: Flicker-light-induced arterial dilatation, FIDv: Flicker-light-induced venous dilatation, SVA: Static vessel analysis, FMD: Flow-mediated dilatation, GTN: Glyceryl trinitrate test, BIA: Bioimpedance analysis, TBW: Total body water, ICW: Intracellular water, ECW: Extracellular water, DAPA: Dapagliflozin-treatment group, PLA: Placebo-treatment group, MSNA: Muscle sympathetic nerve activity, BV: Blood volume, PV: Plasma volume, RBCV: Red blood cell volume, Hb_{mass}: Total haemoglobin mass, TTE: Transthoracic echocardiography, Na-MRI: Sodium magnet resonance imaging, CPET: Cardiopulmonary exercise testing, 6MWT: 6-minute walk test.

9.3.4 Visit 3/4 (V3/4, Follow-up visit 1/2)

The first (V3, time point: 2w, duration: 30 minutes) and second follow-up visit (V4, time point: 6w, duration: 30 minutes) after treatment start will include a clinical evaluation as previously described (see 9.3.2), however without administration tropicamide eye drops. The OpCO measurement will be performed to measure the PVS, the primary outcome variable as well as the BIA to measure TBW and its distribution (ICW/ECW). Furthermore, a specific focus will be on the assessment of potential AEs and defined safety measurements will be assessed as described in section 9.2.4.2.

9.3.5 Visit 6 (V6, Follow-up visit of prospective observational study)

The follow-up study visit (V6, time point: 12m post IMP treatment end, duration 240 minutes) of the prospective observational study will include a clinical evaluation as previously described (see 9.3.2). The testing will include the blood volume and body water assessment via OpCO and BIA, arterial stiffness (AS) via planar tonometry, endothelial function via FMD and microvascular function via RVA.

Of note, the follow-up visit V6 is part of the prospective observational study DAPA-VOLVO-Follow-U and following the end of the main study (V5) and will be evaluated and analysed independent from the main study (V2-V5).

10. SAFETY

10.1 Drug studies

The Sponsor's SOPs provide more detail on safety reporting. The IMP Dapagliflozin has Swiss marketing authorisation and it has been shown in clinical trials to be a safe and well-tolerated drug in patients with heart failure (see also 3.7).

During the entire duration of the main study (V2-V5), all clinically relevant adverse findings, potentially IMP-related (see also 10.1.1, Table 4 Causality: 'Possibly') adverse events (AE) and all serious adverse events (SAEs) are collected, fully investigated and documented in source documents and case report forms (CRF). Study duration of the main study encompasses the time from when the participant signs the informed consent until the last protocol-specific procedure of the main study has been completed.

Of note, visit V6 is an optional follow-up visit 12 months after the final visit V5 of the main study (V2-V5) and part of the prospective observational study DAPA-VOLVO-Follow-U and will be independently evaluated and analysed (see also Fig.1, 5.4, 6.1.1, 10.1, 10.1.3).

During the time period between the end of the main study (final visit: V5) and the optional visit V6 without interventions AEs will not be recorded.

10.1.1 Definition and assessment of (serious) adverse events and other safety related events

An **Adverse Event (AE)** is any untoward medical occurrence in a patient or a clinical investigation participant administered a pharmaceutical product and which does not necessarily have a causal relationship with the study procedure. An AE can therefore be any unfavourable and unintended sign (including an abnormal laboratory finding), symptom, or disease temporally associated with the use of a medicinal (investigational) product, whether or not related to the medicinal (investigational) product. [ICH E6 1.2]

A **Serious Adverse Event (SAE)** is classified as any untoward medical occurrence that:

- results in death,
- is life-threatening,
- requires in-patient hospitalization or prolongation of existing hospitalisation,
- results in persistent or significant disability/incapacity, or
- is a congenital anomaly/birth defect.

In addition, important medical events that may not be immediately life-threatening or result in death, or require hospitalisation, but may jeopardise the patient or may require intervention to prevent one of the other outcomes listed above should also usually be considered serious. [ICH E2A]

Examples of such events are intensive treatment in an emergency room or at home for allergic bronchospasm, blood dyscrasias or convulsions that do not result in hospitalisation, or development of drug dependency or drug abuse.

SAEs should be followed until resolution or stabilisation. Participants with ongoing SAEs at study termination (including safety visit) will be further followed up until recovery or until stabilisation of the disease after termination.

Assessment of Causality

Both Investigator and Sponsor-investigator make a causality assessment of the event to the study drug, based on the criteria listed in the ICH E2A guidelines:

Table 4: Categories of causality assessment.

Relationship	Description
Causal relationship	Temporal relationship

	Improvement after dechallenge*<br>Recurrence after rechallenge<br>(or other proof of drug cause)
Probably	Temporal relationship<br>Improvement after dechallenge<br>No other cause evident
Possibly	Temporal relationship<br>Other cause possible
Unlikely	Any assessable reaction that does not fulfil the above conditions
Not related	Causal relationship can be ruled out
*Improvement after dechallenge only taken into consideration, if applicable to reaction	

Unexpected Adverse Drug Reaction

An "unexpected" adverse drug reaction is an adverse reaction, the nature or severity of which is not consistent with the applicable product information. [ICH E2A]

Suspected Unexpected Serious Adverse Reactions (SUSARs)

The Sponsor-Investigator evaluates any SAE that has been reported regarding seriousness, causality and expectedness. If the event is related to the investigational product and is both serious and unexpected, it is classified as a SUSAR.

Unblinding is needed in order to determine a SUSAR. Treatment allocation should not be disclosed to the investigator, nor to the study staff, in order not to make the subject ineligible.

Assessment of Severity

For severity grading of AEs in this study, depending on the type of study and disease, the grades for severity are described in the "Common Terminology Criteria for Adverse Events (CTCAE) version 5.0 terminology [118].

10.1.2 Reporting of serious adverse events (SAE) and other safety related events

The investigators are responsible for SAE reporting to the CEC according to the following details:

Reporting of SAEs

All SAEs must be reported immediately and within a maximum of 24 hours to the Sponsor-Investigator of the study. The Sponsor-Investigator will re-evaluate the SAE and return the form to the site.

SAEs resulting in death are reported to the Ethics Committee via BASEC within 7 days.

There are no SAEs that are exempted from expedited reporting.

SAEs or other safety relevant events must be also reported to the Marketing Approval Holder (MAH) of the drug (AstraZeneca AG). The details are documented in the Global PS guideline for externally sponsored research (ESR) document (Version 4, 8th February 2019, see APPENDICES) provided by AZ.

Reporting of SUSARs

A SUSAR needs to be reported to the Ethics Committee (local event via local Investigator) via BASEC and to Swissmedic (via Sponsor-Investigator) within 7 days, if the event is fatal, or within 15 days (all other events).

Reporting of Safety Signals

All suspected new risks and relevant new aspects of known adverse reactions that require safety-related measures, i.e. so called safety signals, must be reported to the Sponsor-Investigator within 24 hours. The Sponsor-Investigator must report the safety signals within 7 days to the Ethics Committee (local event via local Investigator) via BASEC and to Swissmedic.

Reporting and Handling of Pregnancies

During the screening visit prior to the study enrolment (see also 9.3.1) a pregnancy test will be performed in premenopausal (or postmenopausal < 2 years) female patients with child bearing potential. Furthermore, these patients will be instructed orally and in written form (study information sheet, PIC) to use reliable forms of contraception throughout the study period and for additional 3 months thereafter.

Pregnancy itself is not regarded as an AE unless there is a suspicion that the investigational product under study may have interfered with the effectiveness of a contraceptive medication.

Pregnant participants must immediately be withdrawn from the clinical study. Any pregnancy during the treatment phase of the study and within 30 days after discontinuation of study medication will be reported to the Sponsor-Investigator within 24 hours. The course and outcome of the pregnancy will be followed up carefully, and any abnormal outcome regarding the mother or the child must be documented and reported.

Periodic reporting of safety

An annual safety report is submitted once a year to the local Ethics Committee via local Investigator and to Swissmedic via Sponsor-Investigator.

10.1.3 Follow up of Serious Adverse Events

Participants terminating the main study (V2-V5) (either regularly or prematurely) with

reported ongoing SAE

any ongoing potentially IMP-related AEs will return for a follow-up investigation.

This visit will take place up to 30 days after terminating the treatment period.

Follow-up information on the outcome will be recorded on the respective AE page in the eCRF.

All other information has to be documented in the source documents. Source data has to be available upon request.

In case of participants lost to follow-up, efforts should be made and documented to contact the participant to encourage him/her to continue study participation as scheduled.

Follow-up investigations may also be necessary according to the investigator’s medical judgment even if the participant has no SAE at the end of the study.

11. HOWEVER, INFORMATION RELATED TO THESE INVESTIGATIONS DOES NOT HAVE TO BE DOCUMENTED IN THE ECRF BUT MUST BE NOTED IN THE SOURCE DOCUMENTS.STATISTICAL METHODS

11.1 Hypothesis

We hypothesize that dapagliflozin on top of recommended therapy improves relative plasma volume status (PVS) as mean change from baseline to 12 weeks of treatment period (ΔPVS) in comparison to placebo in clinically compensated heart failure patients after hospitalization because of an acute decompensated (congestive) heart failure (ADHF) event.

Furthermore, we hypothesize that dapgliflozin treatment improves vascular function compared to placebo with additive beneficial effects on hemodynamics and tissue oxygen supply resulting in an improved cardiac function, exercise capacity and health-related quality of life (HRQoL), and reduce the incidences of worsening heart failure (WHF) and rehospitalization in these patients.

An improved PVS is considered as a shift of measured plasma volume (mPV) closer to sex-dependent ideal plasma volume (iPV) as calculated by use of the ‘Hakim formula’[36].

Subsequently, dapagliflozin treatment is expected to shift and stabilize the PVS towards ‘euvolemia’

(PVS= 0).

Formulation of main study hypothesis for the main outcome variable:

H_0: The ΔPVS is not different between DAPA- and PLA-group.

H_1: The ΔPVS is different between DAPA- and PLA-group.

11.2 Determination of Sample Size

A sample size estimation was performed for the primary study outcome (ΔPVS) using R (V3.6.1) and PASS (V20.0.1). The sample size calculation comprised 2 primary analyses, which lead to the conclusion that a total sample size of 80 patients is sufficient to reach a power of ≥ 80% for the primary outcome test analysis (mixed model for repeated measures, MMRM):

1. ANCOVA:

The calculation is based on a two-step approach for an ANCOVA test setting as proposed by Borm et al. [119].

In a first step, the sample size required for a t-test (type I error probability: 5%, two-sided) was calculated by assuming a moderate effect size (d= 0.5) to reach a power of 80%. It revealed a sample size of n= 63.8 per group, which was rounded up to 64.

In a second step, the t-test sample size was multiplied by a ‘design factor’ (z), which was calculated as follows: $z = (1 - p^2)*n$, p = correlation of the mean of outcome variable Y at baseline (Y_0) and after the treatment period (12 weeks) (Y1), which was set at 0.6, n = calculated t-test total sample size (n= 128).

Subsequently, the ANCOVA-based power analysis revealed a total sample size of 81.9 to reach a power of ≥ 0.8.

2. MMRM:

By using the data obtained from the work of Dekkers et al. [75] on dapagliflozin on estimated plasma volume (ePV), a power calculation for a 2-level hierarchical, longitudinal mixed model design (2 group means at the end of follow-up) [120] was performed and revealed a power ≥ 0.9 when the ANCOVA sample size was applied.

Conclusion: The MMRM is favoured as primary outcome test analysis and the subsequent power analysis revealed that a total sample size of 80 is sufficient to reach a power of ≥ 80% for the primary outcome.

11.3 Statistical criteria of termination of trial

The study is not intended to be prematurely terminated on the basis of superior efficacy determined by a statistically significant result regarding the primary outcome at an interim analysis (IA), however with exception of safety concerns (see also 11.4.4).

11.4 Planned Analyses

11.4.1 Datasets to be analysed, analysis populations

Statistical analyses (primary and secondary) will be performed by the investigators with support from a biostatistician using IBM SPSS V25 (Chicago, IL, USA), R statistics software V3.6.1 (R foundation of statistical computing) and GraphPad Prism software package V8.0 (San Diego, CA, USA).

The dataset will comprise all patients initially randomized.

All data will be tested for normal distribution with the Shapiro-Wilk test and by graphical evaluation using histograms and Q-Q-plots.

The homogeneity of variances will be checked with Levene’s test.

In case of non-normal distribution, variables will be either logarithmically transformed to approximate

normality before parametric testing will be applied or if applicable a corresponding non-parametric test will be used.

Note: The data analysis of the planed prospective observational follow-up study visit (Fig. 1: V6, 12 months after the end of IMP treatment) will not be included in the main study evaluation and analyzed separately to avoid delays to obtain the outcome results of the main study.

11.4.2 Primary Analysis

For the primary analysis, a mixed model for repeated measures (MMRM) - will be applied for continuous outcome variables (e.g. ΔPVS) to test for a potential treatment effect between groups (DAPA vs. PLA).

Rationale: Although the ANCOVA test is considered as statistical 'robust' and 'efficient' test for analysing continuous outcomes requiring covariate adjustment in RCTs [121] and was previously used by others in a comparable study setting [122]. However, the MMRM has the advantage to better handle missing data at random and thus avoids to 'loose' subjects in the data analysis.

For testing of potential associations between outcome variables Pearson's correlation coefficient (r) will be used or if case of non-normal distributed data the Spearman's rank correlation coefficient (ρ) will be used. A p-value of < 0.05 is considered to indicate statistical significance.

11.4.3 Secondary Analyses

For the secondary analysis, considering the changes of outcome variables from baseline over time either a 2-way repeated measures ANOVA with time as within-subject factor and treatment as between subject factor or a comparable linear mixed effect model (lme) with defined fixed factors (e.g. treatment) and random factors (e.g. patient id) considering potential interactions between factors (e.g. between time and treatment) will be applied to evaluate the potential treatment effect at different time points within the 12-weeks observation period (see 9.2).

11.4.4 Interim analyses

Based on the various defined outcomes of the study (see 9.), which are supposed to provide valuable mechanistic insight into the effects of dapagliflozin on cardiac- and vascular function apart from the defined primary outcome variable no 'negative stopping' based on an a priori interim analysis (IA) analysis of the primary outcome is planned [123].

However, if the descriptive evaluation of defined safety outcomes (see 9.2.4) indicates higher than expected incidences of adverse reactions an unplanned IA might be performed by an independent biostatistician who will not be involved in the trial. The results will be reviewed by an independent Data Monitoring Committee.

11.4.5 Safety analysis

Descriptive safety analyses will be performed based on defined safety outcome variables (see 9.2.4).

However, no formal statistical inference analysis will be performed.

11.4.6 Deviation(s) from the original statistical plan

Investigators will avoid any substantial deviation from the original statistical plan. Yet, if a consensus is reached in a particular deviation that will improve the analysis of the data, a deviation from the planned analyses is justified and a protocol amendment will be proposed for approval by CEC and the deviation reported in the clinical study report.

11.5 Handling of missing data and drop-outs

Study dropouts will be listed, showing reasons for discontinuation. All patients originally randomized will be included in the intent to treat (ITT) analysis [124] (patients will be assigned to treatment groups

as initially randomized).

Additional patients will be recruited and randomised to replace potential dropouts of the main study in order to maintain sufficient statistical power for the primary outcome analysis.

12. QUALITY ASSURANCE AND CONTROL

The Sponsor-Investigator is implementing and maintaining quality assurance and quality control systems with written standard operating procedures (SOPs) and working instructions (WIs) to ensure that trials are conducted and data are generated, documented (record), and reported in compliance with the protocol, GCP, and applicable regulatory requirement(s).

Monitoring will be conducted during the course of the study for quality assurance purposes (see also 12.3). Internal Auditing may be planned (see also 12.4).

12.1 Data handling and record keeping / archiving

The study will strictly follow the protocol. If any changes become necessary, they must be laid down in an amendment to the protocol. All amendments of the protocol must be signed by the Sponsor-Investigator and if essential submitted to CEC.

12.1.1 Case Report Forms

The investigators will use electronic case report forms (eCRF), one for each enrolled study participant, to be filled in with all relevant data pertaining to the participant during the study.

All participants who either entered the study or were considered not-eligible or were eligible but not enrolled into the study additionally have to be documented on a screening log.

The investigator will document the participation of each study participant on the enrolment log.

For data and query management, monitoring, reporting and coding an internet-based secure data base secuTrial® developed in agreement to the good clinical practice (GCP) guidelines provided by the clinical trials centre (CTC) Zurich will be used for this study.

It is the responsibility of the investigator to assure that all data in the course of the study will be entered completely and correctly in the respective data base.

Corrections in the eCRF may only be done by the investigator or by other authorized persons. In case of corrections the original data entries will be archived in the system and can be made visible. For all data entries and corrections date, time of day and person who is performing the entries will be generated automatically.

The eCRFs will be kept current to reflect participant status at each phase during the course of study. Participants must not to be identified in the eCRFs by name. Appropriate coded identification (e.g. Participant Number) must be used.

Initials must not be used in combination with the date of birth in the eCRF for identification of the study participant.

It must be assured that any authorized person, who may perform data entries and changes in the eCRFs, can be identified. A list with signatures and initials of all authorized persons will be filed in the study site file and the trial master file, respectively.

Documented medical histories and narrative statements relative to the participant's progress during the study will be maintained. These records will also include the following: originals or copies of laboratory and other medical test results (e.g. ECGs, etc.) which must be kept on file with the individual participant's eCRF.

The investigators assure to perform a complete and accurate documentation of the participant data in the eCRFs. All data entered into eCRFs will also be available in the individual participant file either as print-outs or as notes taken by either the investigator or another responsible person assigned by the investigator.

Essential documents will be retained for at least 10 years after the regular end or a premature termination of the respective study (ClinO Art. 45).

Any patient files and source data must be archived for the longest possible period of time according to

the feasibility of the investigational site, e.g. hospital, institution or private practice.

12.1.2 Specification of source documents

The following documents are considered source data, including but not limited to:

- SAE worksheets,
- pCRFs,
- Nurse records, records of clinical coordinators, and
- Medical records from other department(s), or other hospital(s), or discharge letters and correspondence with other departments/hospitals, if participant visited any during the study period and the post study period;

Source data must be available at the site to document the existence of the study participants and substantiate the integrity of study data collected. Source data must include the original documents relating to the study, as well as the medical treatment and medical history of the participant.

The following information (at least but not limited to) should be included in the source documents:

- Demographic data (age, sex, ethnicity etc.);
- Inclusion and Exclusion Criteria details;
- Participation in study and signed and dated informed consent forms;
- Visit dates;
- Medical history and physical examination details;
- Key efficacy and safety data (as specified in the protocol);
- AEs and concomitant medication;
- Results of relevant examinations;
- Laboratory printouts;
- Dispensing and return of study drug details;
- Reason for premature discontinuation;
- Randomization number;

12.1.3 Record keeping / archiving

All study data must be archived for a minimum of 10 years after study termination or premature termination of the clinical trial. For electronic data see section 12.1.1.

The study data will be stored in a secured place with locking option in the clinical research laboratory of cardiology (room: RAE E30) at the USZ.

12.2 Data management

12.2.1 Data Management System

The study will strictly follow the study protocol. If any changes become necessary, they must be laid down in an amendment to the protocol. All amendments of the protocol must be signed by the Sponsor-Investigator and submitted to CEC and Swissmedic.

The investigators will use study specific eCRFs, one for each enrolled study participant, to be filled in with all relevant data pertaining to the subject during the study.

All subjects who either entered the study or were considered not-eligible or were eligible but not enrolled into the study additionally have to be documented on a screening log.

The investigators will document the participation of each study subject on the enrolment log.

For data and query management, monitoring, reporting and coding an internet-based secure data

base secuTrial® developed in agreement to GCP-guidelines provided by the CTC Zurich will be used for this study. It is the responsibility of the investigators to assure that all data in the course of the study will be entered completely and correctly in the respective data base.

Corrections in the eCRF may only be done by the investigators or by authorized staff. In case of corrections, the original data entries will be archived in the system and can be made visible. For all data entries and corrections date, time of day and person who is performing the entries will be generated automatically.

The eCRFs must be kept current to reflect subject status at each phase during the course of study. Subjects must not to be identified in the eCRF by name. Appropriate coded identification (e.g. subject number) must be used.

Initials must not be used in combination date of birth or year of birth in the eCRF for identification of the study subject.

It must be assured that any authorized person, who may perform data entries and changes in the eCRF, can be identified. A list with signatures and initials of all authorized persons will be filed in the study site file and the trial master file, respectively.

Documented medical histories and narrative statements relative to the subject's progress during the study will be maintained. These records will also include the following: Originals or copies of laboratory and other medical test results (e.g. ECGs, etc.) which must be kept on file with the individual subject's eCRF. The investigators assure to perform a complete and accurate documentation of the subject data in the eCRF.

All data entered into the eCRF must also be available in the individual subject file either as print-outs or as notes taken by either the investigator or another responsible person assigned by the investigator.

Essential documents must be retained for at least 10 years after the regular end or a premature termination of the respective study (VKlin Art. 45).

Any patient files and source data must be archived for the longest possible period (10 years) of time according to the feasibility of the investigational site (University hospital of Zurich).

<u>DAPA-VOLVO-Follow-U (V6):</u> As stated the optional follow-up visit V6 12 months after the final visit (V5) of the main study (V2-V5) will be evaluated and analysed independently from the main study. The data will be documented on pCRF and transferred to eCRF within USZ CTC-supported REDCap® electronic data base.

12.2.2 Data security, access and back-up

For the eCRFs see section 12.2.1. For source documents: Electronic data will be stored on a secure server on site with individual password protection per user, logging of user activity, and an encrypted back-up system. Only authorized personnel as defined on the delegation log will have access to the data. All computers used in the study are behind a firewall and run anti-virus software.

12.2.3 Analysis and archiving

Data will be exported from the secuTrial®/ REDCap® electronic data base for statistical analysis by use of statistical software as described in section 11.4.1. The electronic data base will be archived by use of secuTrial® software as previously described (see 12.2.2). Paper source documentation is kept on site as described in section 12.1.3.

12.2.4 Electronic and central data validation

Electronic and central validation is insured by secuTrial ® software system. Input fields allow for discreet variables only to be chosen from a drop-down menu, if values exceed the range of common-sense a warning will be issued by the eCRF form to check the input before confirmation.

12.3 Monitoring

Monitoring visits at the investigator’s site prior to the start and during the course of the study will help

to follow up the progress of the clinical study, to assure utmost accuracy of the data and to detect possible errors at an early time point. The Sponsor-Investigator organizes professional independent monitoring for the study.

All original data including all patient files, progress notes and copies of laboratory and medical test results must be available for monitoring. The monitor will review all or a part of the eCRFs and written informed consents.

The accuracy of the data will be verified by reviewing the above referenced documents. The investigator's site will collaborate with the Clinical Trials Center (CTC) of the University Hospital Zurich to ensure monitoring. According to the CTC's Monitoring SOP the extent and nature of monitoring activities based on the objective and design of the study will be defined in a study specific monitoring plan.

12.4 Audits and Inspections

An independent quality assurance audit/inspection of this study may be conducted by the competent authority or CEC, respectively. The quality assurance auditor/inspector will have access to all medical records, the investigator's study related files and correspondence, and the informed consent documentation that is relevant to this clinical study.

The investigator will allow the persons being responsible for the audit or the inspection to have access to the source data/documents and to answer any questions arising. All involved parties will keep the patient data strictly confidential.

12.5 Confidentiality, Data Protection

Direct access to source documents will be permitted for purposes of monitoring (12.3), audits and inspections (12.4) (ICHE6, 6.10) and authorized study personnel as defined on the delegation log have access to protocol, dataset, statistical code, etc. during and after the study (publication, dissemination).

Data generation, transmission, archiving and analysis of health related personal data and the storage of biological samples within this project strictly follow the current Swiss legal requirements for data protection and according to the ordinance of KlinV Art. 18. Prerequisite is the voluntary approval of the participant given by signing the informed consent prior start of participation of the research project.

Health related personal data captured during this project and biological samples from participants are strictly confidential and disclosure to third parties is prohibited; coding will safeguard participants' confidentiality. Confidentiality will be ensured by using coded data. The participant identification code will be protected against unauthorized access and will be stored appropriately by the responsible study coordinator.

The collected project data may be subject to inspection by the CEC.

Biological material in this study is not identified by participant name but by a unique participant code. Biological material is appropriately stored in a restricted area only accessible to the authorized personnel. As mentioned previously for the measurement of blood sample parameters that cannot be measured within the USZ laboratories (e.g. FGF23) numerically coded frozen plasma samples will be shipped on dry ice in protected and insulated boxes to the collaborative laboratory for further analysis.

Biological material can only be sent abroad in the scope of the research study, if the participant involved has given his or her consent (e.g. USZ general consent) to do so upon having been sufficiently informed. Non-genetic health-related personal data can be sent abroad for research if the requirements of Swiss data protection law are met (FADP, Art. 6).

Pseudonymized data and coded biological material may be shared with other collaborative investigators involved in / or outside the DAPA-VOLVO study project under the restrictions of the Swiss legal requirements and informed consent given by the patients.

Pseudonymized biological material or genetic personal data of the study participant can only be shared for further research purpose if the study participate has signed the study specific informed consent and/or the USZ-general informed consent (art. 29 HFV).

The participant will be orally and written informed (included in the patient information sheet) about their right to withdrawal from the given consent to share pseudonymized biological material and (genetic) personal data (art. 29 HFV) as well as study data according to HFV (art. 32).

12.6 Storage of biological material and related health data

All study data will be archived according to local legislation for 10 years after study termination or premature termination of the study.

Blood for routinely available laboratory analysis (e.g. blood cell count, potassium) will be performed within 24h after sampling.

Blood samples for further analysis will be stored at -80°C in a temperature monitored freezer on sight of investigation, USZ, until finalization of analysis and publication of the study results.

Thereafter the blood samples and urine samples will be destroyed and a confirmation note to file sheet will be added to the trial master file.

13. PUBLICATION AND DISSEMINATION POLICY

After the statistical data analysis the study results will be presented at international cardiology congresses by an investigator who made a major contribution to the study project and the sponsor will make every effort to publish the data in a peer-reviewed medical journal.

14. FUNDING AND SUPPORT

14.1 Funding

The DAPA-VOLVO project will be funded by the externally sponsored scientific research program of AstraZeneca AG. The corresponding study contracts will be in the study trial master file.

14.2 Other Support

N.A.

15. INSURANCE

Insurance is covered by “Versicherung für klinische Versuche und nichtklinische Versuche“ by Zürich Versicherungs-Gesellschaft AG (Policy no: 14.970.888).

Any damage developed in relation to study participation is covered by this insurance. So as not to forfeit their insurance cover, the participants themselves must strictly follow the instructions of the study personnel. Participants must not be involved in any other medical treatment without permission of the principal investigator (emergency excluded). Medical emergency treatment must be reported immediately to the investigator. The investigator must also be informed instantly, in the event of health problems or other damages during or after the course of study treatment.

The investigator will allow delegates of the insurance company to have access to the source data/documents as necessary to clarify a case of damage related to study participation. All involved parties will keep the patient data strictly confidential.

A copy of the insurance certificate will be placed in the trial master file.

16. REFERENCES

1. Grote Beverborg, N., D.J. van Veldhuisen, and P. van der Meer, *Anemia in Heart Failure: Still Relevant?* JACC Heart Fail, 2018. **6**(3): p. 201-208.
2. Miller, W.L. and B.P. Mullan, *Volume Overload Profiles in Patients With Preserved and Reduced Ejection Fraction Chronic Heart Failure: Are There Differences? A Pilot Study.* JACC Heart Fail, 2016. **4**(6): p. 453-9.
3. Buglioni, A. and J.C. Burnett, Jr., *Pathophysiology and the cardiorenal connection in heart failure. Circulating hormones: biomarkers or mediators.* Clin Chim Acta, 2015. **443**: p. 3-8.
4. Ghosh, R.K., et al., *Sodium Glucose Co-transporter 2 Inhibitors and Heart Failure.* Am J Cardiol, 2019.
5. McMurray, J.J.V., et al., *Dapagliflozin in Patients with Heart Failure and Reduced Ejection Fraction.* N Engl J Med, 2019.
6. Patel, D.K. and J. Strong, *The Pleiotropic Effects of Sodium-Glucose Cotransporter-2 Inhibitors: Beyond the Glycemic Benefit.* Diabetes Ther, 2019. **10**(5): p. 1771-1792.
7. Katz, S.D., *In search of euvolemia in heart failure.* JACC Heart Fail, 2014. **2**(3): p. 306-7.
8. Greene, S.J., et al., *The vulnerable phase after hospitalization for heart failure.* Nat Rev Cardiol, 2015. **12**(4): p. 220-9.
9. Ling, H.Z., et al., *Calculated plasma volume status and prognosis in chronic heart failure.* Eur J Heart Fail, 2015. **17**(1): p. 35-43.
10. Martens, P., et al., *The Optimal Plasma Volume Status in Heart Failure in Relation to Clinical*

Outcome. J Card Fail, 2019. **25**(4): p. 240-248.

11. Ponikowski, P., et al., *2016 ESC Guidelines for the diagnosis and treatment of acute and chronic heart failure: The Task Force for the diagnosis and treatment of acute and chronic heart failure of the European Society of Cardiology (ESC)Developed with the special contribution of the Heart Failure Association (HFA) of the ESC.* Eur Heart J, 2016. **37**(27): p. 2129-2200.
12. WHO, *WHO, International Clinical Trials Registry Platform (ICTRP).*
13. WMA, *Declaration of Helsinki*. 2013, World Medical Association (WMA).
14. ICH, *International Conference on Harmonization (ICH, 1996) E6 (R2) Guideline for Good Clinical Practice.* 2016.
15. ICH, *International Conference on Harmonization (ICH, 1997) E8 Guideline: General Considerations for Clinical Trials*. 1997.
16. Stewart, S., et al., *More 'malignant' than cancer? Five-year survival following a first admission for heart failure.* Eur J Heart Fail, 2001. **3**(3): p. 315-22.
17. Teerlink, J.R., et al., *Acute decompensated heart failure update.* Curr Cardiol Rev, 2015. **11**(1): p. 53-62.
18. Mullens, W., et al., *The use of diuretics in heart failure with congestion - a position statement from the Heart Failure Association of the European Society of Cardiology.* Eur J Heart Fail, 2019. **21**(2): p. 137-155.
19. Miller, W.L., *Fluid Volume Overload and Congestion in Heart Failure: Time to Reconsider Pathophysiology and How Volume Is Assessed.* Circ Heart Fail, 2016. **9**(8): p. e002922.
20. Braam, B., et al., *Cardiorenal syndrome--current understanding and future perspectives.* Nat Rev Nephrol, 2014. **10**(1): p. 48-55.
21. Fudim, M., A.F. Hernandez, and G.M. Felker, *Role of Volume Redistribution in the Congestion of Heart Failure.* J Am Heart Assoc, 2017. **6**(8).
22. Dekker, M.J.E., et al., *Fluid Overload and Inflammation Axis.* Blood Purif, 2018. **45**(1-3): p. 159-165.
23. Van Linthout, S. and C. Tschope, *Inflammation - Cause or Consequence of Heart Failure or Both?* Curr Heart Fail Rep, 2017. **14**(4): p. 251-265.
24. Colombo, P.C., et al., *Venous congestion, endothelial and neurohormonal activation in acute decompensated heart failure: cause or effect?* Curr Heart Fail Rep, 2015. **12**(3): p. 215-22.
25. Weiss, G., T. Ganz, and L.T. Goodnough, *Anemia of inflammation.* Blood, 2019. **133**(1): p. 40-50.
26. van Veldhuisen, D.J., et al., *Anemia and iron deficiency in heart failure: mechanisms and therapeutic approaches.* Nat Rev Cardiol, 2011. **8**(9): p. 485-93.
27. Anand, I.S. and P. Gupta, *Anemia and Iron Deficiency in Heart Failure: Current Concepts and Emerging Therapies.* Circulation, 2018. **138**(1): p. 80-98.
28. Titze, J., *Sodium balance is not just a renal affair.* Curr Opin Nephrol Hypertens, 2014. **23**(2): p. 101-5.
29. Wiig, H., et al., *Immune cells control skin lymphatic electrolyte homeostasis and blood pressure.* J Clin Invest, 2013. **123**(7): p. 2803-15.
30. Androne, A.S., et al., *Hemodilution is common in patients with advanced heart failure.* Circulation, 2003. **107**(2): p. 226-9.
31. Adlbrecht, C., et al., *Chronic heart failure leads to an expanded plasma volume and pseudoanaemia, but does not lead to a reduction in the body's red cell volume.* Eur Heart J, 2008. **29**(19): p. 2343-50.
32. Montero, D., et al., *True Anemia-Red Blood Cell Volume Deficit-in Heart Failure: A Systematic Review.* Circ Heart Fail, 2017. **10**(5).
33. Otto, J.M., et al., *Hemoglobin concentration, total hemoglobin mass and plasma volume in patients: implications for anemia.* Haematologica, 2017. **102**(9): p. 1477-1485.
34. Montero, D., et al., *Hypovolemia and reduced hemoglobin mass in patients with heart failure and preserved ejection fraction.* Physiol Rep, 2019. **7**(21): p. e14222.
35. Strobeck, J.E., J. Feldschuh, and W.L. Miller, *Heart Failure Outcomes With Volume-Guided Management.* JACC Heart Fail, 2018.

36. Hakim, R.M., *Plasmapheresis. In Daugirdas JT, Blake PG, Ing TS, eds. Handbook of dialysis.* 3rd ed. 2001: Lippincott, Williams and Wilkins.

37. Grodin, J.L., et al., *Prognostic implications of plasma volume status estimates in heart failure with preserved ejection fraction: insights from TOPCAT.* Eur J Heart Fail, 2019. **21**(5): p. 634-642.

38. Maznyczka, A.M., et al., *Calculated plasma volume status and outcomes in patients undergoing coronary bypass graft surgery.* Heart, 2019. **105**(13): p. 1020-1026.

39. Gore, C.J., W.G. Hopkins, and C.M. Burge, *Errors of measurement for blood volume parameters: a meta-analysis.* J Appl Physiol (1985), 2005. **99**(5): p. 1745-58.

40. Schmidt, W. and N. Prommer, *The optimised CO-rebreathing method: a new tool to determine total haemoglobin mass routinely.* Eur J Appl Physiol, 2005. **95**(5-6): p. 486-95.

41. Keiser, S., et al., *Detection of blood volumes and haemoglobin mass by means of CO re-breathing and indocyanine green and sodium fluorescein injections.* Scand J Clin Lab Invest, 2017. **77**(3): p. 164-174.

42. Ahlgrim, C., Birkner P., Seiler F., Grundmann S., Baumstark MW., Bode C. and Pottgiesser T., *Applying the Optimized CO Rebreathing Method for Measuring Blood Volumes and Hemoglobin Mass in Heart Failure Patients.* Front Physiol, 2018. **9**(1603).

43. Lundby, C., et al., *Red blood cell volume is not decreased in ESA-naive anemic chronic kidney disease patients.* Physiol Rep, 2018. **6**(21): p. e13900.

44. Bomholt, T., et al., *Intravascular volumes evaluated by a carbon monoxide rebreathing method in patients undergoing chronic hemodialysis.* Hemodial Int, 2020.

45. Montero, D., et al., *Effects of hemodialysis on blood volume, macro- and microvascular function.* Microvasc Res, 2020. **129**: p. 103958.

46. Plumb, J.O.M., et al., *Application of the optimized carbon monoxide rebreathing method for the measurement of total haemoglobin mass in chronic liver disease.* Physiol Rep, 2020. **8**(6): p. e14402.

47. Kluger, A.Y., et al., *Cardiorenal Outcomes in the CANVAS, DECLARE-TIMI 58, and EMPA-REG OUTCOME Trials: A Systematic Review.* Rev Cardiovasc Med, 2018. **19**(2): p. 41-49.

48. Fitchett, D., et al., *Empagliflozin Reduced Mortality and Hospitalization for Heart Failure Across the Spectrum of Cardiovascular Risk in the EMPA-REG OUTCOME Trial.* Circulation, 2019. **139**(11): p. 1384-1395.

49. Fitchett, D., et al., *Heart failure outcomes with empagliflozin in patients with type 2 diabetes at high cardiovascular risk: results of the EMPA-REG OUTCOME(R) trial.* Eur Heart J, 2016. **37**(19): p. 1526-34.

50. Petrie, M.C., et al., *Effect of Dapagliflozin on Worsening Heart Failure and Cardiovascular Death in Patients With Heart Failure With and Without Diabetes.* JAMA, 2020.

51. Lytvyn, Y., et al., *Sodium Glucose Cotransporter-2 Inhibition in Heart Failure: Potential Mechanisms, Clinical Applications, and Summary of Clinical Trials.* Circulation, 2017. **136**(17): p. 1643-1658.

52. Heerspink, H.J.L., et al., *Renoprotective effects of sodium-glucose cotransporter-2 inhibitors.* Kidney Int, 2018. **94**(1): p. 26-39.

53. Heerspink, H.J.L., et al., *Rationale and protocol of the Dapagliflozin And Prevention of Adverse outcomes in Chronic Kidney Disease (DAPA-CKD) randomized controlled trial.* Nephrol Dial Transplant, 2020. **35**(2): p. 274-282.

54. AG, A., *Farxiga Phase III DAPA-CKD trial update statement- early trial stop after overwhelming drug efficiency* 2020.

55. Wojcik, C. and B.A. Warden, *Mechanisms and Evidence for Heart Failure Benefits from SGLT2 Inhibitors.* Curr Cardiol Rep, 2019. **21**(10): p. 130.

56. Filippatos, T.D., et al., *SGLT2 inhibitors and cardioprotection: a matter of debate and multiple hypotheses.* Postgrad Med, 2019. **131**(2): p. 82-88.

57. Verma, S. and J.J.V. McMurray, *SGLT2 inhibitors and mechanisms of cardiovascular benefit: a state-of-the-art review.* Diabetologia, 2018. **61**(10): p. 2108-2117.

58. Zelniker, T.A. and E. Braunwald, *Clinical Benefit of Cardiorenal Effects of Sodium-Glucose Cotransporter 2 Inhibitors: JACC State-of-the-Art Review.* J Am Coll Cardiol, 2020. **75**(4): p.

435-447.
59. Zelniker, T.A. and E. Braunwald, *Mechanisms of Cardiorenal Effects of Sodium-Glucose Cotransporter 2 Inhibitors: JACC State-of-the-Art Review.* J Am Coll Cardiol, 2020. **75**(4): p. 422-434.
60. Inzucchi, S.E., et al., *How Does Empagliflozin Reduce Cardiovascular Mortality? Insights From a Mediation Analysis of the EMPA-REG OUTCOME Trial.* Diabetes Care, 2018. **41**(2): p. 356-363.
61. Eickhoff, M.K., et al., *Effects of Dapagliflozin on Volume Status When Added to Renin-Angiotensin System Inhibitors.* J Clin Med, 2019. **8**(6).
62. Hallow, K.M., et al., *Why do SGLT2 inhibitors reduce heart failure hospitalization? A differential volume regulation hypothesis.* Diabetes Obes Metab, 2018. **20**(3): p. 479-487.
63. Ohara, K., et al., *The extracellular volume status predicts body fluid response to SGLT2 inhibitor dapagliflozin in diabetic kidney disease.* Diabetol Metab Syndr, 2020. **12**: p. 37.
64. Ohara, K., et al., *Effects of the sodium-glucose cotransporter 2 inhibitor dapagliflozin on fluid distribution: A comparison study with furosemide and tolvaptan.* Nephrology (Carlton), 2019. **24**(9): p. 904-911.
65. Mazer, C.D., et al., *Effect of Empagliflozin on Erythropoietin Levels, Iron Stores and Red Blood Cell Morphology in Patients with Type 2 Diabetes and Coronary Artery Disease.* Circulation, 2019.
66. Ghanim, H., et al., *Dapagliflozin Suppresses Hepcidin And Increases Erythropoiesis.* J Clin Endocrinol Metab, 2020. **105**(4).
67. Heerspink, H.J.L., et al., *Reduction in albuminuria with dapagliflozin cannot be predicted by baseline clinical characteristics or changes in most other risk markers.* Diabetes Obes Metab, 2019. **21**(3): p. 720-725.
68. Bonilla-Palomas, J.L., et al., *Hypoalbuminemia in acute heart failure patients: causes and its impact on hospital and long-term mortality.* J Card Fail, 2014. **20**(5): p. 350-8.
69. Peng, W., et al., *Prediction of all-cause mortality with hypoalbuminemia in patients with heart failure: a meta-analysis.* Biomarkers, 2019. **24**(7): p. 631-637.
70. Ancion, A., et al., *Serum albumin level and long-term outcome in acute heart failure.* Acta Cardiol, 2019. **74**(6): p. 465-471.
71. Katz, S.D., et al., *Vascular endothelial dysfunction and mortality risk in patients with chronic heart failure.* Circulation, 2005. **111**(3): p. 310-4.
72. Zuchi, C., et al., *Role of endothelial dysfunction in heart failure.* Heart Fail Rev, 2020. **25**(1): p. 21-30.
73. Komarova, Y. and A.B. Malik, *Regulation of endothelial permeability via paracellular and transcellular transport pathways.* Annu Rev Physiol, 2010. **72**: p. 463-93.
74. Batzias, K., et al., *Effects of Newer Antidiabetic Drugs on Endothelial Function and Arterial Stiffness: A Systematic Review and Meta-Analysis.* J Diabetes Res, 2018. **2018**: p. 1232583.
75. Dekkers, C.C.J., et al., *Effects of the sodium-glucose co-transporter-2 inhibitor dapagliflozin on estimated plasma volume in patients with type 2 diabetes.* Diabetes Obes Metab, 2019. **21**(12): p. 2667-2673.
76. Rehman, S., A. Khan, and A. Rehman, *Physiology, Coronary Circulation*, in *StatPearls*. 2020: Treasure Island (FL).
77. Jensen, R.V., M.V. Hjortbak, and H.E. Botker, *Ischemic Heart Disease: An Update.* Semin Nucl Med, 2020. **50**(3): p. 195-207.
78. Pitoulis, F.G. and C.M. Terracciano, *Heart Plasticity in Response to Pressure- and Volume-Overload: A Review of Findings in Compensated and Decompensated Phenotypes.* Front Physiol, 2020. **11**: p. 92.
79. Burchfield, J.S., M. Xie, and J.A. Hill, *Pathological ventricular remodeling: mechanisms: part 1 of 2.* Circulation, 2013. **128**(4): p. 388-400.
80. Yancey, D.M., et al., *Cardiomyocyte mitochondrial oxidative stress and cytoskeletal breakdown in the heart with a primary volume overload.* Am J Physiol Heart Circ Physiol, 2015. **308**(6): p. H651-63.
81. AstraZeneca, *Forxiga (A10BK01 Dapagliflozin)- Swissmedic-approved national professional*

information 2020.

82. McMurray, J.J.V., et al., *The Dapagliflozin And Prevention of Adverse-outcomes in Heart Failure (DAPA-HF) trial: baseline characteristics.* Eur J Heart Fail, 2019. **21**(11): p. 1402-1411.

83. Huynh, K., *Dapagliflozin - a breakthrough in the search for drugs to treat HFrEF.* Nat Rev Cardiol, 2019. **16**(12): p. 700.

84. Docherty, K.F., et al., *Effects of dapagliflozin in DAPA-HF according to background heart failure therapy.* Eur Heart J, 2020.

85. Kosiborod, M.N., et al., *Effects of Dapagliflozin on Symptoms, Function, and Quality of Life in Patients With Heart Failure and Reduced Ejection Fraction: Results From the DAPA-HF Trial.* Circulation, 2020. **141**(2): p. 90-99.

86. Martinez, F.A., et al., *Efficacy and Safety of Dapagliflozin in Heart Failure With Reduced Ejection Fraction According to Age: Insights From DAPA-HF.* Circulation, 2020. **141**(2): p. 100-111.

87. Nassif, M.E., et al., *Dapagliflozin Effects on Biomarkers, Symptoms, and Functional Status in Patients With Heart Failure With Reduced Ejection Fraction: The DEFINE-HF Trial.* Circulation, 2019. **140**(18): p. 1463-1476.

88. FDA, *FDA Approval of Farxiga (dapagliflozin) for adults with heart failure with reduced ejection fraction to reduce the risk of cardiovascular death and hospitalization for heart failure*. 2020.

89. Zannad, F., et al., *SGLT2 inhibitors in patients with heart failure with reduced ejection fraction: a meta-analysis of the EMPEROR-Reduced and DAPA-HF trials.* Lancet, 2020. **396**(10254): p. 819-829.

90. Berg, D.D., et al., *Time to Clinical Benefit of Dapagliflozin and Significance of Prior Heart Failure Hospitalization in Patients With Heart Failure With Reduced Ejection Fraction.* JAMA Cardiol, 2021.

91. Damman, K., et al., *Randomized, double-blind, placebo-controlled, multicentre pilot study on the effects of empagliflozin on clinical outcomes in patients with acute decompensated heart failure (EMPA-RESPONSE-AHF).* Eur J Heart Fail, 2020. **22**(4): p. 713-722.

92. Cox, Z.L., et al., *Efficacy and safety of dapagliflozin in acute heart failure: Rationale and design of the DICTATE-AHF trial.* Am Heart J, 2021. **232**: p. 116-124.

93. Kobayashi, M., et al., *Prognostic value of estimated plasma volume in acute heart failure in three cohort studies.* Clin Res Cardiol, 2019. **108**(5): p. 549-561.

94. Chaplin, H., Jr., P.L. Mollison, and H. Vetter, *The body/venous hematocrit ratio: its constancy over a wide hematocrit range.* J Clin Invest, 1953. **32**(12): p. 1309-16.

95. Nagele, M.P., et al., *Retinal microvascular dysfunction in heart failure.* Eur Heart J, 2018. **39**(1): p. 47-56.

96. Flammer, A.J., et al., *The assessment of endothelial function: from research into clinical practice.* Circulation, 2012. **126**(6): p. 753-67.

97. Sudano, I., et al., *Acetaminophen increases blood pressure in patients with coronary artery disease.* Circulation, 2010. **122**(18): p. 1789-96.

98. Butlin, M. and A. Qasem, *Large Artery Stiffness Assessment Using SphygmoCor Technology.* Pulse (Basel), 2017. **4**(4): p. 180-192.

99. Pannier, B.M., et al., *Methods and devices for measuring arterial compliance in humans.* Am J Hypertens, 2002. **15**(8): p. 743-53.

100. Laurent, S., et al., *Expert consensus document on arterial stiffness: methodological issues and clinical applications.* Eur Heart J, 2006. **27**(21): p. 2588-605.

101. Green, C.P., et al., *Development and evaluation of the Kansas City Cardiomyopathy Questionnaire: a new health status measure for heart failure.* J Am Coll Cardiol, 2000. **35**(5): p. 1245-55.

102. Buysse, D.J., et al., *The Pittsburgh Sleep Quality Index: a new instrument for psychiatric practice and research.* Psychiatry Res, 1989. **28**(2): p. 193-213.

103. Kodama, S., et al., *Cardiorespiratory fitness as a quantitative predictor of all-cause mortality and cardiovascular events in healthy men and women: a meta-analysis.* JAMA, 2009. **301**(19): p. 2024-35.

104. Kaminsky, L.A., J. Myers, and R. Arena, *Determining Cardiorespiratory Fitness With Precision: Compendium of Findings From the FRIEND Registry.* Prog Cardiovasc Dis, 2019. **62**(1): p. 76-

82.
105. Guazzi, M., et al., *EACPR/AHA Joint Scientific Statement. Clinical recommendations for cardiopulmonary exercise testing data assessment in specific patient populations.* Eur Heart J, 2012. **33**(23): p. 2917-27.
106. Guazzi, M., et al., *2016 focused update: clinical recommendations for cardiopulmonary exercise testing data assessment in specific patient populations.* Eur Heart J, 2018. **39**(14): p. 1144-1161.
107. Uszko-Lencer, N., et al., *Reliability, construct validity and determinants of 6-minute walk test performance in patients with chronic heart failure.* Int J Cardiol, 2017. **240**: p. 285-290.
108. Grassi, G., F. Quarti-Trevano, and M.D. Esler, *Sympathetic activation in congestive heart failure: an updated overview.* Heart Fail Rev, 2019.
109. White, D.W., J.K. Shoemaker, and P.B. Raven, *Methods and considerations for the analysis and standardization of assessing muscle sympathetic nerve activity in humans.* Auton Neurosci, 2015. **193**: p. 12-21.
110. Victor, R.G., et al., *Effects of the cold pressor test on muscle sympathetic nerve activity in humans.* Hypertension, 1987. **9**(5): p. 429-36.
111. Fagius, J., S. Karhuvaara, and G. Sundlof, *The cold pressor test: effects on sympathetic nerve activity in human muscle and skin nerve fascicles.* Acta Physiol Scand, 1989. **137**(3): p. 325-34.
112. Parrinello, G., et al., *Water and sodium in heart failure: a spotlight on congestion.* Heart Fail Rev, 2015. **20**(1): p. 13-24.
113. Dyke, J.P., et al., *Reliability and agreement of sodium ((23)Na) MRI in calf muscle and skin of healthy subjects from the US.* Clin Imaging, 2018. **52**: p. 100-105.
114. Gomolka, R.S., et al., *Quantification of sodium T1 in abdominal tissues at 3 T.* MAGMA, 2020. **33**(3): p. 439-446.
115. Rossi, C., et al., *Histogram analysis of renal arterial spin labeling perfusion data reveals differences between volunteers and patients with mild chronic kidney disease.* Invest Radiol, 2012. **47**(8): p. 490-6.
116. Rossi, C., et al., *Blood oxygen level-dependent magnetic resonance imaging of the kidneys: influence of spatial resolution on the apparent R2* transverse relaxation rate of renal tissue.* Invest Radiol, 2013. **48**(9): p. 671-7.
117. Muller, L., et al., *Isolation of biologically-active exosomes from human plasma.* J Immunol Methods, 2014. **411**: p. 55-65.
118. (NIH), N.I.o.H., *Common Terminology Criteria for Adverse Events (CTCAE) v5.0*. 2017.
119. Borm, G.F., J. Fransen, and W.A. Lemmens, *A simple sample size formula for analysis of covariance in randomized clinical trials.* J Clin Epidemiol, 2007. **60**(12): p. 1234-8.
120. Ahn, C., Heo, M., and Zhang, S., *Sample Size Calculations for Clustered and Longitudinal Outcomes in Clinical Research*. 2015, New York: CRC Press.
121. Zhang, S., et al., *Empirical comparison of four baseline covariate adjustment methods in analysis of continuous outcomes in randomized controlled trials.* Clin Epidemiol, 2014. **6**: p. 227-35.
122. Lambers Heerspink, H.J., et al., *Dapagliflozin a glucose-regulating drug with diuretic properties in subjects with type 2 diabetes.* Diabetes Obes Metab, 2013. **15**(9): p. 853-62.
123. Kumar, A. and B.S. Chakraborty, *Interim analysis: A rational approach of decision making in clinical trial.* J Adv Pharm Technol Res, 2016. **7**(4): p. 118-122.
124. Gupta, S.K., *Intention-to-treat concept: A review.* Perspect Clin Res, 2011. **2**(3): p. 109-12.

17. LIST OF TABLES AND FIGURES

17.1 List of tables

Table1: Overview on study visits and interventions along the study period.

Table2: Physical and chemical properties of dapagliflozin.

Table3: Study tasks and dates.

Table4: Categories of causality assessment.

17.2 List of figures

Fig.1: DAPA-VOLVO study flow chart.

Fig.2 Recurring cycle of decompensation in heart failure.

Fig.3 Cardio-renal interactions in volume expansion and congestion in chronic heart failure.

Fig.4 Paradigm of interstitial and intravascular volume expansion in chronic heart failure.

Fig.5 Heterogeneity in hypervolemic profiles in heart failure.

Fig.6 Relationship between plasma volume status and risk of heart failure hospitalization and all-cause mortality.

Fig.7 A schematic representation of the different mechanisms implicated in the cardiovascular benefits of SGLT2 inhibitors.

Fig.8 SGLT2-is treatment effect in clinically stabilized HF patients after an ADHF event- a 'volume-based' hypothesis.

Fig.9 Cardiovascular Outcomes of DAPA-HF.

Fig.10 Primary composite end point based on diabetes status and glycated hemoglobin.

Fig.11 Blood volume assessment using fully-automated optimized carbon monoxide rebreathing (OpCO) technique.

Fig.12 Retinal vessel analysis (RVA).

Fig.13 Visit 2/5 (Baseline/Final visit, time: 0d/6w): A graphical visit overview.

18. APPENDICES

N.A.
